# Sexual behavior-related exposures, sexually transmitted infections, and viral markers in relation to prostate cancer risk: a systematic review and meta-analysis

**DOI:** 10.3389/fonc.2026.1968570

**Published:** 2026-10-08

**Authors:** Po Liang, Hong Gu, Jia Liu, Yangming Liu, Liang Chen, Ping Che

**Affiliations:** 1 Department of General Practice, People's Hospital of Chongqing Hechuan, Chongqing, China; 2 Department of Urology, People's Hospital of Chongqing Hechuan, Chongqing, China; 3 Department of Urology, Chongqing Rongjun Special Care Hospital (Chongqing Southeast Hospital), Chongqing, China; 4 Department of Obstetrics, The Affiliated Banan Hospital of Chongqing Medical University (People’s Hospital of Banan District), Chongqing, China; 5 Department of Urology, People’s Hospital of Tongliang District, Chongqing, China; 6 Department of Hepatobiliary Surgery, Shapingba Hospital Affiliated to Chongqing University (Shapingba District People’s Hospital of Chongqing), Chongqing, China

**Keywords:** human papillomavirus, meta-analysis, prostate cancer, sexual behavior, sexually transmitted infections, systematic review, *Trichomonas vaginalis*, viral markers

## Abstract

**Background:**

Sexual behavior, sexually transmitted infections (STIs), and viral markers have been investigated in relation to prostate cancer, but heterogeneous exposure definitions and ascertainment methods complicate interpretation. We synthesized observational evidence separately across biologically and methodologically distinct exposure domains.

**Methods:**

We followed PRISMA 2020 and searched PubMed/MEDLINE, Embase.com, and Web of Science Core Collection from inception through 27 May 2026. Eligible studies were comparative observational studies evaluating sexual behavior-related exposures, STI history or serology, or viral markers in relation to prostate cancer. Random-effects meta-analysis was performed for sufficiently comparable estimates. Risk of bias was assessed using Newcastle-Ottawa Scale domains, supplemented by design-specific epidemiological considerations. GRADE was used as a post-registration evidence-certainty assessment.

**Results:**

Of 121 reports assessed for eligibility, 57 primary observational reports were included after final eligibility reconciliation. Twenty-five reports entered at least one pooled model and three additional reports appeared only in descriptive forest plots. For tissue- or PCR-based HPV detection, the observed pooled association was OR 3.01 (95% CI 1.97-4.61; I²=36.2%; prediction interval 1.15-7.91; 13 studies). HPV16 and HPV18 tissue/PCR subtype estimates were also elevated, whereas HPV serology showed no clear association. Estimates for Trichomonas vaginalis serology and history-based STI exposure were imprecise or heterogeneous, and sexual-behavior exposures were too heterogeneous for pooling. Certainty was very low across all evaluated exposure domains.

**Conclusions:**

Tissue- or PCR-based HPV detection was more frequent among prostate cancer cases in the available comparative studies, but the evidence is very uncertain because of risk of bias, limited temporal clarity, sampling and comparator differences, and other methodological limitations. These findings should not be interpreted as establishing an etiologic or causal effect and do not support prostate-cancer prevention recommendations based on HPV vaccination, STI screening, or sexual-behavior modification.

**Systematic review registration:**

https://www.crd.york.ac.uk/prospero/display_record.php?RecordID=1334823, identified: CRD420261334823.

## 1 Introduction

Prostate cancer is among the most common malignancies in men worldwide, with substantial geographic and socioeconomic disparities in incidence and mortality (1). While age, genetic ancestry, and family history are well-established non-modifiable risk factors, modifiable lifestyle and infectious determinants remain incompletely characterized, representing key opportunities for population-level primary prevention (2).

Chronic infection and inflammation have long been proposed as contributors to prostate carcinogenesis, through pathways including persistent epithelial injury, local immune dysregulation, oxidative DNA damage, and dysregulated host-pathogen interactions (3, 4). Human papillomavirus (HPV), particularly high-risk oncogenic subtypes, is of special interest given its established causal role in anogenital and oropharyngeal cancers (5, 6). Whether similar oncogenic mechanisms operate in prostate tissue remains epidemiologically and biologically uncertain.

Published evidence linking sexual behavior-related exposures, STIs, and viral markers to prostate cancer remains fragmented. Previous systematic reviews have examined STIs, HPV, or infectious agents, but differences in exposure ascertainment and biological meaning complicate direct comparison across studies (7–14). A synthesis that separates behavioral history, serological markers, and local tissue-based detection is therefore needed.

We treated exposure ascertainment as a core dimension of the research question. This systematic review and meta-analysis reassessed observational evidence across sexual behavior, STI history/serology, and tissue-based viral-marker domains, with the primary quantitative focus on tissue- or PCR-based HPV detection. We additionally evaluated the certainty and methodological limitations of each evidence domain to distinguish observed associations from causal inference.

## 2 Methods

### 2.1 Protocol and reporting

This systematic review was prospectively registered in PROSPERO (CRD420261334823) and is reported in accordance with the PRISMA 2020 statement (15). The PROSPERO record was amended before final quantitative synthesis to clarify title, databases, final search date, eligibility criteria, and review scope. All analyses reported here were conducted after final eligibility decisions and extraction dataset locking. The completed PRISMA 2020 checklist is provided as **Supplementary Material 4**.

The registered PROSPERO record specified Newcastle-Ottawa Scale assessment for study risk of bias and did not originally specify a formal certainty-of-evidence assessment. GRADE was added after registration as an evidence-interpretation layer. It was not part of the registered eligibility criteria and does not alter the numerical meta-analysis estimates.

### 2.2 Search strategy and information sources

PubMed/MEDLINE, Embase.com, and Web of Science Core Collection were searched from inception through 27 May 2026 without language or publication date restrictions. Search syntax combined terms for prostate cancer with terms for sexual behavior, sexually transmitted infections, HPV and other viral pathogens, serology, and tissue- or PCR-based molecular detection. Reference lists of included studies and relevant reviews were manually checked; records identified through manual search are separately indicated in the PRISMA flow diagram. Full database-specific search strategies are provided in **Supplementary Material 2**.

### 2.3 Eligibility criteria

Eligible designs included cohort, nested case-control, case-cohort, case-control, and other comparative observational studies evaluating prostate-cancer risk or case status in relation to an eligible exposure. Consistent with the registered protocol, cross-sectional studies without relevant comparative risk estimates were excluded. Comparative tissue-based studies were eligible when clearly defined prostate-cancer and non-cancer comparator groups allowed estimation of a relevant association; however, tissue-based viral detection obtained at or around diagnosis was considered unable, by itself, to establish whether viral exposure preceded carcinogenesis.

Eligible exposures included at least one measure of sexual behavior, clinically or laboratory-confirmed STI, pathogen-specific serology, or tissue- or PCR-based viral detection. Outcomes were clinically or pathologically confirmed primary prostate cancer, including total, aggressive, advanced, or fatal disease where separately reported. Reports with eligible comparative observational evidence could contribute to qualitative synthesis; quantitative or structured descriptive forest-plot analyses additionally required an adjusted OR/RR/HR with 95% CI or sufficient raw data to calculate an effect estimate.

Reviews, meta-analyses, editorials, commentaries, reports without original eligible data, duplicate or overlapping reports without additional eligible data, reports with the wrong disease or population, reports without a clear eligible comparator/reference structure where required, and reports without relevant prostate-cancer outcomes were excluded.

### 2.4 Study selection and data extraction

Records were screened in two sequential stages: title/abstract screening followed by full-text assessment. Two reviewers independently assessed full-text reports; disagreements were resolved by discussion with a senior reviewer.

Data were independently extracted and cross-checked using a standardized form. Extracted fields included study design, country, population characteristics, exposure definition and measurement method, outcome definition, effect estimate type, fully adjusted effect with 95% CI, and 2×2 contingency table data where available. For tissue- or PCR-based analyses, 2×2 data were used to calculate odds ratios where appropriate. Where multiple estimates were reported, the primary analytic estimate was selected to avoid double-counting; alternative estimates were retained for sensitivity analyses. Automation tools were not used for eligibility, extraction, or bias assessment.

### 2.5 Exposure grouping and analytic hierarchy

We separated evidence domains rather than combining all sexual or infection-related exposures. Tissue- or PCR-based HPV detection was the primary biological-detection model. HPV16/18 tissue subtypes, HPV16/18 serology, T. vaginalis serology, history-based STI, specific STI estimates, sexual behavior variables, and other viral markers were evaluated separately. OR, RR and HR were all retained as relative-effect measures; cross-type pooled analyses were explicitly labeled and interpreted cautiously.

### 2.6 Risk of bias and evidence appraisal

Risk of bias was independently appraised by two reviewers at the domain level using the relevant selection, comparability, and exposure/outcome domains of the Newcastle-Ottawa Scale, supplemented by design-specific epidemiological considerations for non-randomized evidence (18). Appraisals considered participant and comparator selection, exposure ascertainment, outcome definition, group comparability, confounding control, temporal clarity, and reporting completeness. For tissue-based molecular studies, specimen source and acquisition pathway, assay definition, contamination control, and laboratory blinding were additionally considered where reported. **Supplementary Table S2** provides report-level design, timing, comparator structure, principal risk-of-bias considerations, and interpretation for each of the 57 eligible reports. We did not sum domain judgments into a composite numeric NOS score; non-pooling was recorded separately from risk of bias.

GRADE certainty was added after registration for each principal exposure-outcome domain. Under the GRADE framework applied in this review, observational evidence was initially considered low certainty and was then evaluated across risk of bias, inconsistency, indirectness, imprecision, and publication bias, with potential upgrading factors considered where applicable. A large observed association was not considered sufficient for upgrading when serious concerns regarding bias, temporality, comparator selection, or indirectness remained.

### 2.7 Data synthesis and statistical analysis

Random-effects meta-analyses were performed when at least two studies used sufficiently similar exposure definitions and effect measures. Analyses were conducted on the logarithmic scale with inverse-variance weighting; summary estimates were back-transformed for presentation. Between-study heterogeneity was summarized with τ², Cochran’s Q test, and the I² statistic (16, 17). DerSimonian-Laird random-effects estimates were calculated using reproducible R scripts. Prediction intervals were reported where estimable.

For zero-cell tissue- or PCR-based studies, continuity-corrected odds ratios were used when necessary, with sensitivity analyses excluding zero-cell comparisons. Findings based on fewer than three studies, mixed effect measures, or non-comparable exposures were treated as exploratory or descriptive.

For the primary tissue- or PCR-based HPV model, small-study effects were explored using a funnel plot and Egger’s regression test.

Multiple-testing correction for the eight principal pooled comparisons used the Bonferroni method in the original manuscript analysis: the primary tissue- or PCR-based HPV model, HPV16 and HPV18 tissue-subtype models, HPV16 and HPV18 serology models, total and advanced/aggressive T. vaginalis models, and the history-based any-STI model. The Bonferroni two-sided threshold was P<0.00625. In response to peer review, we additionally applied the Holm step-down procedure to the same family of eight P values as a revision-stage sensitivity analysis. The Holm analysis did not replace the original manuscript Bonferroni analysis. Multiplicity-adjusted statistical significance was interpreted as evidence against the null within the defined family of tests and was not used to upgrade GRADE certainty or infer causality.

All analyses were conducted in R version 4.6.0 using the locked study-level extraction dataset. Forest plots, summary estimates, heterogeneity statistics, sensitivity analyses, and small-study-effect outputs were generated from those data. Machine-readable study-level inputs, summary tables, Egger-test output, and session information are provided in **Supplementary Material 3**.

### 2.8 Use of artificial intelligence tools

OpenAI ChatGPT Work/Codex (GPT-5-series models, including GPT-5.6; OpenAI; https://chatgpt.com; accessed August-September 2026) was used for English-language editing, document organization, plotting-code generation, figure formatting, and submission-package quality control. It was not used to determine eligibility, extract study data, assign risk-of-bias judgements, or make final scientific or statistical decisions. The plotting/statistical script uses locked study-level inputs and contains report-count, locked-result, and file-completeness gates. During revision, the authors reran the original script locally in R 4.6.0; the process exited successfully (exit code 0), reproduced the locked primary result (OR 3.01, 95% CI 1.97-4.61; I²=36.2%; prediction interval 1.15-7.91; Egger P = 0.5042), and all 20 generated figures passed the locked-result and file-completeness gates. The authors retained the validation logs and R session information and remain fully responsible for the final analysis and manuscript.

## 3 Results

### 3.1 Study selection

The final PRISMA flow diagram is shown in **Figure 1**. Searches identified 7,911 database records and 15 records through other methods. After removal of 2,579 duplicate records, 5,332 database records were screened and 5,209 were excluded before retrieval. Of 123 database reports sought for retrieval, six were not retrieved, leaving 117 database reports for eligibility assessment. Fifteen reports were sought through other methods; 11 were not retrieved and four were assessed for eligibility. Overall, 121 reports were assessed for eligibility. After final eligibility reconciliation, 64 reports were excluded and 57 eligible primary observational reports remained. Twenty-five reports entered at least one pooled model, three additional reports appeared only in descriptive forest plots (28 plotted reports in total), and 29 were retained for qualitative or contextual synthesis.

**Figure 1:**
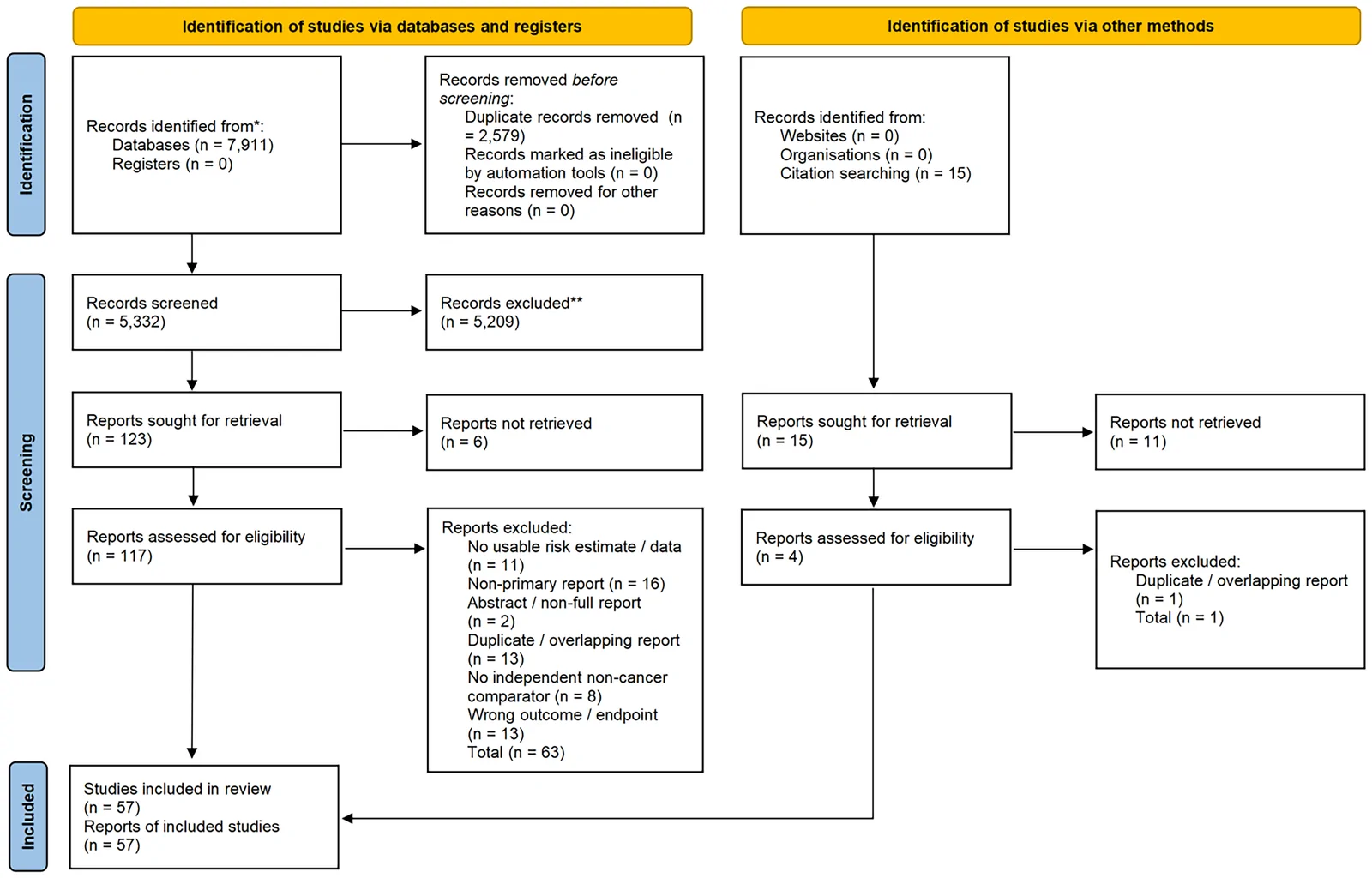
PRISMA 2020 flow diagram of study selection. Database searches identified 7,911 records and other methods identified 15 reports. Overall, 121 reports were assessed for eligibility; 64 were excluded and 57 eligible primary observational reports remained. Twenty-five reports entered at least one pooled model, three additional reports appeared only in descriptive forest plots (28 plotted reports in total), and 29 were retained for qualitative or contextual synthesis. “*” denotes records identified from databases/registers; “**” denotes records excluded at title/abstract screening.

During revision, two reports previously retained only for qualitative/contextual mapping were reclassified: Dennis LK 2002 was confirmed to be a secondary meta-analysis rather than an independent primary report, and Araujo-Neto AP 2016 did not contain the independent non-cancer comparator required by the registered eligibility criteria. Neither report had entered a pooled model or descriptive forest plot; therefore, this reconciliation did not alter any quantitative estimate, sensitivity analysis, or plotted synthesis. Primary exclusion reasons are listed in **Supplementary Table S6**.

### 3.2 Study-design structure and risk-of-bias overview

Included evidence covered distinct exposure domains: tissue- or PCR-based HPV detection, HPV serology, T. vaginalis serology, history-based STI exposure, specific STI histories, sexual behavior variables, claims-defined HPV infection, and other viral markers. Included reports and their use in synthesis are mapped in **Supplementary Table S1** and cited in the reference list (19–75).

The final evidence base comprised 57 eligible primary observational reports. At the report level, 22 (38.6%) were conventional non-tissue case-control studies, 17 (29.8%) were tissue-based case/non-cancer comparative studies, 12 (21.1%) were nested case-control studies, five (8.8%) were cohort studies, and one (1.8%) used a case-cohort design. Thus, the evidence base was dominated by case-control or contemporaneous case/non-cancer comparative evidence, whereas longitudinal cohort evidence was relatively limited. These counts refer to reports rather than necessarily independent source populations because some reports arose from common parent cohorts or study platforms.

The principal risk-of-bias concerns differed by design. Tissue-based studies were particularly limited by comparability of tissue-acquisition pathways and control specimens, contemporaneous exposure ascertainment, and laboratory-method heterogeneity. Conventional case-control studies were more susceptible to retrospective exposure reporting, participant-selection bias, and differential healthcare or PSA-related detection. Nested case-control and cohort studies generally provided stronger temporal ordering for prediagnostic serology or recorded exposure but remained susceptible to exposure misclassification, surveillance differences, and residual confounding. Report-level appraisal is provided in **Supplementary Table S2** and summarized in **Table 1**.

**Table 1:** Study-design structure and principal risk-of-bias considerations.

Study-design category	Reports, n (%)	Principal risk-of-bias considerations
Conventional non-tissue case-control studies	22 (38.6)	Case/control selection; retrospective exposure ascertainment; recall/social-desirability bias; PSA/healthcare-related detection; residual confounding
Tissue-based case/non-cancer comparative studies	17 (29.8)	Tissue-acquisition pathway and comparator comparability; contemporaneous viral detection/temporality; contamination control; assay definition/blinding; residual confounding
Nested case-control studies	12 (21.1)	Parent-cohort and control/risk-set sampling; biospecimen timing; assay thresholds; outcome ascertainment; residual confounding
Cohort studies	5 (8.8)	Exposure misclassification; PSA/surveillance differences; outcome ascertainment; follow-up; residual confounding
Case-cohort study	1 (1.8)	Subcohort representativeness and sampling structure; baseline exposure ascertainment; outcome ascertainment; residual confounding
Total	57 (100.0*)	**—**

*Percentages may not sum exactly to 100 because of rounding. Counts refer to eligible primary observational reports rather than necessarily independent source populations. Tissue-based case/non-cancer comparative reports were grouped separately for this descriptive summary irrespective of whether individual authors used case-control terminology; source-specific design descriptions are retained in **Supplementary Table S2**.

Bold values indicate the total number and percentage of eligible primary observational reports.

### 3.3 Tissue- or PCR-based HPV detection

The primary tissue- or PCR-based HPV model included 13 independent study estimates (**Figure 2**, **Table 2**). HPV detection was associated with higher odds of prostate cancer case status (OR 3.01, 95% CI 1.97-4.61; I²=36.2%; prediction interval 1.15-7.91). Its raw model P value was <0.001 and remained below the Bonferroni threshold used in the original manuscript analysis; this statistical result does not overcome the very low certainty or establish causality.

**Figure 2:**
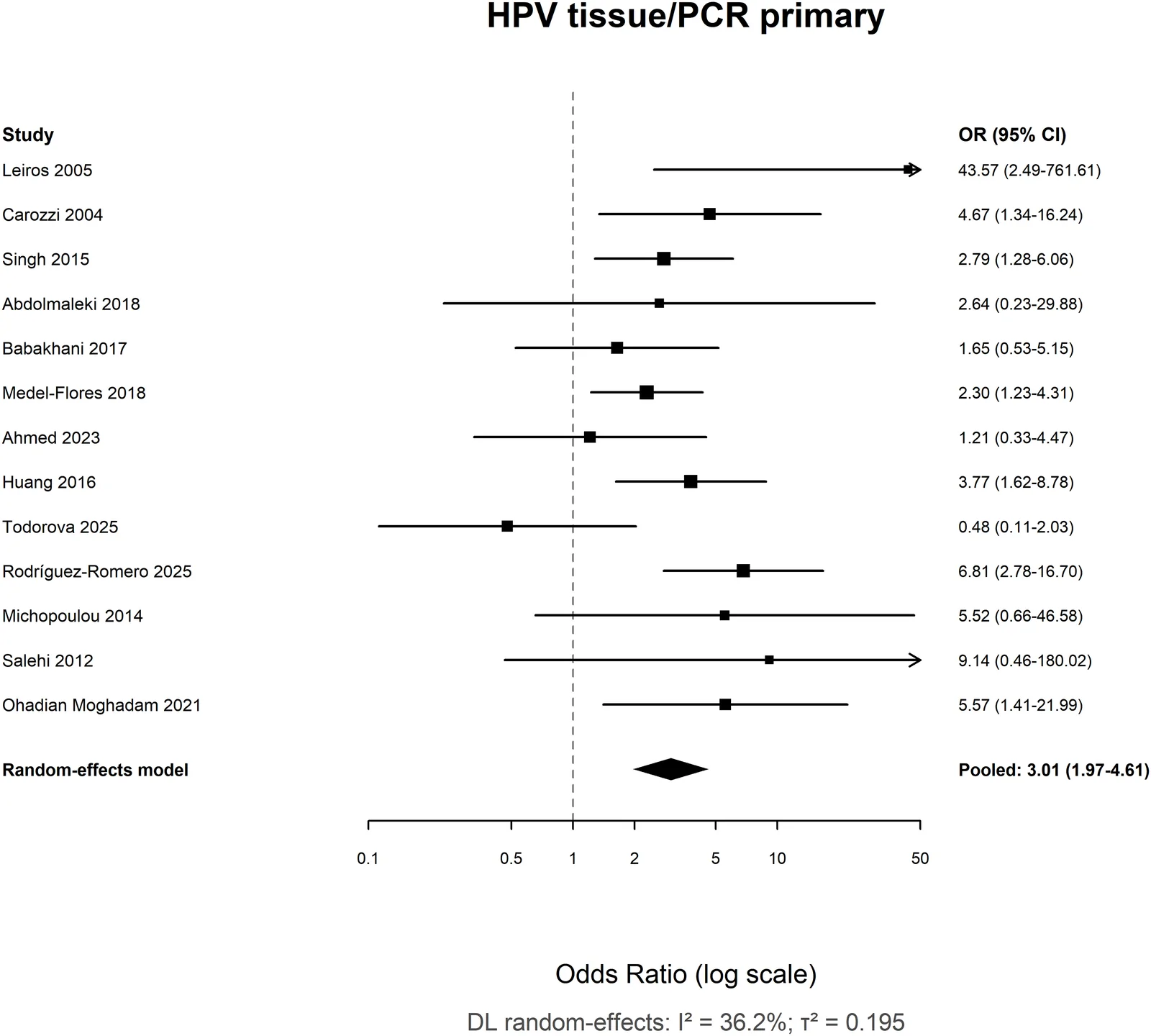
Forest plot of the association between tissue- or PCR-based HPV detection and prostate cancer case status. Random-effects meta-analysis was performed on the logarithmic odds-ratio scale. Squares are study estimates sized by relative weight; horizontal lines are 95% confidence intervals; arrows indicate intervals extending beyond the plotting range; and the diamond is the pooled estimate. The prediction interval is reported in **Table 2**.

**Table 2:** Summary of quantitative and exploratory findings.

Analysis	k	Measure	Summary estimate (95% CI)	I²	Prediction interval	Interpretation
Tissue-/PCR-based HPV, primary	13	OR	3.01 (1.97-4.61)	36.2%	1.15-7.91	Observed pooled association; very low certainty; not causal.
Tissue-/PCR-based HPV, all-zero sensitivity	14	OR	2.97 (1.97-4.49)	32.0%	1.19-7.43	Sensitivity analysis; pattern unchanged.
HPV16 tissue/PCR subtype	4	OR	4.90 (2.84-8.46)	0%	2.84-8.46	Elevated observed association; few studies; very low certainty.
HPV18 tissue/PCR subtype	4	OR	4.92 (2.44-9.93)	0%	2.44-9.93	Elevated observed association; few studies; very low certainty.
HPV16 serology	5	Relative effect	1.10 (0.85-1.43)	31.5%	0.73-1.67	No clear association.
HPV18 serology	4	Relative effect	1.20 (0.71-2.01)	62.8%	0.45-3.16	No clear association; heterogeneous.
T. vaginalis serology, total PCa	3	OR	1.37 (0.87-2.14)	70.9%	0.63-2.96	Imprecise and heterogeneous.
History-based any STI	2	OR	1.51 (0.66-3.49)	81.6%	Not estimable	Sparse and heterogeneous.
Sexual-behavior estimates	5	Relative effect	Not pooled	NA	NA	Contrasts too heterogeneous for pooling.

CI, confidence interval; HPV, human papillomavirus; NA, not applicable; OR, odds ratio; PCa, prostate cancer; PI, prediction interval; STI, sexually transmitted infection. Certainty was very low for all exposure domains. Statistical significance does not establish temporality or causality.

The primary tissue-/PCR-based HPV association remained statistically significant in the Holm sensitivity analysis (Holm-adjusted P = 2.436×10^-6^). HPV16 and HPV18 tissue/PCR subtype analyses also remained significant (adjusted P = 9.328×10^-8^ and 5.217×10^-5^, respectively), whereas the other five principal comparisons had Holm-adjusted P values ≥0.8655. The inferential pattern was therefore unchanged relative to the original manuscript Bonferroni analysis. These multiplicity-adjusted results address statistical evidence against the null hypothesis only and do not increase evidence certainty or establish temporality or causality.

The estimate was materially similar across sensitivity analyses: including an all-zero HPV detection study yielded OR 2.97 (95% CI 1.97-4.49; I²=32.0%), excluding the healthy-tissue comparator study yielded OR 2.95 (95% CI 1.90-4.60), and excluding zero-cell comparisons yielded OR 2.80 (95% CI 1.87-4.20).

Funnel plot (**Supplementary Figure S19**) did not show clear asymmetry, and Egger’s regression test provided no clear evidence of small-study effects (intercept 0.59, 95% CI -1.29 to 2.47; P = 0.504), though statistical power was limited by the modest number of studies.

### 3.4 HPV subtype and serology analyses

HPV16 and HPV18 tissue- or PCR-based subtype analyses also showed elevated estimates. The HPV16 model included four studies and yielded OR 4.90 (95% CI 2.84-8.46; I²=0%). The HPV18 model included four studies and yielded OR 4.92 (95% CI 2.44-9.93; I²=0%), with similar findings in sensitivity analyses.

In contrast, HPV serology was not clearly associated with prostate cancer. HPV16 serology produced a pooled relative effect of 1.10 (95% CI 0.85-1.43; I²=31.5%), and HPV18 serology produced 1.20 (95% CI 0.71-2.01; I²=62.8%). Neither association remained statistically significant after multiplicity adjustment.

### 3.5 Trichomonas vaginalis serology, STI history, and sexual behavior

T. vaginalis serology was evaluated in three total-prostate-cancer estimates and produced an imprecise and heterogeneous estimate (OR 1.37, 95% CI 0.87-2.14; I²=70.9%; prediction interval 0.63-2.96). Advanced/aggressive disease estimates were sparse and imprecise.

Self-reported any-STI exposure was based on two studies and also imprecise (OR 1.51, 95% CI 0.66-3.49; I²=81.6%).

Specific STI estimates, sexual behavior-related estimates, and other infection-related estimates were not pooled because exposure definitions and effect measures differed substantially. Descriptive forest plots are provided in the **Supplementary Materials**.

### 3.6 GRADE evidence certainty summary

Certainty was very low across all evaluated exposure domains. For tissue-/PCR-based HPV detection, major concerns were risk of bias and indirectness related particularly to comparator selection, tissue sampling, and inability to establish temporal sequence; inconsistency was more limited in the primary model, but uncertainty remained across settings. HPV16/18 tissue-subtype evidence was very low because of risk-of-bias and indirectness concerns and substantial imprecision arising from the small evidence base. HPV serology evidence was very low because of combinations of risk of bias, imprecision, inconsistency, and indirectness of systemic serological markers for local prostatic infection. T. vaginalis and history-based STI evidence was also very low because of combinations of risk of bias, inconsistency, indirectness, and imprecision. Accordingly, pooled estimates are interpreted as summary associations rather than reliable causal effect estimates. Full domain-level judgments are provided in **Supplementary Table S5**.

## 4 Discussion

### 4.1 Principal findings

The evidence did not show a uniform pattern across infection-related and sexual behavior-related exposure domains. Tissue- or PCR-based HPV detection showed the largest observed pooled association, but certainty in this estimate was very low. The pooled value should therefore be interpreted as a summary of the observed association across the included comparative studies rather than as a reliable etiologic effect size. In particular, most contributing studies could not establish whether viral detection preceded prostate carcinogenesis. HPV serology, T. vaginalis serology, history-based STI exposure, and sexual-behavior measures showed weaker, inconsistent, imprecise, or non-poolable patterns. These differences support domain-specific interpretation according to exposure ascertainment rather than a single omnibus estimate for infection or sexual exposure.

### 4.2 Interpretation of tissue-based and serological evidence

Tissue- or PCR-based assays detect viral DNA directly in prostate specimens and therefore reflect local viral presence more closely than systemic serology. Even so, tissue-based findings remain vulnerable to multiple biases, including case-control sampling, choice of benign hyperplasia or normal tissue controls, fixation and DNA quality, contamination control, assay sensitivity, and selective genotype reporting.

The absence of a clear serological signal does not directly refute tissue-based findings, because serology measures past systemic immune response rather than current local prostatic infection. Conversely, tissue- or PCR-based positivity alone cannot establish temporal sequence, persistent infection, or biological causation. The divergent pattern between tissue and serological evidence highlights why combining different exposure measurement methods can be misleading.

### 4.3 PSA screening, tissue sampling, and detection bias

Detection and verification processes are important potential sources of bias in prostate-cancer epidemiology. PSA testing, clinical indications for biopsy, lower urinary tract symptoms, benign prostatic enlargement, healthcare utilization, and acceptance of biopsy can influence whether otherwise occult prostate cancer is detected. These mechanisms are relevant when observational exposure groups differ in contact with healthcare or likelihood of diagnostic work-up.

The same concern is especially important for tissue-based HPV studies. Prostate tissue is not sampled independently of clinical indication: cancer cases and non-cancer comparators may undergo biopsy, transurethral resection, or surgery for different reasons, and benign prostatic hyperplasia is frequently used as a source of comparison tissue. Consequently, cases and comparators may differ not only in cancer status but also in the probability, anatomical source, and clinical context of tissue acquisition. Such selection and verification processes can distort observed tissue-based associations, but their direction and magnitude cannot be assumed *a priori*.

Accordingly, the observed tissue-/PCR-based HPV association should not be interpreted as an unbiased causal effect estimate. Future studies should prospectively define tissue-acquisition pathways, use comparable sampling procedures in cases and controls, document PSA and biopsy indications, incorporate healthcare-utilization measures, and establish exposure before cancer diagnosis whenever feasible.

Broader lifestyle and metabolic factors may also shape prostate-cancer epidemiology and the context in which disease is detected. Physical activity has been reviewed in relation to prostate carcinogenesis and clinical outcomes, while recent observational work has reported a positive association between body mass index and prostate volume (76, 77). These factors were outside the registered exposure scope of the present review and were not included in the evidence synthesis, but they reinforce the need to consider lifestyle, metabolic, and detection-related pathways when interpreting observational associations.

### 4.4 Comparison with previous reviews

Previous syntheses reported mixed or modest associations between STIs, HPV, and prostate cancer (7–14). The present review differs from earlier summaries by explicitly separating tissue-based detection, serological exposure, STI history, and sexual behavior into distinct evidence domains. This methodological separation helps explain why tissue-based HPV studies can show an association while serological studies do not, and it anchors interpretation firmly to the quality and type of evidence. The updated search to May 2026 further adds more recent population-based and molecular studies.

### 4.5 Public health implications

Current evidence is insufficient to recommend any prostate-cancer prevention intervention based on these associations. HPV vaccination, STI prevention, sexual-health education, and indicated STI testing remain important for their established benefits, but this review does not demonstrate that they prevent prostate cancer. The tissue-detection association instead identifies a research priority: prospective studies should establish temporality, standardize laboratory contamination controls and assay definitions, and measure PSA screening and healthcare use. Linkage of vaccination, infection, screening, and cancer-registry data could test long-term hypotheses, provided that privacy, consent, and data-governance requirements are met.

### 4.6 Strengths and limitations

Key strengths include the updated literature search through May 2026, prospective PROSPERO registration, PRISMA reporting, explicit separation of exposure-measurement domains, report-level traceability, comprehensive sensitivity analyses, and transparent domain-level risk-of-bias and GRADE assessments. The revision also reconciled eligibility against the registered comparator and primary-report criteria without changing any quantitative estimate.

Important limitations remain. All included studies were observational, so residual confounding cannot be excluded and causality cannot be established. Exposure definitions, comparator groups, tissue sources, laboratory assays, outcome definitions, and confounder adjustment varied across studies. PSA testing, healthcare utilization, biopsy indications, and tissue-acquisition pathways were often incompletely measured, creating potential selection and detection/verification bias whose direction cannot be assumed. Several pooled domains contained few studies, limiting precision and the ability to investigate heterogeneity. Although funnel-plot and Egger testing did not identify clear small-study effects for the primary HPV model, power was limited and selective reporting cannot be excluded. Most data originated from upper-middle- and high-income settings, which may limit generalizability. Multiplicity correction addresses statistical testing but does not correct bias, confounding, indirectness, or imprecision. Finally, GRADE was added after registration as an interpretive layer rather than as a registered protocol component; this addition did not alter eligibility decisions or numerical meta-analysis estimates.

## 5 Conclusions

The available literature does not establish a uniform association between sexual behavior-related exposures, STIs, viral markers, and prostate-cancer risk. Tissue- or PCR-based HPV detection showed an observed pooled association with prostate-cancer case status, but certainty is very low and the available evidence cannot establish temporal sequence or causality. Serological and history-based evidence was weaker, heterogeneous, or imprecise. Future work should prioritize prospective exposure ascertainment, comparable tissue-sampling pathways, standardized laboratory methods, and measurement of PSA screening and healthcare utilization. The current evidence does not support prostate-cancer prevention recommendations based specifically on HPV vaccination, STI screening, or sexual-behavior modification.

## Data Availability

The original contributions presented in the study are included in the article/**Supplementary Material**. Further inquiries can be directed to the corresponding author.

## Supplementary material

Supplementary Figure 1 Sensitivity analysis for tissue- or PCR-based HPV detection including the all-zero HPV detection study.

Supplementary Figure 2 Sensitivity analysis excluding the study using a healthy-tissue comparator.

Supplementary Figure 3 Sensitivity analysis excluding zero-cell tissue- or PCR-based studies or comparisons.

Supplementary Figure 4 Forest plot for HPV16 tissue- or PCR-based subtype detection.

Supplementary Figure 5 Forest plot for the primary HPV18 tissue- or PCR-based subtype analysis.

Supplementary Figure 6 Alternative HPV18 tissue- or PCR-based subtype analysis using the alternative Ohadian Moghadam data extraction.

Supplementary Figure 7 HPV18 tissue- or PCR-based subtype sensitivity analysis excluding the source-discrepant Ohadian Moghadam row.

Supplementary Figure 8 HPV16 serology mixed relative-effect analysis.

Supplementary Figure 9 HPV16 serology OR-only sensitivity analysis.

Supplementary Figure 10 HPV18 serology mixed relative-effect analysis.

Supplementary Figure 11 HPV18 serology OR-only sensitivity analysis.

Supplementary Figure 12 T. vaginalis serology and total prostate cancer primary analysis.

Supplementary Figure 13 T. vaginalis serology sensitivity analysis using the alternative Saleh denominator.

Supplementary Figure 14 T. vaginalis serology and advanced/aggressive prostate cancer analysis.

Supplementary Figure 15 Exploratory analysis of history-based or self-reported any-STI exposure.

Supplementary Figure 16 Descriptive forest plot of specific STI estimates.

Supplementary Figure 17 Descriptive forest plot of sexual behavior-related estimates.

Supplementary Figure 18 Descriptive forest plot of other infection-related estimates.

Supplementary Figure 19 Funnel plot for the primary tissue- or PCR-based HPV model.

Supplementary Material 1 Supplementary Tables S1-S6 (.docx): Study/report traceability, risk-of-bias appraisal, meta-analysis summaries, study-level forest-plot inputs, evidence-certainty summaries, and full-text exclusion reasons.

Supplementary Material 2 Full Search Strategies (.pdf): Database-specific Embase.com, PubMed/MEDLINE, and Web of Science Core Collection strategies, final search dates, and supplementary search methods.

Supplementary Material 3 Supplementary Figures and Meta-analysis Data (.zip): Supplementary forest plots S1-S18, funnel plot S19, combined supplementary-figure PDF, machine-readable study-level inputs and summary tables, analytic R code, Egger-test output, and R session information.

Supplementary Material 4 PRISMA 2020 Checklist (.docx): Completed PRISMA 2020 checklist mapped to the revised manuscript and supplementary files.
