# Supplementary material for: Sexual behavior-related exposures, sexually transmitted infections, and viral markers in relation to prostate cancer risk: a systematic review and meta-analysis
Source: Front Oncol. 2026 Oct 8;16:1968570. doi: 10.3389/fonc.2026.1968570 (PMC13686856; doi:10.3389/fonc.2026.1968570)
Supplement: Supplementary file 1 [file DataSheet1.pdf]

Full Search Strategies

Sexual behavior-related exposures, sexually transmitted infections, and viral markers in relation to prostate cancer risk: a systematic review and meta-analysis

PROSPERO registration: CRD420261334823

The final database searches were conducted on 27 May 2026. No language or publication-date restrictions and no study-design filter were applied at the search stage.

Database	Platform / interface	Final search date
Embase	Embase.com (institutional/proxy access)	27 May 2026
PubMed	PubMed/MEDLINE	27 May 2026
Web of Science	Web of Science Core Collection	27 May 2026

1. Embase (Embase.com)

Search string used/adapted for Embase.com syntax:

```
('prostate cancer'/exp OR 'prostate cancer':ti,ab,kw OR 'prostatic cancer':ti,ab,kw OR 'prostate carcinoma':ti,ab,kw
OR 'prostatic carcinoma':ti,ab,kw OR 'prostate neoplasm*':ti,ab,kw OR 'prostatic neoplasm*':ti,ab,kw OR 'prostate
tumor*':ti,ab,kw OR 'prostate tumour*':ti,ab,kw)
AND
('sexual behavior'/exp OR 'sexual activity'/exp OR 'ejaculation'/exp OR 'sexually transmitted disease'/exp OR '
papillomavirus infection'/exp OR 'human papillomavirus'/exp OR 'herpesvirus infection'/exp OR 'human herpesvirus
8'/exp OR 'cytomegalovirus infection'/exp OR 'epstein barr virus infection'/exp OR 'sexual behavio*':ti,ab,kw OR '
sexual activ*':ti,ab,kw OR intercourse:ti,ab,kw OR 'sexual partner*':ti,ab,kw OR 'number of partner*':ti,ab,kw OR
condom*:ti,ab,kw OR 'commercial sex':ti,ab,kw OR prostitution:ti,ab,kw OR 'sex work*':ti,ab,kw OR 'age at first
intercourse':ti,ab,kw OR 'premarital sex':ti,ab,kw OR ejacul*:ti,ab,kw OR masturbation:ti,ab,kw OR 'nocturnal
emission*':ti,ab,kw OR 'sexually transmitted infection*':ti,ab,kw OR 'sexually transmitted disease*':ti,ab,kw OR
STI:ti,ab,kw OR STIs:ti,ab,kw OR STD:ti,ab,kw OR STDs:ti,ab,kw OR gonorrhea:ti,ab,kw OR gonorrhoea:ti,ab,kw OR
syphilis:ti,ab,kw OR chlamydia:ti,ab,kw OR 'Chlamydia trachomatis':ti,ab,kw OR trichomon*:ti,ab,kw OR 'Trichomonas
vaginalis':ti,ab,kw OR herpes:ti,ab,kw OR 'genital herpes':ti,ab,kw OR HSV:ti,ab,kw OR HPV:ti,ab,kw OR 'human
papillomavirus':ti,ab,kw OR papillomavirus:ti,ab,kw OR HPV16:ti,ab,kw OR HPV18:ti,ab,kw OR 'human herpesvirus
8':ti,ab,kw OR HHV-8:ti,ab,kw OR KSHV:ti,ab,kw OR 'Kaposi sarcoma-associated herpesvirus':ti,ab,kw OR 'human
herpesvirus 6':ti,ab,kw OR HHV-6:ti,ab,kw OR cytomegalovirus:ti,ab,kw OR CMV:ti,ab,kw OR 'Epstein-Barr':ti,ab,kw OR
EBV:ti,ab,kw OR serolog*:ti,ab,kw OR seropositiv*:ti,ab,kw OR antibody:ti,ab,kw OR antibodies:ti,ab,kw OR 'viral
DNA':ti,ab,kw OR 'viral RNA':ti,ab,kw OR PCR:ti,ab,kw OR qPCR:ti,ab,kw OR 'polymerase chain reaction':ti,ab,kw OR
immunohistochemistry:ti,ab,kw OR IHC:ti,ab,kw OR 'in situ hybridization':ti,ab,kw OR ISH:ti,ab,kw)
```

2. PubMed/MEDLINE

Search string:

```
("Prostatic Neoplasms"[Mesh] OR "prostate cancer"[tiab] OR "prostatic cancer"[tiab] OR "prostate carcinoma"[tiab] OR "
prostatic carcinoma"[tiab] OR "prostate neoplasm*"[tiab] OR "prostatic neoplasm*"[tiab] OR "prostate tumor*"[tiab]
OR "prostate tumour*"[tiab])
AND
("Sexual Behavior"[Mesh] OR "Sexual Health"[Mesh] OR "Ejaculation"[Mesh] OR "Sexually Transmitted Diseases"[Mesh] OR "
Papillomavirus Infections"[Mesh] OR "Herpesviridae Infections"[Mesh] OR "Herpesvirus 8, Human"[Mesh] OR "
Cytomegalovirus Infections"[Mesh] OR "Epstein-Barr Virus Infections"[Mesh] OR sexual behavio*[tiab] OR sexual
activ*[tiab] OR intercourse[tiab] OR "sexual partner*"[tiab] OR "number of partner*"[tiab] OR condom*[tiab] OR "
commercial sex"[tiab] OR prostitution[tiab] OR "sex work*"[tiab] OR "age at first intercourse"[tiab] OR "premarital
sex"[tiab] OR ejacul*[tiab] OR masturbation[tiab] OR "nocturnal emission*"[tiab] OR "sexually transmitted
infection*"[tiab] OR "sexually transmitted disease*"[tiab] OR STI[tiab] OR STIs[tiab] OR STD[tiab] OR STDs[tiab] OR
gonorrhea[tiab] OR gonorrhoea[tiab] OR syphilis[tiab] OR chlamydia[tiab] OR "Chlamydia trachomatis"[tiab] OR
trichomon*[tiab] OR "Trichomonas vaginalis"[tiab] OR herpes[tiab] OR "genital herpes"[tiab] OR HSV[tiab] OR
HPV[tiab] OR "human papillomavirus"[tiab] OR papillomavirus[tiab] OR HPV16[tiab] OR HPV18[tiab] OR "human
herpesvirus 8"[tiab] OR HHV-8[tiab] OR KSHV[tiab] OR "Kaposi sarcoma-associated herpesvirus"[tiab] OR "human
herpesvirus 6"[tiab] OR HHV-6[tiab] OR cytomegalovirus[tiab] OR CMV[tiab] OR "Epstein-Barr"[tiab] OR EBV[tiab] OR
serolog*[tiab] OR seropositiv*[tiab] OR antibody[tiab] OR antibodies[tiab] OR "viral DNA"[tiab] OR "viral RNA"[tiab]
OR PCR[tiab] OR qPCR[tiab] OR "polymerase chain reaction"[tiab] OR immunohistochemistry[tiab] OR IHC[tiab] OR "in
situ hybridization"[tiab] OR ISH[tiab])
```

3. Web of Science Core Collection

Search string:

```
("prostate cancer" OR "prostatic cancer" OR "prostate carcinoma" OR "prostatic carcinoma" OR "prostate neoplasm*" OR "
prostatic neoplasm*" OR "prostate tumor*" OR "prostate tumour*")
AND
("sexual behavio*" OR "sexual activ*" OR intercourse OR "sexual partner*" OR "number of partner*" OR condom* OR "
commercial sex" OR prostitution OR "sex work*" OR "age at first intercourse" OR "premarital sex" OR ejacul* OR
masturbation OR "nocturnal emission*" OR "sexually transmitted infection*" OR "sexually transmitted disease*" OR STI
OR STIs OR STD OR STDs OR gonorrhea OR gonorrhoea OR syphilis OR chlamydia OR "Chlamydia trachomatis" OR trichomon*
OR "Trichomonas vaginalis" OR herpes OR "genital herpes" OR HSV OR HPV OR "human papillomavirus" OR papillomavirus
OR HPV16 OR HPV18 OR "human herpesvirus 8" OR HHV-8 OR KSHV OR "Kaposi sarcoma-associated herpesvirus" OR "human
herpesvirus 6" OR HHV-6 OR cytomegalovirus OR CMV OR "Epstein-Barr" OR EBV OR serolog* OR seropositiv* OR antibody
OR antibodies OR "viral DNA" OR "viral RNA" OR PCR OR qPCR OR "polymerase chain reaction" OR immunohistochemistry OR
IHC OR "in situ hybridization" OR ISH)
```

Supplementary search methods

Reference lists of included studies and relevant reviews were screened, and forward citation tracking was used to identify additional eligible reports. Unpublished studies were not systematically searched.

Eligibility notes for screening

Records were screened for observational evidence evaluating prostate cancer risk in relation to sexual behavior-related exposures, including ejaculation or sexual-activity frequency, self-reported STI history, serology-defined pathogen exposure, and tissue-based viral detection. Reports were retained for qualitative synthesis when they provided relevant observational evidence; final plotted analyses additionally required a comparator group and an extractable effect estimate or sufficient raw data.
