# Supplementary material for: Sexual behavior-related exposures, sexually transmitted infections, and viral markers in relation to prostate cancer risk: a systematic review and meta-analysis
Source: Front Oncol. 2026 Oct 8;16:1968570. doi: 10.3389/fonc.2026.1968570 (PMC13686856; doi:10.3389/fonc.2026.1968570)
Supplement: Supplementary file 2 [file Table1.docx]

**Supplementary Tables for Submission**

**Sexual behavior-related exposures, sexually transmitted infections, and viral markers in relation to prostate cancer risk: a systematic review and meta-analysis**

These supplementary tables provide study-level traceability, risk-of-bias appraisal, meta-analysis summary results, study-level forest-plot inputs, evidence-certainty summaries, and full-text exclusion reasons for the review.

**Supplementary Table S1. Included-study/report traceability table and reference audit**

| **Ref. no.** | **Study/report** | **Evidence domain(s)** | **Use in synthesis** | **Linked figure(s)** | **Traceability note** |
| --- | --- | --- | --- | --- | --- |
| 19 | McNicol PJ 1991 | HPV tissue/PCR detection | Qualitative/contextual synthesis; not included in final plotted analyses. | Qualitative / not in final plot | Verified against the final inclusion and exclusion reconciliation; retained for qualitative synthesis only and not represented in the final forest plots. |
| 20 | Strickler HD 1998 | HPV tissue/PCR detection; HPV/viral marker serology | Supplementary quantitative/descriptive analysis | Supplementary Figure S1 | Mapped to the final analysis output. |
| 21 | Carozzi F 2004 | HPV tissue/PCR detection | Main meta-analysis; Supplementary quantitative/descriptive analysis | Figure 2; Supplementary Figure S1; Supplementary Figure S2; Supplementary Figure S3 | Mapped to the final analysis output. |
| 22 | Leiros GJ 2005 | HPV tissue/PCR detection | Main meta-analysis; Supplementary quantitative/descriptive analysis | Figure 2; Supplementary Figure S1; Supplementary Figure S2 | Mapped to the final analysis output. |
| 23 | Salehi Z 2012 | HPV tissue/PCR detection | Main meta-analysis; Supplementary quantitative/descriptive analysis | Figure 2; Supplementary Figure S1; Supplementary Figure S2 | Mapped to the final analysis output. |
| 24 | Michopoulou V 2014 | HPV tissue/PCR detection | Main meta-analysis; Supplementary quantitative/descriptive analysis | Figure 2; Supplementary Figure S1; Supplementary Figure S3 | Mapped to the final analysis output. |
| 25 | Singh N 2015 | HPV tissue/PCR detection; HPV16/18 tissue subtype | Main meta-analysis; Supplementary quantitative/descriptive analysis | Figure 2; Supplementary Figure S1; Supplementary Figure S2; Supplementary Figure S3; Supplementary Figure S4; Supplementary Figure S5; Supplementary Figure S6; Supplementary Figure S7 | Mapped to the final analysis output. |
| 26 | Huang L 2016 | HPV tissue/PCR detection; HPV16/18 tissue subtype | Main meta-analysis; Supplementary quantitative/descriptive analysis | Figure 2; Supplementary Figure S1; Supplementary Figure S2; Supplementary Figure S3; Supplementary Figure S4; Supplementary Figure S5; Supplementary Figure S6; Supplementary Figure S7 | Mapped to the final analysis output. |
| 27 | Babakhani F 2017 | HPV tissue/PCR detection | Main meta-analysis; Supplementary quantitative/descriptive analysis | Figure 2; Supplementary Figure S1; Supplementary Figure S2; Supplementary Figure S3 | Mapped to the final analysis output. |
| 28 | Abdolmaleki N 2018 | HPV tissue/PCR detection | Main meta-analysis; Supplementary quantitative/descriptive analysis | Figure 2; Supplementary Figure S1; Supplementary Figure S2; Supplementary Figure S3 | Mapped to the final analysis output. |
| 29 | Medel-Flores O 2018 | HPV tissue/PCR detection | Main meta-analysis; Supplementary quantitative/descriptive analysis | Figure 2; Supplementary Figure S1; Supplementary Figure S2; Supplementary Figure S3 | Mapped to the final analysis output. |
| 30 | Ohadian Moghadam S 2021 | HPV tissue/PCR detection; HPV16/18 tissue subtype | Main meta-analysis; Supplementary quantitative/descriptive analysis | Figure 2; Supplementary Figure S1; Supplementary Figure S2; Supplementary Figure S3; Supplementary Figure S4; Supplementary Figure S5; Supplementary Figure S6 | Mapped to the final analysis output. |
| 31 | Ahmed MY 2023 | HPV tissue/PCR detection | Main meta-analysis; Supplementary quantitative/descriptive analysis | Figure 2; Supplementary Figure S1; Supplementary Figure S2; Supplementary Figure S3 | Mapped to the final analysis output. |
| 32 | Todorova E 2025 | HPV/EBV tissue detection | Main meta-analysis; Supplementary quantitative/descriptive analysis | Figure 2; Supplementary Figure S1; Supplementary Figure S2; Supplementary Figure S3 | Mapped to the final analysis output. |
| 33 | Rodríguez-Romero BI 2025 | HPV tissue/PCR detection; HPV16/18 tissue subtype | Main meta-analysis; Supplementary quantitative/descriptive analysis | Figure 2; Supplementary Figure S1; Supplementary Figure S2; Supplementary Figure S3; Supplementary Figure S4; Supplementary Figure S5; Supplementary Figure S6; Supplementary Figure S7 | Mapped to the final analysis output. |
| 34 | Dillner J 1998 | HPV serology | Supplementary quantitative/descriptive analysis | Supplementary Figure S10; Supplementary Figure S8 | Mapped to the final analysis output. |
| 35 | Hisada M 2000 | HPV16 serology | Supplementary quantitative/descriptive analysis | Supplementary Figure S8; Supplementary Figure S9 | Mapped to the final analysis output. |
| 36 | Rosenblatt KA 2003 | HPV16/18 serology; sexual factors | Supplementary quantitative/descriptive analysis | Supplementary Figure S10; Supplementary Figure S11; Supplementary Figure S8; Supplementary Figure S9 | Mapped to the final analysis output. |
| 37 | Korodi Z 2005 | HPV16/18 serology | Supplementary quantitative/descriptive analysis | Supplementary Figure S10; Supplementary Figure S11; Supplementary Figure S8; Supplementary Figure S9 | Mapped to the final analysis output. |
| 38 | Sutcliffe S 2010 | HPV16/18 serology | Supplementary quantitative/descriptive analysis | Supplementary Figure S10; Supplementary Figure S11; Supplementary Figure S8; Supplementary Figure S9 | Mapped to the final analysis output. |
| 39 | Sutcliffe S 2006 | T. vaginalis serology; STI history | Supplementary quantitative/descriptive analysis | Supplementary Figure S12; Supplementary Figure S13 | Mapped to the final analysis output. |
| 40 | Stark JR 2009 | T. vaginalis serology | Qualitative/contextual synthesis; not included in final plotted analyses. | Qualitative / not in final plot | Verified against the final inclusion and exclusion reconciliation; retained for qualitative synthesis only and not represented in the final forest plots. |
| 41 | Fowke JH 2016 | T. vaginalis serology | Qualitative/contextual synthesis; not included in final plotted analyses. | Qualitative / not in final plot | Verified against the final inclusion and exclusion reconciliation; retained for qualitative synthesis only and not represented in the final forest plots. |
| 42 | Shui IM 2016 | T. vaginalis serology; aggressive/advanced PCa | Supplementary quantitative/descriptive analysis | Supplementary Figure S14 | Mapped to the final analysis output. |
| 43 | Marous M 2017 | T. vaginalis serology | Supplementary quantitative/descriptive analysis | Supplementary Figure S12; Supplementary Figure S13; Supplementary Figure S14 | Mapped to the final analysis output. |
| 44 | Saleh NE 2021 | T. vaginalis serology | Supplementary quantitative/descriptive analysis | Supplementary Figure S12; Supplementary Figure S13 | Mapped to the final analysis output. |
| 45 | Sawaya M 2024 | History-based STI; specific STI; other infection markers | Supplementary quantitative/descriptive analysis | Supplementary Figure S15; Supplementary Figure S16; Supplementary Figure S18 | Mapped to the final analysis output. |
| 46 | Ángeles-Garay U 2019 | History-based STI; sexual behavior | Supplementary quantitative/descriptive analysis | Supplementary Figure S15; Supplementary Figure S17 | Mapped to the final analysis output. |
| 47 | Mandel JS 1987 | Sexual behavior | Supplementary quantitative/descriptive analysis | Supplementary Figure S17 | Mapped to the final analysis output. |
| 48 | Honda GD 1988 | Sexual behavior | Supplementary quantitative/descriptive analysis | Supplementary Figure S17 | Mapped to the final analysis output. |
| 49 | Hayes RB 2000 | Sexual behavior; STI history | Qualitative/contextual synthesis; not included in final plotted analyses. | Qualitative / not in final plot | Verified against the final inclusion and exclusion reconciliation; retained for qualitative synthesis only and not represented in the final forest plots. |
| 50 | Sarma AV 2006 | Sexual behavior; STI history; prostatitis | Qualitative/contextual synthesis; not included in final plotted analyses. | Qualitative / not in final plot | Verified against the final inclusion and exclusion reconciliation; retained for qualitative synthesis only and not represented in the final forest plots. |
| 51 | Hennis AJM 2013 | Sexual behavior; urologic characteristics | Qualitative/contextual synthesis; not included in final plotted analyses. | Qualitative / not in final plot | Verified against the final inclusion and exclusion reconciliation; retained for qualitative synthesis only and not represented in the final forest plots. |
| 52 | Leitzmann MF 2004 | Ejaculation frequency | Qualitative/contextual synthesis; not included in final plotted analyses. | Qualitative / not in final plot | Verified against the final inclusion and exclusion reconciliation; retained for qualitative synthesis only and not represented in the final forest plots. |
| 53 | Rider JR 2016 | Ejaculation frequency | Qualitative/contextual synthesis; not included in final plotted analyses. | Qualitative / not in final plot | Verified against the final inclusion and exclusion reconciliation; retained for qualitative synthesis only and not represented in the final forest plots. |
| 54 | Papa NP 2017 | Ejaculation frequency; aggressive PCa | Qualitative/contextual synthesis; not included in final plotted analyses. | Qualitative / not in final plot | Verified against the final inclusion and exclusion reconciliation; retained for qualitative synthesis only and not represented in the final forest plots. |
| 55 | Nair-Shalliker V 2017 | Sexual history; adolescent sexual development | Qualitative/contextual synthesis; not included in final plotted analyses. | Qualitative / not in final plot | Verified against the final inclusion and exclusion reconciliation; retained for qualitative synthesis only and not represented in the final forest plots. |
| 56 | Lozano-Lorca M 2023 | Ejaculation frequency | Qualitative/contextual synthesis; not included in final plotted analyses. | Qualitative / not in final plot | Verified against the final inclusion and exclusion reconciliation; retained for qualitative synthesis only and not represented in the final forest plots. |
| 57 | McDonald AC 2011 | HHV-8 serology | Supplementary quantitative/descriptive analysis | Supplementary Figure S18 | Mapped to the final analysis output. |
| 58 | Yin SH 2024 | Claims-defined HPV infection | Qualitative/contextual synthesis; not included in final plotted analyses. | Qualitative / not in final plot | Verified against the final inclusion and exclusion reconciliation; retained for qualitative synthesis only and not represented in the final forest plots. |
| 59 | Aghakhani A 2011 | HPV tissue detection | Qualitative/contextual synthesis; not included in final plotted analyses. | Qualitative / not in final plot | Verified against the final inclusion and exclusion reconciliation; retained for qualitative synthesis only and not represented in the final forest plots. |
| 60 | Whitaker NJ 2013 | HPV/EBV tissue detection | Qualitative/contextual synthesis; not included in final plotted analyses. | Qualitative / not in final plot | Verified against the final inclusion and exclusion reconciliation; retained for qualitative synthesis only and not represented in the final forest plots. |
| 61 | Cheng I 2010 | STI history; prostatitis | Qualitative/contextual synthesis; not included in final plotted analyses. | Qualitative / not in final plot | Verified against the final inclusion and exclusion reconciliation; retained for qualitative synthesis only and not represented in the final forest plots. |
| 62 | Chung SD 2013 | Claims-defined STI history | Qualitative/contextual synthesis; not included in final plotted analyses. | Qualitative / not in final plot | Verified against the final inclusion and exclusion reconciliation; retained for qualitative synthesis only and not represented in the final forest plots. |
| 63 | Dennis LK 2009 | STI serology | Qualitative/contextual synthesis; not included in final plotted analyses. | Qualitative / not in final plot | Verified against the final inclusion and exclusion reconciliation; retained for qualitative synthesis only and not represented in the final forest plots. |
| 64 | Wang YC 2017 | Claims-defined gonorrhea | Qualitative/contextual synthesis; not included in final plotted analyses. | Qualitative / not in final plot | Verified against the final inclusion and exclusion reconciliation; retained for qualitative synthesis only and not represented in the final forest plots. |
| 65 | Vázquez-Salas RA 2016 | Gonorrhea/STI history; sexual behavior | Qualitative/contextual synthesis; not included in final plotted analyses. | Qualitative / not in final plot | Verified against the final inclusion and exclusion reconciliation; retained for qualitative synthesis only and not represented in the final forest plots. |
| 66 | Anttila T 2005 | Chlamydia serology | Qualitative/contextual synthesis; not included in final plotted analyses. | Qualitative / not in final plot | Verified against the final inclusion and exclusion reconciliation; retained for qualitative synthesis only and not represented in the final forest plots. |
| 67 | Sutcliffe S 2007 | Chlamydia/HPV/HHV-8 serology | Qualitative/contextual synthesis; not included in final plotted analyses. | Qualitative / not in final plot | Verified against the final inclusion and exclusion reconciliation; retained for qualitative synthesis only and not represented in the final forest plots. |
| 68 | Huang WY 2008 | STI serology/history | Qualitative/contextual synthesis; not included in final plotted analyses. | Qualitative / not in final plot | Verified against the final inclusion and exclusion reconciliation; retained for qualitative synthesis only and not represented in the final forest plots. |
| 69 | Fernández L 2005 | Sexual behavior; STD history | Qualitative/contextual synthesis; not included in final plotted analyses. | Qualitative / not in final plot | Verified against the final inclusion and exclusion reconciliation; retained for qualitative synthesis only and not represented in the final forest plots. |
| 70 | La Vecchia C 1993 | Marital/sexual activity | Qualitative/contextual synthesis; not included in final plotted analyses. | Qualitative / not in final plot | Verified against the final inclusion and exclusion reconciliation; retained for qualitative synthesis only and not represented in the final forest plots. |
| 71 | Rosenblatt KA 2001 | Sexual factors | Qualitative/contextual synthesis; not included in final plotted analyses. | Qualitative / not in final plot | Verified against the final inclusion and exclusion reconciliation; retained for qualitative synthesis only and not represented in the final forest plots. |
| 72 | Lightfoot N 2004 | Medical/sexual/maturational factors | Qualitative/contextual synthesis; not included in final plotted analyses. | Qualitative / not in final plot | Verified against the final inclusion and exclusion reconciliation; retained for qualitative synthesis only and not represented in the final forest plots. |
| 73 | Spence AR 2014 | Sexual partners; STI history | Qualitative/contextual synthesis; not included in final plotted analyses. | Qualitative / not in final plot | Verified against the final inclusion and exclusion reconciliation; retained for qualitative synthesis only and not represented in the final forest plots. |
| 74 | Dubey B 2020 | STI exposure and host genetic interaction | Qualitative/contextual synthesis; not included in final plotted analyses. | Qualitative / not in final plot | Verified against the final inclusion and exclusion reconciliation; retained for qualitative synthesis only and not represented in the final forest plots. |
| 75 | Heshmat MY 1975 | Gonorrhea history | Qualitative/contextual synthesis; not included in final plotted analyses. | Qualitative / not in final plot | Verified against the final inclusion and exclusion reconciliation; retained for qualitative synthesis only and not represented in the final forest plots. |

Abbreviations: HPV, human papillomavirus; PCa, prostate cancer; STI, sexually transmitted infection. Reference numbers correspond to the revised manuscript reference list. Of 57 eligible primary observational reports, 25 unique reports enter at least one pooled model, 3 additional reports appear only in descriptive forest plots (28 plotted reports in total), and 29 are retained for qualitative or contextual synthesis.

**Supplementary Table S2. Report-level study design and risk-of-bias appraisal**

A structured domain-based appraisal informed by Newcastle-Ottawa Scale domains is reported for each of the 57 eligible primary observational reports. During revision, study-design labels, exposure timing, and comparator structure were rechecked against the source reports and locked review materials. No composite numeric NOS score is presented. Non-pooling is reported separately from risk of bias and is not interpreted as a quality grade. Dennis LK 2002 and Araujo-Neto AP 2016 were reclassified as excluded during final eligibility reconciliation; details are provided in Supplementary Table S6.

# S2A. Design, timing, comparator structure, and synthesis role

| **Ref. no.** | **Study/report** | **Evidence domain(s)** | **Use in synthesis** | **Source-verified design** | **Exposure timing** | **Comparator/reference group** |
| --- | --- | --- | --- | --- | --- | --- |
| 19 | McNicol PJ 1991 | HPV tissue/PCR detection | Qualitative/contextual synthesis only; not included in pooled or descriptive forest-plot analyses. | Comparative prostate-tissue study (PCa vs BPH; small normal-autopsy group also reported) | Viral markers measured in prostate tissue obtained at/around diagnostic or therapeutic tissue acquisition; exposure and case status are therefore contemporaneous for causal-temporality purposes. | BPH tissue; a small normal-autopsy tissue group was also reported. |
| 20 | Strickler HD 1998 | HPV tissue/PCR detection; HPV/viral marker serology | Entered ≥1 pooled model (some also contributed to sensitivity/subtype/descriptive analyses). | Hospital-based case-control comparative study with prostate tissue and serology | Viral markers measured in prostate tissue obtained at/around diagnostic or therapeutic tissue acquisition; exposure and case status are therefore contemporaneous for causal-temporality purposes. | Histologically confirmed BPH controls; tissue and serum comparisons. |
| 21 | Carozzi F 2004 | HPV tissue/PCR detection | Entered ≥1 pooled model (some also contributed to sensitivity/subtype/descriptive analyses). | Consecutive prostate-biopsy comparative study (malignant vs benign biopsies) | Viral markers measured in prostate tissue obtained at/around diagnostic or therapeutic tissue acquisition; exposure and case status are therefore contemporaneous for causal-temporality purposes. | Benign prostate core biopsies from the same consecutive biopsy series. |
| 22 | Leiros GJ 2005 | HPV tissue/PCR detection | Entered ≥1 pooled model (some also contributed to sensitivity/subtype/descriptive analyses). | Comparative prostate-tissue study (PCa vs prostatic hyperplasia) | Viral markers measured in prostate tissue obtained at/around diagnostic or therapeutic tissue acquisition; exposure and case status are therefore contemporaneous for causal-temporality purposes. | Prostatic hyperplasia controls. |
| 23 | Salehi Z 2012 | HPV tissue/PCR detection | Entered ≥1 pooled model (some also contributed to sensitivity/subtype/descriptive analyses). | Comparative prostate-tissue study (PCa vs BPH) | Viral markers measured in prostate tissue obtained at/around diagnostic or therapeutic tissue acquisition; exposure and case status are therefore contemporaneous for causal-temporality purposes. | BPH surgical/biopsy specimens. |
| 24 | Michopoulou V 2014 | HPV tissue/PCR detection | Entered ≥1 pooled model (some also contributed to sensitivity/subtype/descriptive analyses). | Comparative prostate-tissue study (PCa vs non-cancer/healthy prostate tissue) | Viral markers measured in prostate tissue obtained at/around diagnostic or therapeutic tissue acquisition; exposure and case status are therefore contemporaneous for causal-temporality purposes. | Non-cancer/healthy prostate tissue comparator used in the source extraction. |
| 25 | Singh N 2015 | HPV tissue/PCR detection; HPV16/18 tissue subtype | Entered ≥1 pooled model (some also contributed to sensitivity/subtype/descriptive analyses). | Case-control tissue study (PCa vs BPH) | Viral markers measured in prostate tissue obtained at/around diagnostic or therapeutic tissue acquisition; exposure and case status are therefore contemporaneous for causal-temporality purposes. | BPH controls. |
| 26 | Huang L 2016 | HPV tissue/PCR detection; HPV16/18 tissue subtype | Entered ≥1 pooled model (some also contributed to sensitivity/subtype/descriptive analyses). | Comparative prostate-tissue study (PCa vs BPH) | Viral markers measured in prostate tissue obtained at/around diagnostic or therapeutic tissue acquisition; exposure and case status are therefore contemporaneous for causal-temporality purposes. | BPH controls. |
| 27 | Babakhani F 2017 | HPV tissue/PCR detection | Entered ≥1 pooled model (some also contributed to sensitivity/subtype/descriptive analyses). | Comparative prostate-tissue study (PCa vs BPH) | Viral markers measured in prostate tissue obtained at/around diagnostic or therapeutic tissue acquisition; exposure and case status are therefore contemporaneous for causal-temporality purposes. | BPH controls. |
| 28 | Abdolmaleki N 2018 | HPV tissue/PCR detection | Entered ≥1 pooled model (some also contributed to sensitivity/subtype/descriptive analyses). | Comparative prostate-tissue study (PCa vs BPH) | Viral markers measured in prostate tissue obtained at/around diagnostic or therapeutic tissue acquisition; exposure and case status are therefore contemporaneous for causal-temporality purposes. | BPH controls. |
| 29 | Medel-Flores O 2018 | HPV tissue/PCR detection | Entered ≥1 pooled model (some also contributed to sensitivity/subtype/descriptive analyses). | Comparative prostate-tissue study (PCa vs BPH) | Viral markers measured in prostate tissue obtained at/around diagnostic or therapeutic tissue acquisition; exposure and case status are therefore contemporaneous for causal-temporality purposes. | BPH controls. |
| 30 | Ohadian Moghadam S 2021 | HPV tissue/PCR detection; HPV16/18 tissue subtype | Entered ≥1 pooled model (some also contributed to sensitivity/subtype/descriptive analyses). | Case-control tissue study (PCa vs BPH) | Viral markers measured in prostate tissue obtained at/around diagnostic or therapeutic tissue acquisition; exposure and case status are therefore contemporaneous for causal-temporality purposes. | BPH controls. |
| 31 | Ahmed MY 2023 | HPV tissue/PCR detection | Entered ≥1 pooled model (some also contributed to sensitivity/subtype/descriptive analyses). | Biopsy-based comparative tissue study (malignant vs benign prostate samples) | Viral markers measured in prostate tissue obtained at/around diagnostic or therapeutic tissue acquisition; exposure and case status are therefore contemporaneous for causal-temporality purposes. | Benign prostate samples from the UK biopsy/tissue series. |
| 32 | Todorova E 2025 | HPV/EBV tissue detection | Entered ≥1 pooled model (some also contributed to sensitivity/subtype/descriptive analyses). | Comparative prostate-tissue study (PCa vs BPH) | Viral markers measured in prostate tissue obtained at/around diagnostic or therapeutic tissue acquisition; exposure and case status are therefore contemporaneous for causal-temporality purposes. | BPH controls. |
| 33 | Rodríguez-Romero BI 2025 | HPV tissue/PCR detection; HPV16/18 tissue subtype | Entered ≥1 pooled model (some also contributed to sensitivity/subtype/descriptive analyses). | Comparative prostate-tissue study (PCa vs BPH) | Viral markers measured in prostate tissue obtained at/around diagnostic or therapeutic tissue acquisition; exposure and case status are therefore contemporaneous for causal-temporality purposes. | BPH controls. |
| 34 | Dillner J 1998 | HPV serology | Entered ≥1 pooled model (some also contributed to sensitivity/subtype/descriptive analyses). | Nested case-control study within a prospective serum bank | Serum/plasma obtained before prostate-cancer diagnosis or case ascertainment within a defined parent cohort/biobank. | Controls selected from the same source cohort and cancer-free/at risk at the relevant case time, according to the source report. |
| 35 | Hisada M 2000 | HPV16 serology | Entered ≥1 pooled model (some also contributed to sensitivity/subtype/descriptive analyses). | Nested case-control serology study (brief report) | Archived serum used in a nested case-control analysis; the brief JAMA report provides limited methodological detail in accessible text. | Controls from the same source population; exact sampling details are not fully reported in the accessible brief report. |
| 36 | Rosenblatt KA 2003 | HPV16/18 serology; sexual factors | Entered ≥1 pooled model (some also contributed to sensitivity/subtype/descriptive analyses). | Population-based case-control study | Serology measured after case/control selection; antibodies reflect prior infection but sampling itself was not prospectively anchored before diagnosis. | Population controls from King County, Washington. |
| 37 | Korodi Z 2005 | HPV16/18 serology | Entered ≥1 pooled model (some also contributed to sensitivity/subtype/descriptive analyses). | Nordic nested case-control study within prospective biobank cohorts | Serum/plasma obtained before prostate-cancer diagnosis or case ascertainment within a defined parent cohort/biobank. | Controls selected from the same source cohort and cancer-free/at risk at the relevant case time, according to the source report. |
| 38 | Sutcliffe S 2010 | HPV16/18 serology | Entered ≥1 pooled model (some also contributed to sensitivity/subtype/descriptive analyses). | Nested case-control study within the Prostate Cancer Prevention Trial | Serum/plasma obtained before prostate-cancer diagnosis or case ascertainment within a defined parent cohort/biobank. | Controls selected from the same source cohort and cancer-free/at risk at the relevant case time, according to the source report. |
| 39 | Sutcliffe S 2006 | T. vaginalis serology; STI history | Entered ≥1 pooled model (some also contributed to sensitivity/subtype/descriptive analyses). | Nested case-control study within the Health Professionals Follow-up Study | Serum/plasma obtained before prostate-cancer diagnosis or case ascertainment within a defined parent cohort/biobank. | Controls selected from the same source cohort and cancer-free/at risk at the relevant case time, according to the source report. |
| 40 | Stark JR 2009 | T. vaginalis serology | Qualitative/contextual synthesis only; not included in pooled or descriptive forest-plot analyses. | Nested case-control study within the Physicians' Health Study | Serum/plasma obtained before prostate-cancer diagnosis or case ascertainment within a defined parent cohort/biobank. | Controls selected from the same source cohort and cancer-free/at risk at the relevant case time, according to the source report. |
| 41 | Fowke JH 2016 | T. vaginalis serology | Qualitative/contextual synthesis only; not included in pooled or descriptive forest-plot analyses. | Nested case-control study within the Southern Community Cohort Study | Serum/plasma obtained before prostate-cancer diagnosis or case ascertainment within a defined parent cohort/biobank. | Controls selected from the same source cohort and cancer-free/at risk at the relevant case time, according to the source report. |
| 42 | Shui IM 2016 | T. vaginalis serology; aggressive/advanced PCa | Entered ≥1 pooled model (some also contributed to sensitivity/subtype/descriptive analyses). | Case-control serology analysis using participants from two prior population-based case-control studies | Serology measured after case/control selection; antibodies reflect prior infection but sampling itself was not prospectively anchored before diagnosis. | Age-matched controls drawn from two prior population-based case-control studies. |
| 43 | Marous M 2017 | T. vaginalis serology | Entered ≥1 pooled model (some also contributed to sensitivity/subtype/descriptive analyses). | Nested case-control study within the PLCO Cancer Screening Trial | Serum/plasma obtained before prostate-cancer diagnosis or case ascertainment within a defined parent cohort/biobank. | Controls selected from the same source cohort and cancer-free/at risk at the relevant case time, according to the source report. |
| 44 | Saleh NE 2021 | T. vaginalis serology | Entered ≥1 pooled model (some also contributed to sensitivity/subtype/descriptive analyses). | Case-control serology study | Serology measured after case/control selection; antibodies reflect prior infection but sampling itself was not prospectively anchored before diagnosis. | Age-matched non-PCa comparison groups including healthy controls; the report also included other-cancer groups. |
| 45 | Sawaya M 2024 | History-based STI; specific STI; other infection markers | Entered ≥1 pooled model (some also contributed to sensitivity/subtype/descriptive analyses). | Population-based case-control study (EPICAP) | Exposure was assessed after case/control identification or retrospectively for pre-diagnostic history; temporal ordering therefore depends on recalled/recorded history rather than prospective measurement. | Population controls from the same French geographic source population. |
| 46 | Ángeles-Garay U 2019 | History-based STI; sexual behavior | Entered ≥1 pooled model (some also contributed to sensitivity/subtype/descriptive analyses). | Matched case-control study | Exposure was assessed after case/control identification or retrospectively for pre-diagnostic history; temporal ordering therefore depends on recalled/recorded history rather than prospective measurement. | Age-matched controls without prostate-cancer diagnosis. |
| 47 | Mandel JS 1987 | Sexual behavior | Descriptive forest plot only; not included in a pooled model. | Case-control study | Exposure was assessed after case/control identification or retrospectively for pre-diagnostic history; temporal ordering therefore depends on recalled/recorded history rather than prospective measurement. | Controls from the same case-control study base. |
| 48 | Honda GD 1988 | Sexual behavior | Descriptive forest plot only; not included in a pooled model. | Population-based matched case-control study | Exposure was assessed after case/control identification or retrospectively for pre-diagnostic history; temporal ordering therefore depends on recalled/recorded history rather than prospective measurement. | Population controls individually matched to cases. |
| 49 | Hayes RB 2000 | Sexual behavior; STI history | Qualitative/contextual synthesis only; not included in pooled or descriptive forest-plot analyses. | Population-based case-control study | Exposure was assessed after case/control identification or retrospectively for pre-diagnostic history; temporal ordering therefore depends on recalled/recorded history rather than prospective measurement. | Population controls; Black and White men were included. |
| 50 | Sarma AV 2006 | Sexual behavior; STI history; prostatitis | Qualitative/contextual synthesis only; not included in pooled or descriptive forest-plot analyses. | Population-based case-control study | Exposure was assessed after case/control identification or retrospectively for pre-diagnostic history; temporal ordering therefore depends on recalled/recorded history rather than prospective measurement. | Population-based non-PCa controls from the same geographic study base. |
| 51 | Hennis AJM 2013 | Sexual behavior; urologic characteristics | Qualitative/contextual synthesis only; not included in pooled or descriptive forest-plot analyses. | National/population-based case-control study | Exposure was assessed after case/control identification or retrospectively for pre-diagnostic history; temporal ordering therefore depends on recalled/recorded history rather than prospective measurement. | Controls from the Barbados national case-control study. |
| 52 | Leitzmann MF 2004 | Ejaculation frequency | Qualitative/contextual synthesis only; not included in pooled or descriptive forest-plot analyses. | Prospective cohort study (Health Professionals Follow-up Study) | Ejaculation-frequency history reported on the 1992 questionnaire before subsequent prostate-cancer follow-up. | Lower/higher ejaculation-frequency categories within the HPFS cohort. |
| 53 | Rider JR 2016 | Ejaculation frequency | Qualitative/contextual synthesis only; not included in pooled or descriptive forest-plot analyses. | Prospective cohort study (Health Professionals Follow-up Study; extended follow-up) | Ejaculation-frequency history reported before outcome follow-up; extended follow-up of the HPFS analysis through 2010. | Lower/higher ejaculation-frequency categories within the HPFS cohort. |
| 54 | Papa NP 2017 | Ejaculation frequency; aggressive PCa | Qualitative/contextual synthesis only; not included in pooled or descriptive forest-plot analyses. | Case-control study | Exposure was assessed after case/control identification or retrospectively for pre-diagnostic history; temporal ordering therefore depends on recalled/recorded history rather than prospective measurement. | Male controls sampled from the same Victorian urology-practice study base. |
| 55 | Nair-Shalliker V 2017 | Sexual history; adolescent sexual development | Qualitative/contextual synthesis only; not included in pooled or descriptive forest-plot analyses. | Case-control study (NSW CLEAR) | Exposure was assessed after case/control identification or retrospectively for pre-diagnostic history; temporal ordering therefore depends on recalled/recorded history rather than prospective measurement. | Male controls from the NSW CLEAR study. |
| 56 | Lozano-Lorca M 2023 | Ejaculation frequency | Qualitative/contextual synthesis only; not included in pooled or descriptive forest-plot analyses. | Population-based case-control study (CAPLIFE) | Exposure was assessed after case/control identification or retrospectively for pre-diagnostic history; temporal ordering therefore depends on recalled/recorded history rather than prospective measurement. | Population controls from the CAPLIFE study. |
| 57 | McDonald AC 2011 | HHV-8 serology | Descriptive forest plot only; not included in a pooled model. | Case-cohort study | Baseline HHV-8 serostatus measured before subsequent screen-detected incident prostate cancer. | Subcohort comparison sampled from the Tobago study cohort according to the case-cohort design. |
| 58 | Yin SH 2024 | Claims-defined HPV infection | Qualitative/contextual synthesis only; not included in pooled or descriptive forest-plot analyses. | Claims-based propensity-score-matched case-control study | Administrative HPV diagnoses recorded before the prostate-cancer index date. | Propensity-score-matched men without prostate cancer in the same insurance database. |
| 59 | Aghakhani A 2011 | HPV tissue detection | Qualitative/contextual synthesis only; not included in pooled or descriptive forest-plot analyses. | Comparative prostate-tissue study (PCa vs BPH) | Viral markers measured in prostate tissue obtained at/around diagnostic or therapeutic tissue acquisition; exposure and case status are therefore contemporaneous for causal-temporality purposes. | BPH controls. |
| 60 | Whitaker NJ 2013 | HPV/EBV tissue detection | Qualitative/contextual synthesis only; not included in pooled or descriptive forest-plot analyses. | Comparative tissue study (normal, benign, and malignant prostate tissue) | Viral markers measured in prostate tissue obtained at/around diagnostic or therapeutic tissue acquisition; exposure and case status are therefore contemporaneous for causal-temporality purposes. | Normal and benign prostate tissues. |
| 61 | Cheng I 2010 | STI history; prostatitis | Qualitative/contextual synthesis only; not included in pooled or descriptive forest-plot analyses. | Prospective cohort study (California Men's Health Study) | STD/prostatitis history recorded in the cohort before subsequent prostate-cancer ascertainment. | Men without the corresponding exposure/history within the California Men's Health Study. |
| 62 | Chung SD 2013 | Claims-defined STI history | Qualitative/contextual synthesis only; not included in pooled or descriptive forest-plot analyses. | Population-based matched retrospective cohort study | STI diagnosis defined from administrative claims before subsequent 5-year prostate-cancer follow-up. | Matched men without the indexed STI diagnosis. |
| 63 | Dennis LK 2009 | STI serology | Qualitative/contextual synthesis only; not included in pooled or descriptive forest-plot analyses. | Nested case-control study within a U.S. military cohort | Serum/plasma obtained before prostate-cancer diagnosis or case ascertainment within a defined parent cohort/biobank. | Controls selected from the same source cohort and cancer-free/at risk at the relevant case time, according to the source report. |
| 64 | Wang YC 2017 | Claims-defined gonorrhea | Qualitative/contextual synthesis only; not included in pooled or descriptive forest-plot analyses. | Nationwide population-based retrospective cohort study | Gonorrhea diagnosis defined from administrative claims before subsequent prostate-cancer follow-up. | Age/index-year matched men without gonorrhea. |
| 65 | Vázquez-Salas RA 2016 | Gonorrhea/STI history; sexual behavior | Qualitative/contextual synthesis only; not included in pooled or descriptive forest-plot analyses. | Population-based case-control study | Exposure was assessed after case/control identification or retrospectively for pre-diagnostic history; temporal ordering therefore depends on recalled/recorded history rather than prospective measurement. | Age-matched population controls without prostate cancer. |
| 66 | Anttila T 2005 | Chlamydia serology | Qualitative/contextual synthesis only; not included in pooled or descriptive forest-plot analyses. | Nested case-control study within Nordic serum-bank cohorts | Serum/plasma obtained before prostate-cancer diagnosis or case ascertainment within a defined parent cohort/biobank. | Controls selected from the same source cohort and cancer-free/at risk at the relevant case time, according to the source report. |
| 67 | Sutcliffe S 2007 | Chlamydia/HPV/HHV-8 serology | Qualitative/contextual synthesis only; not included in pooled or descriptive forest-plot analyses. | Nested case-control study within the Health Professionals Follow-up Study | Serum/plasma obtained before prostate-cancer diagnosis or case ascertainment within a defined parent cohort/biobank. | Controls selected from the same source cohort and cancer-free/at risk at the relevant case time, according to the source report. |
| 68 | Huang WY 2008 | STI serology/history | Qualitative/contextual synthesis only; not included in pooled or descriptive forest-plot analyses. | Nested case-control study within the PLCO Cancer Screening Trial | Serum/plasma obtained before prostate-cancer diagnosis or case ascertainment within a defined parent cohort/biobank. | Controls selected from the same source cohort and cancer-free/at risk at the relevant case time, according to the source report. |
| 69 | Fernández L 2005 | Sexual behavior; STD history | Qualitative/contextual synthesis only; not included in pooled or descriptive forest-plot analyses. | Case-control study | Exposure was assessed after case/control identification or retrospectively for pre-diagnostic history; temporal ordering therefore depends on recalled/recorded history rather than prospective measurement. | Controls from the Cuban case-control study. |
| 70 | La Vecchia C 1993 | Marital/sexual activity | Qualitative/contextual synthesis only; not included in pooled or descriptive forest-plot analyses. | Hospital-based case-control study | Exposure was assessed after case/control identification or retrospectively for pre-diagnostic history; temporal ordering therefore depends on recalled/recorded history rather than prospective measurement. | Hospital controls admitted for acute non-neoplastic, non-genital/urologic conditions. |
| 71 | Rosenblatt KA 2001 | Sexual factors | Qualitative/contextual synthesis only; not included in pooled or descriptive forest-plot analyses. | Population-based case-control study | Exposure was assessed after case/control identification or retrospectively for pre-diagnostic history; temporal ordering therefore depends on recalled/recorded history rather than prospective measurement. | Population controls identified by random-digit dialing and frequency matched on age. |
| 72 | Lightfoot N 2004 | Medical/sexual/maturational factors | Qualitative/contextual synthesis only; not included in pooled or descriptive forest-plot analyses. | Population-based case-control study | Exposure was assessed after case/control identification or retrospectively for pre-diagnostic history; temporal ordering therefore depends on recalled/recorded history rather than prospective measurement. | Population controls from telephone listings, age-frequency matched. |
| 73 | Spence AR 2014 | Sexual partners; STI history | Qualitative/contextual synthesis only; not included in pooled or descriptive forest-plot analyses. | Population-based case-control study (PROtEuS) | Exposure was assessed after case/control identification or retrospectively for pre-diagnostic history; temporal ordering therefore depends on recalled/recorded history rather than prospective measurement. | Population controls from the PROtEuS source population. |
| 74 | Dubey B 2020 | STI exposure and host genetic interaction | Qualitative/contextual synthesis only; not included in pooled or descriptive forest-plot analyses. | Case-control study | Exposure was assessed after case/control identification or retrospectively for pre-diagnostic history; temporal ordering therefore depends on recalled/recorded history rather than prospective measurement. | Non-PCa controls from the Jamaican case-control study. |
| 75 | Heshmat MY 1975 | Gonorrhea history | Qualitative/contextual synthesis only; not included in pooled or descriptive forest-plot analyses. | Retrospective age-matched case-control study | Exposure was assessed after case/control identification or retrospectively for pre-diagnostic history; temporal ordering therefore depends on recalled/recorded history rather than prospective measurement. | Age-matched controls in the U.S. retrospective case-control component. |

# S2B. Verified risk-of-bias considerations and interpretation

| **Ref. no.** | **Study/report** | **Verified main risk-of-bias considerations** | **Interpretation for synthesis** |
| --- | --- | --- | --- |
| 19 | McNicol PJ 1991 | Comparator and tissue-source selection; clinical indication for tissue acquisition; contemporaneous viral-marker measurement with established disease status; assay definition/sensitivity; contamination control and laboratory blinding where reported; residual confounding. | Eligible comparative evidence. Tissue/PCR findings can support an observed case/non-cancer association, but cannot by themselves establish that viral exposure preceded carcinogenesis. |
| 20 | Strickler HD 1998 | Comparator and tissue-source selection; clinical indication for tissue acquisition; contemporaneous viral-marker measurement with established disease status; assay definition/sensitivity; contamination control and laboratory blinding where reported; residual confounding. | Eligible comparative evidence. Tissue/PCR findings can support an observed case/non-cancer association, but cannot by themselves establish that viral exposure preceded carcinogenesis. |
| 21 | Carozzi F 2004 | Comparator and tissue-source selection; clinical indication for tissue acquisition; contemporaneous viral-marker measurement with established disease status; assay definition/sensitivity; contamination control and laboratory blinding where reported; residual confounding. | Eligible comparative evidence. Tissue/PCR findings can support an observed case/non-cancer association, but cannot by themselves establish that viral exposure preceded carcinogenesis. |
| 22 | Leiros GJ 2005 | Comparator and tissue-source selection; clinical indication for tissue acquisition; contemporaneous viral-marker measurement with established disease status; assay definition/sensitivity; contamination control and laboratory blinding where reported; residual confounding. | Eligible comparative evidence. Tissue/PCR findings can support an observed case/non-cancer association, but cannot by themselves establish that viral exposure preceded carcinogenesis. |
| 23 | Salehi Z 2012 | Comparator and tissue-source selection; clinical indication for tissue acquisition; contemporaneous viral-marker measurement with established disease status; assay definition/sensitivity; contamination control and laboratory blinding where reported; residual confounding. | Eligible comparative evidence. Tissue/PCR findings can support an observed case/non-cancer association, but cannot by themselves establish that viral exposure preceded carcinogenesis. |
| 24 | Michopoulou V 2014 | Comparator and tissue-source selection; clinical indication for tissue acquisition; contemporaneous viral-marker measurement with established disease status; assay definition/sensitivity; contamination control and laboratory blinding where reported; residual confounding. | Eligible comparative evidence. Tissue/PCR findings can support an observed case/non-cancer association, but cannot by themselves establish that viral exposure preceded carcinogenesis. |
| 25 | Singh N 2015 | Comparator and tissue-source selection; clinical indication for tissue acquisition; contemporaneous viral-marker measurement with established disease status; assay definition/sensitivity; contamination control and laboratory blinding where reported; residual confounding. | Eligible comparative evidence. Tissue/PCR findings can support an observed case/non-cancer association, but cannot by themselves establish that viral exposure preceded carcinogenesis. |
| 26 | Huang L 2016 | Comparator and tissue-source selection; clinical indication for tissue acquisition; contemporaneous viral-marker measurement with established disease status; assay definition/sensitivity; contamination control and laboratory blinding where reported; residual confounding. | Eligible comparative evidence. Tissue/PCR findings can support an observed case/non-cancer association, but cannot by themselves establish that viral exposure preceded carcinogenesis. |
| 27 | Babakhani F 2017 | Comparator and tissue-source selection; clinical indication for tissue acquisition; contemporaneous viral-marker measurement with established disease status; assay definition/sensitivity; contamination control and laboratory blinding where reported; residual confounding. | Eligible comparative evidence. Tissue/PCR findings can support an observed case/non-cancer association, but cannot by themselves establish that viral exposure preceded carcinogenesis. |
| 28 | Abdolmaleki N 2018 | Comparator and tissue-source selection; clinical indication for tissue acquisition; contemporaneous viral-marker measurement with established disease status; assay definition/sensitivity; contamination control and laboratory blinding where reported; residual confounding. | Eligible comparative evidence. Tissue/PCR findings can support an observed case/non-cancer association, but cannot by themselves establish that viral exposure preceded carcinogenesis. |
| 29 | Medel-Flores O 2018 | Comparator and tissue-source selection; clinical indication for tissue acquisition; contemporaneous viral-marker measurement with established disease status; assay definition/sensitivity; contamination control and laboratory blinding where reported; residual confounding. | Eligible comparative evidence. Tissue/PCR findings can support an observed case/non-cancer association, but cannot by themselves establish that viral exposure preceded carcinogenesis. |
| 30 | Ohadian Moghadam S 2021 | Comparator and tissue-source selection; clinical indication for tissue acquisition; contemporaneous viral-marker measurement with established disease status; assay definition/sensitivity; contamination control and laboratory blinding where reported; residual confounding. | Eligible comparative evidence. Tissue/PCR findings can support an observed case/non-cancer association, but cannot by themselves establish that viral exposure preceded carcinogenesis. |
| 31 | Ahmed MY 2023 | Comparator and tissue-source selection; clinical indication for tissue acquisition; contemporaneous viral-marker measurement with established disease status; assay definition/sensitivity; contamination control and laboratory blinding where reported; residual confounding. | Eligible comparative evidence. Tissue/PCR findings can support an observed case/non-cancer association, but cannot by themselves establish that viral exposure preceded carcinogenesis. |
| 32 | Todorova E 2025 | Comparator and tissue-source selection; clinical indication for tissue acquisition; contemporaneous viral-marker measurement with established disease status; assay definition/sensitivity; contamination control and laboratory blinding where reported; residual confounding. | Eligible comparative evidence. Tissue/PCR findings can support an observed case/non-cancer association, but cannot by themselves establish that viral exposure preceded carcinogenesis. |
| 33 | Rodríguez-Romero BI 2025 | Comparator and tissue-source selection; clinical indication for tissue acquisition; contemporaneous viral-marker measurement with established disease status; assay definition/sensitivity; contamination control and laboratory blinding where reported; residual confounding. | Eligible comparative evidence. Tissue/PCR findings can support an observed case/non-cancer association, but cannot by themselves establish that viral exposure preceded carcinogenesis. |
| 34 | Dillner J 1998 | Parent-cohort/biobank selection; control sampling from the risk set; serologic assay thresholds and reproducibility; screening/outcome ascertainment where relevant; residual confounding. | Prediagnostic biomarker evidence with stronger temporal ordering than contemporaneous tissue studies; remaining concerns relate to assay classification, cohort selection, screening, and confounding. |
| 35 | Hisada M 2000 | Brief-report methodological detail is limited in accessible text; key concerns are source-population/control sampling, serologic assay classification, and residual confounding. | Nested case-control design is supported by a contemporaneous peer-reviewed comparative table; the original brief report provides limited accessible methodological detail, so no finer RoB claims are made. |
| 36 | Rosenblatt KA 2003 | Case/control selection; serologic assay thresholds/reproducibility; blood sampling after case/control ascertainment; screening/detection differences; residual confounding. | Eligible case-control serology evidence. Antibodies indicate prior exposure, but non-prediagnostic sampling does not provide the same temporal protection as nested prospective serology. |
| 37 | Korodi Z 2005 | Parent-cohort/biobank selection; control sampling from the risk set; serologic assay thresholds and reproducibility; screening/outcome ascertainment where relevant; residual confounding. | Prediagnostic biomarker evidence with stronger temporal ordering than contemporaneous tissue studies; remaining concerns relate to assay classification, cohort selection, screening, and confounding. |
| 38 | Sutcliffe S 2010 | Parent-cohort/biobank selection; control sampling from the risk set; serologic assay thresholds and reproducibility; screening/outcome ascertainment where relevant; residual confounding. | Prediagnostic biomarker evidence with stronger temporal ordering than contemporaneous tissue studies; remaining concerns relate to assay classification, cohort selection, screening, and confounding. |
| 39 | Sutcliffe S 2006 | Parent-cohort/biobank selection; control sampling from the risk set; serologic assay thresholds and reproducibility; screening/outcome ascertainment where relevant; residual confounding. | Prediagnostic biomarker evidence with stronger temporal ordering than contemporaneous tissue studies; remaining concerns relate to assay classification, cohort selection, screening, and confounding. |
| 40 | Stark JR 2009 | Parent-cohort/biobank selection; control sampling from the risk set; serologic assay thresholds and reproducibility; screening/outcome ascertainment where relevant; residual confounding. | Prediagnostic biomarker evidence with stronger temporal ordering than contemporaneous tissue studies; remaining concerns relate to assay classification, cohort selection, screening, and confounding. |
| 41 | Fowke JH 2016 | Parent-cohort/biobank selection; control sampling from the risk set; serologic assay thresholds and reproducibility; screening/outcome ascertainment where relevant; residual confounding. | Prediagnostic biomarker evidence with stronger temporal ordering than contemporaneous tissue studies; remaining concerns relate to assay classification, cohort selection, screening, and confounding. |
| 42 | Shui IM 2016 | Case/control selection; serologic assay thresholds/reproducibility; blood sampling after case/control ascertainment; screening/detection differences; residual confounding. | Eligible case-control serology evidence. Antibodies indicate prior exposure, but non-prediagnostic sampling does not provide the same temporal protection as nested prospective serology. |
| 43 | Marous M 2017 | Parent-cohort/biobank selection; control sampling from the risk set; serologic assay thresholds and reproducibility; screening/outcome ascertainment where relevant; residual confounding. | Prediagnostic biomarker evidence with stronger temporal ordering than contemporaneous tissue studies; remaining concerns relate to assay classification, cohort selection, screening, and confounding. |
| 44 | Saleh NE 2021 | Case/control selection; serologic assay thresholds/reproducibility; blood sampling after case/control ascertainment; screening/detection differences; residual confounding. | Eligible case-control serology evidence. Antibodies indicate prior exposure, but non-prediagnostic sampling does not provide the same temporal protection as nested prospective serology. |
| 45 | Sawaya M 2024 | Case/control selection; retrospective exposure ascertainment; recall and/or social-desirability bias where self-report was used; PSA/healthcare-related detection differences; residual confounding. | Eligible comparative evidence; retrospective exposure ascertainment limits causal-temporal inference. Non-pooling, where applicable, reflects exposure/estimate incompatibility rather than a RoB grade. |
| 46 | Ángeles-Garay U 2019 | Case/control selection; retrospective exposure ascertainment; recall and/or social-desirability bias where self-report was used; PSA/healthcare-related detection differences; residual confounding. | Eligible comparative evidence; retrospective exposure ascertainment limits causal-temporal inference. Non-pooling, where applicable, reflects exposure/estimate incompatibility rather than a RoB grade. |
| 47 | Mandel JS 1987 | Case/control selection; retrospective exposure ascertainment; recall and/or social-desirability bias where self-report was used; PSA/healthcare-related detection differences; residual confounding. | Eligible comparative evidence; retrospective exposure ascertainment limits causal-temporal inference. Non-pooling, where applicable, reflects exposure/estimate incompatibility rather than a RoB grade. |
| 48 | Honda GD 1988 | Case/control selection; retrospective exposure ascertainment; recall and/or social-desirability bias where self-report was used; PSA/healthcare-related detection differences; residual confounding. | Eligible comparative evidence; retrospective exposure ascertainment limits causal-temporal inference. Non-pooling, where applicable, reflects exposure/estimate incompatibility rather than a RoB grade. |
| 49 | Hayes RB 2000 | Case/control selection; retrospective exposure ascertainment; recall and/or social-desirability bias where self-report was used; PSA/healthcare-related detection differences; residual confounding. | Eligible comparative evidence; retrospective exposure ascertainment limits causal-temporal inference. Non-pooling, where applicable, reflects exposure/estimate incompatibility rather than a RoB grade. |
| 50 | Sarma AV 2006 | Case/control selection; retrospective exposure ascertainment; recall and/or social-desirability bias where self-report was used; PSA/healthcare-related detection differences; residual confounding. | Eligible comparative evidence; retrospective exposure ascertainment limits causal-temporal inference. Non-pooling, where applicable, reflects exposure/estimate incompatibility rather than a RoB grade. |
| 51 | Hennis AJM 2013 | Case/control selection; retrospective exposure ascertainment; recall and/or social-desirability bias where self-report was used; PSA/healthcare-related detection differences; residual confounding. | Eligible comparative evidence; retrospective exposure ascertainment limits causal-temporal inference. Non-pooling, where applicable, reflects exposure/estimate incompatibility rather than a RoB grade. |
| 52 | Leitzmann MF 2004 | Exposure misclassification; changes in exposure over time; outcome ascertainment; PSA/surveillance differences; loss to follow-up where applicable; residual confounding. | Longitudinal evidence with exposure preceding incident prostate-cancer ascertainment; interpretation remains observational and susceptible to screening and residual confounding. This report shares the HPFS parent cohort with the later Rider 2016 update. |
| 53 | Rider JR 2016 | Exposure misclassification; changes in exposure over time; outcome ascertainment; PSA/surveillance differences; loss to follow-up where applicable; residual confounding. | Longitudinal evidence with exposure preceding incident prostate-cancer ascertainment; interpretation remains observational and susceptible to screening and residual confounding. This report is an extended follow-up of the same HPFS exposure analysis reported earlier by Leitzmann 2004. |
| 54 | Papa NP 2017 | Case/control selection; retrospective exposure ascertainment; recall and/or social-desirability bias where self-report was used; PSA/healthcare-related detection differences; residual confounding. | Eligible comparative evidence; retrospective exposure ascertainment limits causal-temporal inference. Non-pooling, where applicable, reflects exposure/estimate incompatibility rather than a RoB grade. |
| 55 | Nair-Shalliker V 2017 | Case/control selection; retrospective exposure ascertainment; recall and/or social-desirability bias where self-report was used; PSA/healthcare-related detection differences; residual confounding. | Eligible comparative evidence; retrospective exposure ascertainment limits causal-temporal inference. Non-pooling, where applicable, reflects exposure/estimate incompatibility rather than a RoB grade. |
| 56 | Lozano-Lorca M 2023 | Case/control selection; retrospective exposure ascertainment; recall and/or social-desirability bias where self-report was used; PSA/healthcare-related detection differences; residual confounding. | Eligible comparative evidence; retrospective exposure ascertainment limits causal-temporal inference. Non-pooling, where applicable, reflects exposure/estimate incompatibility rather than a RoB grade. |
| 57 | McDonald AC 2011 | Subcohort sampling/representativeness; handling of the case-cohort sampling structure; baseline HHV-8 assay classification; screen-detected outcome ascertainment; residual confounding. | Prediagnostic case-cohort evidence; not equivalent to a nested case-control design and should be appraised using its sampling structure. |
| 58 | Yin SH 2024 | Administrative coding validity for HPV and prostate cancer; differential healthcare contact/surveillance; residual confounding despite propensity-score matching; inability to verify infection biology from claims. | Eligible claims-based case-control evidence; interpreted as diagnosis-history association rather than direct viral detection. |
| 59 | Aghakhani A 2011 | Comparator and tissue-source selection; clinical indication for tissue acquisition; contemporaneous viral-marker measurement with established disease status; assay definition/sensitivity; contamination control and laboratory blinding where reported; residual confounding. | Eligible comparative evidence. Tissue/PCR findings can support an observed case/non-cancer association, but cannot by themselves establish that viral exposure preceded carcinogenesis. |
| 60 | Whitaker NJ 2013 | Comparator and tissue-source selection; clinical indication for tissue acquisition; contemporaneous viral-marker measurement with established disease status; assay definition/sensitivity; contamination control and laboratory blinding where reported; residual confounding. | Eligible comparative evidence. Tissue/PCR findings can support an observed case/non-cancer association, but cannot by themselves establish that viral exposure preceded carcinogenesis. |
| 61 | Cheng I 2010 | Exposure misclassification; changes in exposure over time; outcome ascertainment; PSA/surveillance differences; loss to follow-up where applicable; residual confounding. | Longitudinal evidence with exposure preceding incident prostate-cancer ascertainment; interpretation remains observational and susceptible to screening and residual confounding. |
| 62 | Chung SD 2013 | Administrative STI coding; exposure misclassification; differential healthcare surveillance; outcome coding; residual confounding. | Longitudinal evidence with exposure preceding incident prostate-cancer ascertainment; interpretation remains observational and susceptible to screening and residual confounding. |
| 63 | Dennis LK 2009 | Parent-cohort/biobank selection; control sampling from the risk set; serologic assay thresholds and reproducibility; screening/outcome ascertainment where relevant; residual confounding. | Prediagnostic biomarker evidence with stronger temporal ordering than contemporaneous tissue studies; remaining concerns relate to assay classification, cohort selection, screening, and confounding. |
| 64 | Wang YC 2017 | Administrative gonorrhea coding; exposure misclassification; differential healthcare surveillance; outcome coding; residual confounding. | Longitudinal evidence with exposure preceding incident prostate-cancer ascertainment; interpretation remains observational and susceptible to screening and residual confounding. |
| 65 | Vázquez-Salas RA 2016 | Case/control selection; retrospective exposure ascertainment; recall and/or social-desirability bias where self-report was used; PSA/healthcare-related detection differences; residual confounding. | Eligible comparative evidence; retrospective exposure ascertainment limits causal-temporal inference. Non-pooling, where applicable, reflects exposure/estimate incompatibility rather than a RoB grade. |
| 66 | Anttila T 2005 | Parent-cohort/biobank selection; control sampling from the risk set; serologic assay thresholds and reproducibility; screening/outcome ascertainment where relevant; residual confounding. | Prediagnostic biomarker evidence with stronger temporal ordering than contemporaneous tissue studies; remaining concerns relate to assay classification, cohort selection, screening, and confounding. |
| 67 | Sutcliffe S 2007 | Parent-cohort/biobank selection; control sampling from the risk set; serologic assay thresholds and reproducibility; screening/outcome ascertainment where relevant; residual confounding. | Prediagnostic biomarker evidence with stronger temporal ordering than contemporaneous tissue studies; remaining concerns relate to assay classification, cohort selection, screening, and confounding. |
| 68 | Huang WY 2008 | Parent-cohort/biobank selection; control sampling from the risk set; serologic assay thresholds and reproducibility; screening/outcome ascertainment where relevant; residual confounding. | Prediagnostic biomarker evidence with stronger temporal ordering than contemporaneous tissue studies; remaining concerns relate to assay classification, cohort selection, screening, and confounding. |
| 69 | Fernández L 2005 | Case/control selection; retrospective exposure ascertainment; recall and/or social-desirability bias where self-report was used; PSA/healthcare-related detection differences; residual confounding. | Eligible comparative evidence; retrospective exposure ascertainment limits causal-temporal inference. Non-pooling, where applicable, reflects exposure/estimate incompatibility rather than a RoB grade. |
| 70 | La Vecchia C 1993 | Case/control selection; retrospective exposure ascertainment; recall and/or social-desirability bias where self-report was used; PSA/healthcare-related detection differences; residual confounding. | Eligible comparative evidence; retrospective exposure ascertainment limits causal-temporal inference. Non-pooling, where applicable, reflects exposure/estimate incompatibility rather than a RoB grade. |
| 71 | Rosenblatt KA 2001 | Case/control selection; retrospective exposure ascertainment; recall and/or social-desirability bias where self-report was used; PSA/healthcare-related detection differences; residual confounding. | Eligible comparative evidence; retrospective exposure ascertainment limits causal-temporal inference. Non-pooling, where applicable, reflects exposure/estimate incompatibility rather than a RoB grade. |
| 72 | Lightfoot N 2004 | Case/control selection; retrospective exposure ascertainment; recall and/or social-desirability bias where self-report was used; PSA/healthcare-related detection differences; residual confounding. | Eligible comparative evidence; retrospective exposure ascertainment limits causal-temporal inference. Non-pooling, where applicable, reflects exposure/estimate incompatibility rather than a RoB grade. |
| 73 | Spence AR 2014 | Case/control selection; retrospective exposure ascertainment; recall and/or social-desirability bias where self-report was used; PSA/healthcare-related detection differences; residual confounding. | Eligible comparative evidence; retrospective exposure ascertainment limits causal-temporal inference. Non-pooling, where applicable, reflects exposure/estimate incompatibility rather than a RoB grade. |
| 74 | Dubey B 2020 | Case/control selection; retrospective exposure ascertainment; recall and/or social-desirability bias where self-report was used; PSA/healthcare-related detection differences; residual confounding. | Eligible comparative evidence; retrospective exposure ascertainment limits causal-temporal inference. Non-pooling, where applicable, reflects exposure/estimate incompatibility rather than a RoB grade. |
| 75 | Heshmat MY 1975 | Case/control selection; retrospective exposure ascertainment; recall and/or social-desirability bias where self-report was used; PSA/healthcare-related detection differences; residual confounding. | Eligible comparative evidence; retrospective exposure ascertainment limits causal-temporal inference. Non-pooling, where applicable, reflects exposure/estimate incompatibility rather than a RoB grade. |

**Notes.** PCa, prostate cancer; BPH, benign prostatic hyperplasia; HPV, human papillomavirus; STI, sexually transmitted infection. Counts refer to reports rather than necessarily independent source populations. Some reports arose from common parent cohorts or study platforms. Leitzmann 2004 and Rider 2016, for example, represent analyses from the Health Professionals Follow-up Study with the latter extending follow-up. The classification 'tissue-based case/non-cancer comparative study' is a review-level descriptive grouping used to avoid forcing heterogeneous tissue studies into a single case-control label; original design descriptions remain identified where explicit.

**Supplementary Table S3. Meta-analysis summary and figure placement**

| **Figure** | **Analysis set** | **Metric** | **k** | **Effect (95% CI)** | **I²** | **Prediction interval** | **Placement** | **Rationale** |
| --- | --- | --- | --- | --- | --- | --- | --- | --- |
| Figure 2 | HPV tissue/PCR primary | OR | 13 | 3.01 (1.97-4.61) | 36.2% | 1.15-7.91 | Main manuscript | Primary pooled biological-detection result; robust to sensitivity analyses. |
| Supplementary Figure S1 | HPV tissue/PCR sensitivity incl all-zero | OR | 14 | 2.97 (1.97-4.49) | 32.0% | 1.19-7.43 | Supplementary | Sensitivity including Strickler all-zero row. |
| Supplementary Figure S2 | HPV tissue/PCR excluding Michopoulou healthy-tissue control | OR | 12 | 2.95 (1.90-4.60) | 40.4% | 1.06-8.20 | Supplementary | Comparator-type sensitivity. |
| Supplementary Figure S3 | HPV tissue/PCR excluding zero-cell rows | OR | 11 | 2.80 (1.87-4.20) | 32.4% | 1.21-6.49 | Supplementary | Continuity-correction sensitivity. |
| Supplementary Figure S4 | HPV16 tissue/PCR subtype | OR | 4 | 4.90 (2.84-8.46) | 0.0% | 2.84-8.46 | Supplementary | Subtype analysis. |
| Supplementary Figure S5 | HPV18 tissue/PCR subtype primary | OR | 4 | 4.92 (2.44-9.93) | 0.0% | 2.44-9.93 | Supplementary | HPV18 subtype primary analysis using the final extraction. |
| Supplementary Figure S6 | HPV18 tissue/PCR subtype alternative extraction | OR | 4 | 4.69 (2.32-9.48) | 0.0% | 2.32-9.48 | Supplementary | Sensitivity analysis for the alternative Ohadian Moghadam HPV18 extraction. |
| Supplementary Figure S7 | HPV18 tissue/PCR subtype excluding Ohadian | OR | 3 | 4.72 (2.25-9.91) | 0.0% | 2.25-9.91 | Supplementary | Sensitivity excluding source-discrepant Ohadian row. |
| Supplementary Figure S8 | HPV16 serology mixed OR/RR | relative effect | 5 | 1.10 (0.85-1.43) | 31.5% | 0.73-1.67 | Supplementary | Secondary serology analysis; mixed OR/RR. |
| Supplementary Figure S9 | HPV16 serology OR-only | OR | 4 | 1.05 (0.83-1.32) | 18.9% | 0.77-1.42 | Supplementary | OR-only sensitivity for HPV16 serology. |
| Supplementary Figure S10 | HPV18 serology mixed OR/RR | relative effect | 4 | 1.20 (0.71-2.01) | 62.8% | 0.45-3.16 | Supplementary | Secondary serology analysis; mixed OR/RR. |
| Supplementary Figure S11 | HPV18 serology OR-only | OR | 3 | 0.92 (0.66-1.28) | 0.0% | 0.66-1.28 | Supplementary | OR-only sensitivity for HPV18 serology. |
| Supplementary Figure S12 | T. vaginalis serology total PCa primary | OR | 3 | 1.37 (0.87-2.14) | 70.9% | 0.63-2.96 | Supplementary | Exploratory secondary pooled result. |
| Supplementary Figure S13 | T. vaginalis serology total PCa Saleh sensitivity | OR | 3 | 1.44 (0.87-2.38) | 76.9% | 0.59-3.52 | Supplementary | Sensitivity using Saleh text-value denominator. |
| Supplementary Figure S14 | T. vaginalis serology advanced/aggressive subgroup | OR | 2 | 0.70 (0.42-1.19) | 54.8% |  | Supplementary | Exploratory advanced/aggressive subgroup. |
| Supplementary Figure S15 | Self-reported/history-based any STI exploratory | OR | 2 | 1.51 (0.66-3.49) | 81.6% |  | Supplementary | Exploratory history/self-reported STI model. |
| Supplementary Figure S16 | Specific STI descriptive estimates | OR | 3 | Descriptive only | NA | Not applicable | Supplementary | Descriptive-only; no pooled estimate. |
| Supplementary Figure S17 | Sexual behavior descriptive estimates | relative effect | 5 | Descriptive only | NA | Not applicable | Supplementary | Descriptive-only; exposure contrasts heterogeneous. |
| Supplementary Figure S18 | Other infection descriptive estimates | relative effect | 2 | Descriptive only | NA | Not applicable | Supplementary | Descriptive-only; distinct infections. |
| Supplementary Figure S19 | Funnel plot: HPV tissue/PCR primary small-study effects | OR | 13 | Not applicable | Not applicable | Not applicable | Supplementary | Funnel plot and Egger’s regression test for the primary HPV tissue/PCR model. |

Summary of the primary, sensitivity, subtype, serology, STI-related, and descriptive analyses reported in the manuscript and supplementary figures.

**Supplementary Table S4. Study-level data used in final forest plots**

| **Figure** | **Analysis set** | **Study** | **Exposure** | **Effect type** | **Effect** | **LCI** | **UCI** | **Case +** | **Case total** | **Control +** | **Control total** | **Weight %** | **Notes** |
| --- | --- | --- | --- | --- | --- | --- | --- | --- | --- | --- | --- | --- | --- |
| Figure 2 | HPV tissue/PCR primary | Leiros 2005 | Any HPV DNA | OR | 43.57 | 2.49 | 761.61 | 17 | 41 | 0 | 30 | 2.0 | Zero-cell control group; continuity correction required |
| Figure 2 | HPV tissue/PCR primary | Carozzi 2004 | High-risk HPV DNA | OR | 4.67 | 1.34 | 16.24 | 14 | 26 | 5 | 25 | 7.8 | Use high-risk row rather than any-HPV row |
| Figure 2 | HPV tissue/PCR primary | Singh 2015 | Any HPV L1 DNA | OR | 2.79 | 1.28 | 6.06 | 39 | 95 | 11 | 55 | 13.3 | Main Indian tissue PCR row |
| Figure 2 | HPV tissue/PCR primary | Abdolmaleki 2018 | HPV18 / HPV by general primers | OR | 2.64 | 0.23 | 29.88 | 2 | 58 | 1 | 75 | 2.7 | Low-prevalence study; wide CI |
| Figure 2 | HPV tissue/PCR primary | Babakhani 2017 | Any HPV DNA | OR | 1.65 | 0.53 | 5.15 | 9 | 40 | 6 | 40 | 8.8 | Nested PCR + sequencing |
| Figure 2 | HPV tissue/PCR primary | Medel-Flores 2018 | High-risk HPV DNA | OR | 2.30 | 1.23 | 4.31 | 37 | 189 | 16 | 167 | 15.7 | Reported OR about 2.3 |
| Figure 2 | HPV tissue/PCR primary | Ahmed 2023 | High-risk HPV DNA | OR | 1.21 | 0.33 | 4.47 | 5 | 14 | 11 | 35 | 7.3 | Small biopsy study; benign comparator includes inflammatory benign samples |
| Figure 2 | HPV tissue/PCR primary | Huang 2016 | HPV16/18 DNA by PCR-RDB | OR | 3.77 | 1.62 | 8.78 | 26 | 75 | 9 | 73 | 12.3 | PCR-RDB preferred over IHC |
| Figure 2 | HPV tissue/PCR primary | Todorova 2025 | HPV DNA | OR | 0.48 | 0.11 | 2.03 | 3 | 49 | 6 | 50 | 6.3 | HPV null/reverse study |
| Figure 2 | HPV tissue/PCR primary | Rodríguez-Romero 2025 | Any HPV DNA | OR | 6.81 | 2.78 | 16.70 | 109 | 117 | 40 | 60 | 11.6 | High-prevalence Mexico qPCR study |
| Figure 2 | HPV tissue/PCR primary | Michopoulou 2014 | Any HPV DNA by GP5+/6+ real-time PCR | OR | 5.52 | 0.66 | 46.58 | 8 | 50 | 1 | 30 | 3.4 | Final adjudicated extraction; healthy tissue controls; comparator sensitivity considered. |
| Figure 2 | HPV tissue/PCR primary | Salehi 2012 | Any HPV DNA | OR | 9.14 | 0.46 | 180.02 | 3 | 68 | 0 | 85 | 1.9 | Final adjudicated extraction; zero-cell control; continuity correction required. |
| Figure 2 | HPV tissue/PCR primary | Ohadian Moghadam 2021 | Any HPV L1 DNA by PCR/hybridization | OR | 5.57 | 1.41 | 21.99 | 12 | 35 | 3 | 35 | 6.8 | HPV L1 12/35 PCa vs 3/35 BPH controls. |
| Supplementary Figure S1 | HPV tissue/PCR sensitivity incl all-zero | Leiros 2005 | Any HPV DNA | OR | 43.57 | 2.49 | 761.61 | 17 | 41 | 0 | 30 | 1.9 | Zero-cell control group; continuity correction required |
| Supplementary Figure S1 | HPV tissue/PCR sensitivity incl all-zero | Carozzi 2004 | High-risk HPV DNA | OR | 4.67 | 1.34 | 16.24 | 14 | 26 | 5 | 25 | 7.6 | Use high-risk row rather than any-HPV row |
| Supplementary Figure S1 | HPV tissue/PCR sensitivity incl all-zero | Singh 2015 | Any HPV L1 DNA | OR | 2.79 | 1.28 | 6.06 | 39 | 95 | 11 | 55 | 13.4 | Main Indian tissue PCR row |
| Supplementary Figure S1 | HPV tissue/PCR sensitivity incl all-zero | Abdolmaleki 2018 | HPV18 / HPV by general primers | OR | 2.64 | 0.23 | 29.88 | 2 | 58 | 1 | 75 | 2.6 | Low-prevalence study; wide CI |
| Supplementary Figure S1 | HPV tissue/PCR sensitivity incl all-zero | Babakhani 2017 | Any HPV DNA | OR | 1.65 | 0.53 | 5.15 | 9 | 40 | 6 | 40 | 8.6 | Nested PCR + sequencing |
| Supplementary Figure S1 | HPV tissue/PCR sensitivity incl all-zero | Medel-Flores 2018 | High-risk HPV DNA | OR | 2.30 | 1.23 | 4.31 | 37 | 189 | 16 | 167 | 16.0 | Reported OR about 2.3 |
| Supplementary Figure S1 | HPV tissue/PCR sensitivity incl all-zero | Ahmed 2023 | High-risk HPV DNA | OR | 1.21 | 0.33 | 4.47 | 5 | 14 | 11 | 35 | 7.2 | Small biopsy study; benign comparator includes inflammatory benign samples |
| Supplementary Figure S1 | HPV tissue/PCR sensitivity incl all-zero | Huang 2016 | HPV16/18 DNA by PCR-RDB | OR | 3.77 | 1.62 | 8.78 | 26 | 75 | 9 | 73 | 12.3 | PCR-RDB preferred over IHC |
| Supplementary Figure S1 | HPV tissue/PCR sensitivity incl all-zero | Todorova 2025 | HPV DNA | OR | 0.48 | 0.11 | 2.03 | 3 | 49 | 6 | 50 | 6.2 | HPV null/reverse study |
| Supplementary Figure S1 | HPV tissue/PCR sensitivity incl all-zero | Rodríguez-Romero 2025 | Any HPV DNA | OR | 6.81 | 2.78 | 16.70 | 109 | 117 | 40 | 60 | 11.5 | High-prevalence Mexico qPCR study |
| Supplementary Figure S1 | HPV tissue/PCR sensitivity incl all-zero | Michopoulou 2014 | Any HPV DNA by GP5+/6+ real-time PCR | OR | 5.52 | 0.66 | 46.58 | 8 | 50 | 1 | 30 | 3.3 | Final adjudicated extraction; healthy tissue controls; comparator sensitivity considered. |
| Supplementary Figure S1 | HPV tissue/PCR sensitivity incl all-zero | Salehi 2012 | Any HPV DNA | OR | 9.14 | 0.46 | 180.02 | 3 | 68 | 0 | 85 | 1.8 | Final adjudicated extraction; zero-cell control; continuity correction required. |
| Supplementary Figure S1 | HPV tissue/PCR sensitivity incl all-zero | Ohadian Moghadam 2021 | Any HPV L1 DNA by PCR/hybridization | OR | 5.57 | 1.41 | 21.99 | 12 | 35 | 3 | 35 | 6.6 | HPV L1 12/35 PCa vs 3/35 BPH controls. |
| Supplementary Figure S1 | HPV tissue/PCR sensitivity incl all-zero | Strickler 1998 | Any HPV DNA by PCR | OR | 0.97 | 0.02 | 49.58 | 0 | 63 | 0 | 61 | 1.1 | All-zero HPV detection row; little statistical information. |
| Supplementary Figure S2 | HPV tissue/PCR excluding Michopoulou healthy-tissue control | Leiros 2005 | Any HPV DNA | OR | 43.57 | 2.49 | 761.61 | 17 | 41 | 0 | 30 | 2.2 | Zero-cell control group; continuity correction required |
| Supplementary Figure S2 | HPV tissue/PCR excluding Michopoulou healthy-tissue control | Carozzi 2004 | High-risk HPV DNA | OR | 4.67 | 1.34 | 16.24 | 14 | 26 | 5 | 25 | 8.2 | Use high-risk row rather than any-HPV row |
| Supplementary Figure S2 | HPV tissue/PCR excluding Michopoulou healthy-tissue control | Singh 2015 | Any HPV L1 DNA | OR | 2.79 | 1.28 | 6.06 | 39 | 95 | 11 | 55 | 13.6 | Main Indian tissue PCR row |
| Supplementary Figure S2 | HPV tissue/PCR excluding Michopoulou healthy-tissue control | Abdolmaleki 2018 | HPV18 / HPV by general primers | OR | 2.64 | 0.23 | 29.88 | 2 | 58 | 1 | 75 | 2.9 | Low-prevalence study; wide CI |
| Supplementary Figure S2 | HPV tissue/PCR excluding Michopoulou healthy-tissue control | Babakhani 2017 | Any HPV DNA | OR | 1.65 | 0.53 | 5.15 | 9 | 40 | 6 | 40 | 9.1 | Nested PCR + sequencing |
| Supplementary Figure S2 | HPV tissue/PCR excluding Michopoulou healthy-tissue control | Medel-Flores 2018 | High-risk HPV DNA | OR | 2.30 | 1.23 | 4.31 | 37 | 189 | 16 | 167 | 15.8 | Reported OR about 2.3 |
| Supplementary Figure S2 | HPV tissue/PCR excluding Michopoulou healthy-tissue control | Ahmed 2023 | High-risk HPV DNA | OR | 1.21 | 0.33 | 4.47 | 5 | 14 | 11 | 35 | 7.7 | Small biopsy study; benign comparator includes inflammatory benign samples |
| Supplementary Figure S2 | HPV tissue/PCR excluding Michopoulou healthy-tissue control | Huang 2016 | HPV16/18 DNA by PCR-RDB | OR | 3.77 | 1.62 | 8.78 | 26 | 75 | 9 | 73 | 12.6 | PCR-RDB preferred over IHC |
| Supplementary Figure S2 | HPV tissue/PCR excluding Michopoulou healthy-tissue control | Todorova 2025 | HPV DNA | OR | 0.48 | 0.11 | 2.03 | 3 | 49 | 6 | 50 | 6.7 | HPV null/reverse study |
| Supplementary Figure S2 | HPV tissue/PCR excluding Michopoulou healthy-tissue control | Rodríguez-Romero 2025 | Any HPV DNA | OR | 6.81 | 2.78 | 16.70 | 109 | 117 | 40 | 60 | 11.9 | High-prevalence Mexico qPCR study |
| Supplementary Figure S2 | HPV tissue/PCR excluding Michopoulou healthy-tissue control | Salehi 2012 | Any HPV DNA | OR | 9.14 | 0.46 | 180.02 | 3 | 68 | 0 | 85 | 2.0 | Final adjudicated extraction; zero-cell control; continuity correction required. |
| Supplementary Figure S2 | HPV tissue/PCR excluding Michopoulou healthy-tissue control | Ohadian Moghadam 2021 | Any HPV L1 DNA by PCR/hybridization | OR | 5.57 | 1.41 | 21.99 | 12 | 35 | 3 | 35 | 7.2 | HPV L1 12/35 PCa vs 3/35 BPH controls. |
| Supplementary Figure S3 | HPV tissue/PCR excluding zero-cell rows | Carozzi 2004 | High-risk HPV DNA | OR | 4.67 | 1.34 | 16.24 | 14 | 26 | 5 | 25 | 7.8 | Use high-risk row rather than any-HPV row |
| Supplementary Figure S3 | HPV tissue/PCR excluding zero-cell rows | Singh 2015 | Any HPV L1 DNA | OR | 2.79 | 1.28 | 6.06 | 39 | 95 | 11 | 55 | 14.3 | Main Indian tissue PCR row |
| Supplementary Figure S3 | HPV tissue/PCR excluding zero-cell rows | Abdolmaleki 2018 | HPV18 / HPV by general primers | OR | 2.64 | 0.23 | 29.88 | 2 | 58 | 1 | 75 | 2.6 | Low-prevalence study; wide CI |
| Supplementary Figure S3 | HPV tissue/PCR excluding zero-cell rows | Babakhani 2017 | Any HPV DNA | OR | 1.65 | 0.53 | 5.15 | 9 | 40 | 6 | 40 | 8.9 | Nested PCR + sequencing |
| Supplementary Figure S3 | HPV tissue/PCR excluding zero-cell rows | Medel-Flores 2018 | High-risk HPV DNA | OR | 2.30 | 1.23 | 4.31 | 37 | 189 | 16 | 167 | 17.5 | Reported OR about 2.3 |
| Supplementary Figure S3 | HPV tissue/PCR excluding zero-cell rows | Ahmed 2023 | High-risk HPV DNA | OR | 1.21 | 0.33 | 4.47 | 5 | 14 | 11 | 35 | 7.3 | Small biopsy study; benign comparator includes inflammatory benign samples |
| Supplementary Figure S3 | HPV tissue/PCR excluding zero-cell rows | Huang 2016 | HPV16/18 DNA by PCR-RDB | OR | 3.77 | 1.62 | 8.78 | 26 | 75 | 9 | 73 | 13.1 | PCR-RDB preferred over IHC |
| Supplementary Figure S3 | HPV tissue/PCR excluding zero-cell rows | Todorova 2025 | HPV DNA | OR | 0.48 | 0.11 | 2.03 | 3 | 49 | 6 | 50 | 6.2 | HPV null/reverse study |
| Supplementary Figure S3 | HPV tissue/PCR excluding zero-cell rows | Rodríguez-Romero 2025 | Any HPV DNA | OR | 6.81 | 2.78 | 16.70 | 109 | 117 | 40 | 60 | 12.2 | High-prevalence Mexico qPCR study |
| Supplementary Figure S3 | HPV tissue/PCR excluding zero-cell rows | Michopoulou 2014 | Any HPV DNA by GP5+/6+ real-time PCR | OR | 5.52 | 0.66 | 46.58 | 8 | 50 | 1 | 30 | 3.2 | Final adjudicated extraction; healthy tissue controls; comparator sensitivity considered. |
| Supplementary Figure S3 | HPV tissue/PCR excluding zero-cell rows | Ohadian Moghadam 2021 | Any HPV L1 DNA by PCR/hybridization | OR | 5.57 | 1.41 | 21.99 | 12 | 35 | 3 | 35 | 6.8 | HPV L1 12/35 PCa vs 3/35 BPH controls. |
| Supplementary Figure S4 | HPV16 tissue/PCR subtype | Singh 2015 | HPV16 DNA | OR | 8.00 | 2.31 | 27.69 | 30 | 95 | 3 | 55 | 19.3 | HPV subtype extraction used for final analysis. |
| Supplementary Figure S4 | HPV16 tissue/PCR subtype | Huang 2016 | HPV16 DNA by PCR-RDB | OR | 3.27 | 1.21 | 8.85 | 17 | 75 | 6 | 73 | 30.1 | HPV subtype extraction used for final analysis. |
| Supplementary Figure S4 | HPV16 tissue/PCR subtype | Rodríguez-Romero 2025 | HPV16 DNA | OR | 4.69 | 2.12 | 10.41 | 53 | 117 | 9 | 60 | 47.0 | HPV subtype extraction used for final analysis. |
| Supplementary Figure S4 | HPV16 tissue/PCR subtype | Ohadian Moghadam 2021 | HPV16 DNA | OR | 18.68 | 1.02 | 341.24 | 7 | 35 | 0 | 35 | 3.5 | HPV subtype extraction used for final analysis. |
| Supplementary Figure S5 | HPV18 tissue/PCR subtype primary | Singh 2015 | HPV18 DNA | OR | 3.64 | 0.43 | 31.06 | 6 | 95 | 1 | 55 | 10.7 | HPV subtype extraction used for final analysis. |
| Supplementary Figure S5 | HPV18 tissue/PCR subtype primary | Huang 2016 | HPV18 DNA by PCR-RDB | OR | 4.89 | 1.33 | 17.97 | 13 | 75 | 3 | 73 | 29.1 | HPV subtype extraction used for final analysis. |
| Supplementary Figure S5 | HPV18 tissue/PCR subtype primary | Rodríguez-Romero 2025 | HPV18 DNA | OR | 4.89 | 1.81 | 13.24 | 36 | 117 | 5 | 60 | 49.7 | HPV subtype extraction used for final analysis. |
| Supplementary Figure S5 | HPV18 tissue/PCR subtype primary | Ohadian Moghadam 2021 | HPV18 DNA | OR | 7.03 | 0.80 | 61.87 | 6 | 35 | 1 | 35 | 10.4 | HPV subtype extraction used for final analysis. |
| Supplementary Figure S6 | HPV18 tissue/PCR subtype alternative extraction | Singh 2015 | HPV18 DNA | OR | 3.64 | 0.43 | 31.06 | 6 | 95 | 1 | 55 | 10.8 | HPV subtype extraction used for final analysis. |
| Supplementary Figure S6 | HPV18 tissue/PCR subtype alternative extraction | Huang 2016 | HPV18 DNA by PCR-RDB | OR | 4.89 | 1.33 | 17.97 | 13 | 75 | 3 | 73 | 29.3 | HPV subtype extraction used for final analysis. |
| Supplementary Figure S6 | HPV18 tissue/PCR subtype alternative extraction | Rodríguez-Romero 2025 | HPV18 DNA | OR | 4.89 | 1.81 | 13.24 | 36 | 117 | 5 | 60 | 50.0 | HPV subtype extraction used for final analysis. |
| Supplementary Figure S6 | HPV18 tissue/PCR subtype alternative extraction | Ohadian Moghadam 2021 | HPV18 DNA | OR | 4.39 | 0.46 | 41.41 | 4 | 35 | 1 | 35 | 9.9 | Alternative from Table 2 discrepancy: HPV18 cases 4/35 vs controls 1/35. |
| Supplementary Figure S7 | HPV18 tissue/PCR subtype excluding Ohadian | Singh 2015 | HPV18 DNA | OR | 3.64 | 0.43 | 31.06 | 6 | 95 | 1 | 55 | 12.0 | HPV subtype extraction used for final analysis. |
| Supplementary Figure S7 | HPV18 tissue/PCR subtype excluding Ohadian | Huang 2016 | HPV18 DNA by PCR-RDB | OR | 4.89 | 1.33 | 17.97 | 13 | 75 | 3 | 73 | 32.5 | HPV subtype extraction used for final analysis. |
| Supplementary Figure S7 | HPV18 tissue/PCR subtype excluding Ohadian | Rodríguez-Romero 2025 | HPV18 DNA | OR | 4.89 | 1.81 | 13.24 | 36 | 117 | 5 | 60 | 55.5 | HPV subtype extraction used for final analysis. |
| Supplementary Figure S8 | HPV16 serology mixed OR/RR | Korodi 2005 | HPV16 seropositivity | OR | 0.90 | 0.64 | 1.26 |  |  |  |  | 31.4 | Nordic biobank serology study |
| Supplementary Figure S8 | HPV16 serology mixed OR/RR | Sutcliffe/PCPT 2010 | HPV16 strong seropositivity | OR | 1.07 | 0.77 | 1.48 |  |  |  |  | 32.6 | PCPT nested serology study |
| Supplementary Figure S8 | HPV16 serology mixed OR/RR | Hisada 2000 | HPV16 antibody positivity in stored serum | OR | 2.70 | 0.90 | 7.90 | 20 | 48 | 19 | 63 | 5.3 | JAMA research letter; overlapping duplicate report excluded from analysis. |
| Supplementary Figure S8 | HPV16 serology mixed OR/RR | Dillner 1998 | HPV16 seropositivity | RR | 2.58 | 0.77 | 8.56 | 7 | 165 | 5 | 290 | 4.4 | Finnish serum bank nested case-control study. |
| Supplementary Figure S8 | HPV16 serology mixed OR/RR | Rosenblatt 2003 | HPV16 seropositivity | OR | 1.06 | 0.71 | 1.57 | 59 | 642 | 50 | 570 | 26.2 | Population-based serology study. |
| Supplementary Figure S9 | HPV16 serology OR-only | Korodi 2005 | HPV16 seropositivity | OR | 0.90 | 0.64 | 1.26 |  |  |  |  | 33.7 | Nordic biobank serology study |
| Supplementary Figure S9 | HPV16 serology OR-only | Sutcliffe/PCPT 2010 | HPV16 strong seropositivity | OR | 1.07 | 0.77 | 1.48 |  |  |  |  | 35.5 | PCPT nested serology study |
| Supplementary Figure S9 | HPV16 serology OR-only | Hisada 2000 | HPV16 antibody positivity in stored serum | OR | 2.70 | 0.90 | 7.90 | 20 | 48 | 19 | 63 | 4.3 | JAMA research letter; overlapping duplicate report excluded from analysis. |
| Supplementary Figure S9 | HPV16 serology OR-only | Rosenblatt 2003 | HPV16 seropositivity | OR | 1.06 | 0.71 | 1.57 | 59 | 642 | 50 | 570 | 26.4 | Population-based serology study. |
| Supplementary Figure S10 | HPV18 serology mixed OR/RR | Korodi 2005 | HPV18 seropositivity | OR | 0.79 | 0.49 | 1.26 |  |  |  |  | 30.3 | Nordic biobank serology study |
| Supplementary Figure S10 | HPV18 serology mixed OR/RR | Sutcliffe/PCPT 2010 | HPV18 strong seropositivity | OR | 0.87 | 0.47 | 1.63 |  |  |  |  | 25.6 | PCPT nested serology study |
| Supplementary Figure S10 | HPV18 serology mixed OR/RR | Dillner 1998 | HPV18 seropositivity | RR | 2.88 | 1.27 | 6.56 | 17 | 165 | 13 | 290 | 20.1 | Finnish serum bank nested case-control study. |
| Supplementary Figure S10 | HPV18 serology mixed OR/RR | Rosenblatt 2003 | HPV18 seropositivity | OR | 1.36 | 0.69 | 2.69 | 22 | 642 | 14 | 570 | 23.9 | Population-based serology study. |
| Supplementary Figure S11 | HPV18 serology OR-only | Korodi 2005 | HPV18 seropositivity | OR | 0.79 | 0.49 | 1.26 |  |  |  |  | 48.6 | Nordic biobank serology study |
| Supplementary Figure S11 | HPV18 serology OR-only | Sutcliffe/PCPT 2010 | HPV18 strong seropositivity | OR | 0.87 | 0.47 | 1.63 |  |  |  |  | 28.0 | PCPT nested serology study |
| Supplementary Figure S11 | HPV18 serology OR-only | Rosenblatt 2003 | HPV18 seropositivity | OR | 1.36 | 0.69 | 2.69 | 22 | 642 | 14 | 570 | 23.4 | Population-based serology study. |
| Supplementary Figure S12 | T. vaginalis serology total PCa primary | Sutcliffe/HPFS 2006 | T. vaginalis A-actinin IgG seropositivity | OR | 1.43 | 1.00 | 2.03 |  |  |  |  | 38.2 | Compatible total-PCa serology row. |
| Supplementary Figure S12 | T. vaginalis serology total PCa primary | Marous/PLCO 2017 | T. vaginalis seropositivity | OR | 0.97 | 0.73 | 1.27 |  |  |  |  | 42.1 | Compatible total-PCa serology row. |
| Supplementary Figure S12 | T. vaginalis serology total PCa primary | Saleh/Egypt 2021 | T. vaginalis IgG seropositivity corrected primary | OR_2x2 | 2.59 | 1.18 | 5.68 | 24 | 126 | 10 | 120 | 19.7 | Corrected primary based on 19% of 126 PCa cases. |
| Supplementary Figure S13 | T. vaginalis serology total PCa Saleh sensitivity | Sutcliffe/HPFS 2006 | T. vaginalis A-actinin IgG seropositivity | OR | 1.43 | 1.00 | 2.03 |  |  |  |  | 37.6 | Compatible total-PCa serology row. |
| Supplementary Figure S13 | T. vaginalis serology total PCa Saleh sensitivity | Marous/PLCO 2017 | T. vaginalis seropositivity | OR | 0.97 | 0.73 | 1.27 |  |  |  |  | 40.5 | Compatible total-PCa serology row. |
| Supplementary Figure S13 | T. vaginalis serology total PCa Saleh sensitivity | Saleh/Egypt 2021 | T. vaginalis IgG seropositivity text 26/120 | OR_2x2 | 3.04 | 1.40 | 6.63 | 26 | 120 | 10 | 120 | 21.9 | Sensitivity only using internally inconsistent 26/120 text. |
| Supplementary Figure S14 | T. vaginalis serology advanced/aggressive subgroup | Marous/PLCO 2017 | T. vaginalis seropositivity, more advanced PCa | OR | 0.88 | 0.59 | 1.30 |  |  |  |  | 58.9 | Advanced disease subgroup. |
| Supplementary Figure S14 | T. vaginalis serology advanced/aggressive subgroup | Shui 2016 | T. vaginalis serostatus, advanced/fatal PCa | OR | 0.51 | 0.28 | 0.93 |  |  |  |  | 41.1 | Advanced/fatal endpoint only. |
| Supplementary Figure S15 | Self-reported/history-based any STI exploratory | Sawaya/EPICAP 2024 | Overall sexually transmitted infections | OR | 1.05 | 0.86 | 1.29 |  |  |  |  | 57.7 | History-based self-report/medical-history estimate. |
| Supplementary Figure S15 | Self-reported/history-based any STI exploratory | Ángeles-Garay 2019 | History of sexually transmitted disease | OR | 2.49 | 1.24 | 4.99 |  |  |  |  | 42.3 | History-based STI estimate; different case-control setting. |
| Supplementary Figure S16 | Specific STI descriptive estimates | Sawaya/EPICAP 2024 | Self-reported gonorrhea history | OR | 0.90 | 0.61 | 1.33 |  |  |  |  |  | Single-study updated row. |
| Supplementary Figure S16 | Specific STI descriptive estimates | Sawaya/EPICAP 2024 | Self-reported trichomonas history | OR | 0.74 | 0.27 | 2.07 |  |  |  |  |  | Self-reported, not serology. |
| Supplementary Figure S16 | Specific STI descriptive estimates | Sawaya/EPICAP 2024 | Self-reported genital herpes history | OR | 0.69 | 0.38 | 1.27 |  |  |  |  |  | Single-study updated row. |
| Supplementary Figure S17 | Sexual behavior descriptive estimates | Honda 1988 | Age at first intercourse <17 vs 21+ | RR | 2.30 | 1.30 | 4.00 |  |  |  |  |  | Do not pool with late sexual debut. |
| Supplementary Figure S17 | Sexual behavior descriptive estimates | Ángeles-Garay 2019 | Beginning sexual relations after 18 years | OR | 2.82 | 1.27 | 6.25 |  |  |  |  |  | Directionally incompatible with early-debut contrast. |
| Supplementary Figure S17 | Sexual behavior descriptive estimates | Ángeles-Garay 2019 | Sexual intercourse with sex workers | OR | 8.75 | 3.66 | 20.90 |  |  |  |  |  | Single-study high-magnitude exposure. |
| Supplementary Figure S17 | Sexual behavior descriptive estimates | Mandel & Schuman 1987 | Partner venereal disease; hospital controls | OR | 2.71 | 1.14 | 6.46 |  |  |  |  |  | Primary control-group choice if analyzed. |
| Supplementary Figure S17 | Sexual behavior descriptive estimates | Mandel & Schuman 1987 | Partner venereal disease; neighborhood controls | OR | 2.09 | 1.02 | 4.29 |  |  |  |  |  | Alternate-control sensitivity. |
| Supplementary Figure S18 | Other infection descriptive estimates | McDonald 2011 | HHV-8 seropositivity | HR | 0.88 | 0.46 | 1.69 |  |  |  |  |  | Separate infection exposure. |
| Supplementary Figure S18 | Other infection descriptive estimates | Sawaya/EPICAP 2024 | Overall non-sexually transmitted viral infections | OR | 1.11 | 0.90 | 1.35 |  |  |  |  |  | Separate non-STI exposure class. |

Rows are analysis-specific; repeated studies appear in multiple sensitivity or subtype analyses by design and should not be counted as independent studies across figures.

**Supplementary Table S5. Evidence certainty summary by exposure domain**

| **Evidence domain** | **Studies** | **Main result** | **Risk of bias** | **Inconsistency** | **Indirectness** | **Imprecision** | **Publication bias / small-study effects** | **Overall certainty** | **Interpretation** |
| --- | --- | --- | --- | --- | --- | --- | --- | --- | --- |
| HPV tissue/PCR detection | 13 studies; 14 in all-zero sensitivity | OR 3.01 (95% CI 1.97-4.61); PI 1.15-7.91 | Serious: comparator/tissue-sampling differences, limited temporal clarity, and variable laboratory reporting | Not serious to serious: I² 36.2%, but PI remains wide | Serious: tissue/PCR positivity does not establish timing, persistence, or causality | Not serious for primary estimate, but some study estimates sparse | Possible; small and positive tissue studies may be overrepresented | Very low | Local HPV DNA signal is consistent but causal interpretation is not justified. |
| HPV16/18 tissue/PCR subtype | 4 studies for HPV16; 4 studies for HPV18 | HPV16 OR 4.90; HPV18 OR 4.92 | Serious | Not serious in pooled model (I² 0%) but k is small | Serious: subtype assays and tissue sources vary | Serious: few studies | Undetected small-study effects cannot be excluded | Very low | Supportive subtype signal; should not be overinterpreted. |
| HPV16/18 serology | 5 mixed HPV16; 4 mixed HPV18; OR-only sensitivities smaller | HPV16 relative effect 1.10; HPV18 relative effect 1.20 | Serious | Serious for HPV18 mixed analysis; lower for OR-only sensitivity | Serious: serology reflects systemic past exposure, not prostate tissue infection | Serious: confidence intervals cross null | Possible | Very low | No clear serological association; biologically distinct from tissue/PCR results. |
| T. vaginalis serology | 3 total-PCa studies | OR 1.37 (95% CI 0.87-2.14); PI 0.63-2.96 | Serious | Serious: I² 70.9% | Serious: serology and endpoint definitions vary | Serious: CI and PI include null | Possible | Very low | Exploratory only. |
| History-based/self-reported any STI | 2 studies | OR 1.51 (95% CI 0.66-3.49) | Serious: self-report and recall/healthcare access bias | Serious: I² 81.6% | Serious: composite STI exposure definitions differ | Very serious: k=2 and wide CI | Possible | Very low | Insufficient for firm inference. |
| Sexual behavior-related exposures | Descriptive only | No pooled estimate | Serious: recall and confounding likely | Very serious: contrasts and definitions heterogeneous | Serious: behavioral proxies do not map directly to infection biology | Serious: sparse per contrast | Possible | Very low | Do not combine into a single pooled estimate. |

Certainty was assessed using GRADE. Observational evidence was initially considered low certainty and was then evaluated across standard GRADE domains. Overall certainty was very low for each exposure domain; these ratings do not imply causal effects.

# **Supplementary Table S6. Full-text reports excluded after eligibility assessment**

The table lists 64 reports excluded after full-text eligibility assessment and final revision-stage eligibility reconciliation. Primary exclusion reasons were consolidated from the screening record and the two source-verified eligibility corrections made during revision. Counts by reason code are: E3, n=11; E4, n=16; E5, n=2; E6, n=14; E7, n=8; and E9, n=13.

| **No.** | **Branch** | **Record ID** | **Title** | **Year** | **Reason code** | **Primary reason** | **Detailed rationale** |
| --- | --- | --- | --- | --- | --- | --- | --- |
| 1 | Database branch | 24 | Comparative analysis of human papillomavirus detection in tissue and urinary/plasmatic exosomes in prostatic adenocarcinoma: Links with clinicopathological parameters | 2026 | E9 | Wrong outcome or endpoint not incident/risk association | Endpoint, outcome, or analytic question did not match prostate cancer risk/incidence association. |
| 2 | Database branch | 53 | HPV and urological cancers: Investigating renal and prostate cancer associations | 2026 | E3 | No usable risk estimate / no extractable 2x2 data | No extractable comparative effect estimate or 2x2 data for the planned risk synthesis. |
| 3 | Database branch | 10 | Sexually Transmitted Infections in Prostate Cancer: A Prospective Multicenter Analysis | 2026 | E4 | Review, commentary, letter, news, or other non-primary report | Non-primary report type; no independent original eligible dataset for this review question. |
| 4 | Database branch | 71 | Evaluation of human papillomavirus as a risk factor in prostate cancer pathogenesis | 2025 | E4 | Review, commentary, letter, news, or other non-primary report | Non-primary report type; no independent original eligible dataset for this review question. |
| 5 | Database branch | 113 | Exposing the Influence of Preceding Sexual Behavior in Younger Years toward Prostate Cancer Occurrence: Qualitative Mapping on Sexual Risk Factors | 2025 | E4 | Review, commentary, letter, news, or other non-primary report | Non-primary report type; no independent original eligible dataset for this review question. |
| 6 | Database branch | 186 | Human Papillomavirus Infection and prostate cancer in Mexico | 2025 | E6 | Duplicate publication, overlapping population, or superseded report | Conference/supplement abstract or overlapping report. |
| 7 | Database branch | 76 | Prostate cancer and HPV: Investigating the association with case control approach | 2025 | E3 | No usable risk estimate / no extractable 2x2 data | No extractable comparative effect estimate or 2x2 data for the planned risk synthesis. |
| 8 | Database branch | 178 | Reduction of Prostate Cancer Risk: Role of Frequent Ejaculation-Associated Mechanisms | 2025 | E4 | Review, commentary, letter, news, or other non-primary report | Non-primary report type; no independent original eligible dataset for this review question. |
| 9 | Database branch | 251 | Highly sensitive deep panel sequencing of 27 HPV genotypes in prostate cancer biopsies results in very low detection rates and indicates that HPV is not a major etiological driver of this malignancy | 2024 | E3 | No usable risk estimate / no extractable 2x2 data | No extractable comparative effect estimate or 2x2 data for the planned risk synthesis. |
| 10 | Database branch | 330 | The impact of obesity and sexual behavior on prostate cancer risk is mediated by testosterone levels: a mendelian randomization study and mediation analysis | 2024 | E9 | Wrong outcome or endpoint not incident/risk association | Genetic/effect-modification or mechanistic analysis not usable as a planned risk estimate. |
| 11 | Database branch | 511 | Human herpesvirus 8 infection is associated with prostate cancer among IFNL4-DeltaG carriers | 2023 | E9 | Wrong outcome or endpoint not incident/risk association | Genetic/effect-modification analysis not usable as a planned risk estimate. |
| 12 | Database branch | 462 | No Association of Trichomonas vaginalis Seropositivity with Advanced Prostate Cancer Risk in the Multiethnic Cohort: A Nested Case-Control Study | 2023 | E7 | No independent non-cancer control group / unsuitable comparator | Endpoint/comparator configuration not suitable for the prespecified main risk synthesis. |
| 13 | Database branch | 972 | Association between Chlamydia trachomatis Infection and Prostate Cancer: A Case-Control Study | 2021 | E4 | Review, commentary, letter, news, or other non-primary report | Non-primary report type; no independent original eligible dataset for this review question. |
| 14 | Database branch | 870 | Comparison of Sexual Behavior and Inflammatory Parameters in Prostate Cancer Patients with Control Group: Prospective Controlled Study | 2021 | E3 | No usable risk estimate / no extractable 2x2 data | No extractable comparative effect estimate or 2x2 data for the planned risk synthesis. |
| 15 | Database branch | 995 | Interactive effect of TLR SNPs and exposure to sexually transmitted infections on prostate cancer risk in Jamaican men | 2020 | E9 | Wrong outcome or endpoint not incident/risk association | Gene-exposure interaction analysis not used as an independent main risk estimate. |
| 16 | Database branch | 1202 | Association between Trichomonas vaginalis and prostate cancer mortality | 2019 | E9 | Wrong outcome or endpoint not incident/risk association | Mortality endpoint rather than incident/risk association. |
| 17 | Database branch | 1318 | Human Papillomavirus (HPV) Infection As an Emerging Risk Factor in Prostate Cancer | 2018 | E5 | Conference abstract only / non-full report / full text unavailable for eligibility | Abstract-only/non-full report; no reliable complete full text located. |
| 18 | Database branch | 1422 | IFNL4-DeltaG is associated with prostate cancer among men at increased risk of sexually transmitted infections | 2018 | E9 | Wrong outcome or endpoint not incident/risk association | Genetic/effect-modification analysis not usable as a planned risk estimate. |
| 19 | Database branch | 1303 | New Insights into Ejaculatory Frequency and Prostate Cancer Risk: Association, Causation, or What Do We Have to Lose? | 2018 | E4 | Review, commentary, letter, news, or other non-primary report | Non-primary report type; no independent original eligible dataset for this review question. |
| 20 | Database branch | 1532 | Adult body size, sexual history and adolescent sexual development, may predict risk of developing prostate cancer: Results from the New South Wales Lifestyle and Evaluation of Risk Study (CLEAR) | 2017 | E3 | No usable risk estimate / no extractable 2x2 data | No extractable comparative effect estimate or 2x2 data for the planned risk synthesis. |
| 21 | Database branch | 1448 | Relationship of Human Papilloma Virus (HPV) and Epstein Barr Virus (EBV) with prostate cancer in Al-Najaf governorate | 2017 | E3 | No usable risk estimate / no extractable 2x2 data | No extractable comparative effect estimate or 2x2 data for the planned risk synthesis. |
| 22 | Database branch | 1615 | Evidence for Masturbation and Prostate Cancer Risk: Do We Have a Verdict? | 2016 | E4 | Review, commentary, letter, news, or other non-primary report | Non-primary report type; no independent original eligible dataset for this review question. |
| 23 | Database branch | 1596 | Frequent ejaculations reduce the risk of prostate cancer | 2016 | E6 | Duplicate publication, overlapping population, or superseded report | Duplicate, commentary, or secondary report of ejaculation-frequency evidence. |
| 24 | Database branch | 1588 | Trichomonas vaginalis: a possible foe to prostate cancer | 2016 | E4 | Review, commentary, letter, news, or other non-primary report | Non-primary report type; no independent original eligible dataset for this review question. |
| 25 | Database branch | 3884 | [Prostate cancer: papillomaviruses as a possible cause] | 2015 | E4 | Review, commentary, letter, news, or other non-primary report | Non-primary report type; no independent original eligible dataset for this review question. |
| 26 | Database branch | 2002 | Detection of human papillomavirus (HPV) DNA prevalence and p53 codon 72 (Arg72Pro) polymorphism in prostate cancer in a Greek group of patients | 2014 | E6 | Duplicate publication, overlapping population, or superseded report | Duplicate/overlapping report or metadata-mismatched duplicate of retained Michopoulou 2014 full article. |
| 27 | Database branch | 2162 | False-positive prostate cancer markers in a man with symptomatic urethral Chlamydia trachomatis infection | 2013 | E9 | Wrong outcome or endpoint not incident/risk association | Wrong outcome/endpoint. |
| 28 | Database branch | 2139 | Human papillomavirus and Epstein Barr virus in prostate cancer: Koilocytes indicate potential oncogenic influences of human papillomavirus in prostate cancer | 2013 | E7 | No independent non-cancer control group / unsuitable comparator | No independent non-cancer control or unsuitable comparator for risk synthesis. |
| 29 | Database branch | 2048 | Oncogenic herpesvirus HHV-8 promotes androgen-independent prostate cancer growth | 2013 | E9 | Wrong outcome or endpoint not incident/risk association | Mechanistic/experimental endpoint rather than incident/risk association. |
| 30 | Database branch | 2169 | Prospective study of effect modification by Toll-like receptor 4 variation on the association between Trichomonas vaginalis serostatus and prostate cancer | 2013 | E9 | Wrong outcome or endpoint not incident/risk association | Effect-modification analysis not used as an independent main risk estimate. |
| 31 | Database branch | 2107 | Urologic characteristics and sexual behaviors associated with prostate cancer in an african-caribbean population in Barbados, West Indies | 2013 | E3 | No usable risk estimate / no extractable 2x2 data | No extractable comparative effect estimate or 2x2 data for the planned risk synthesis. |
| 32 | Database branch | 2485 | Correlation of hepatitis C and prostate cancer, inverse correlation of basal cell hyperplasia or prostatitis and epidemic syphilis of unknown duration | 2011 | E3 | No usable risk estimate / no extractable 2x2 data | No extractable comparative effect estimate or 2x2 data for the planned risk synthesis. |
| 33 | Database branch | 2446 | No high-risk human papillomavirus infection in prostate cancer tissues | 2011 | E7 | No independent non-cancer control group / unsuitable comparator | No independent non-cancer control or unsuitable comparator. |
| 34 | Database branch | 2678 | The role of human papillomavirus infection in prostate carcinoma | 2011 | E4 | Review, commentary, letter, news, or other non-primary report | Non-primary report type; no independent original eligible dataset for this review question. |
| 35 | Database branch | 2627 | Association between human herpesvirus 8 infection, inflammation and a polymorphism in the IL-6 signaling receptor in increased prostate cancer risk among men of African descent | 2010 | E9 | Wrong outcome or endpoint not incident/risk association | Genetic/effect-modification analysis not usable as a planned risk estimate. |
| 36 | Database branch | 2591 | The significance of chronic prostatitis for the etiopathology of prostate cancer | 2010 | E3 | No usable risk estimate / no extractable 2x2 data | No extractable comparative effect estimate or 2x2 data for the planned risk synthesis. |
| 37 | Database branch | 2829 | Is prostate cancer an HPV-associated lesion? | 2009 | E3 | No usable risk estimate / no extractable 2x2 data | No extractable comparative effect estimate or 2x2 data for the planned risk synthesis. |
| 38 | Database branch | 2765 | Trichomonosis and subsequent risk of prostate cancer in the Prostate Cancer Prevention Trial | 2009 | E3 | No usable risk estimate / no extractable 2x2 data | No extractable comparative effect estimate or 2x2 data for the planned risk synthesis. |
| 39 | Database branch | 2865 | Involvement of human papillomavirus infections in prostate cancer progression | 2008 | E9 | Wrong outcome or endpoint not incident/risk association | Progression endpoint rather than incident/risk association. |
| 40 | Database branch | 2955 | Human herpesvirus 8 seroprevalence among prostate cancer case patients and control subjects | 2007 | E6 | Duplicate publication, overlapping population, or superseded report | Overlapping HHV-8 serology report; independent retained report used where applicable. |
| 41 | Database branch | 2934 | Prevalence of BK virus and human papillomavirus in human prostate cancer | 2007 | E7 | No independent non-cancer control group / unsuitable comparator | No independent non-cancer control or unsuitable comparator. |
| 42 | Database branch | 3146 | Sexual behaviour, history of sexually transmitted diseases, and the risk of prostate cancer: A case-control study in Cuba | 2005 | E4 | Review, commentary, letter, news, or other non-primary report | Non-primary report type; no independent original eligible dataset for this review question. |
| 43 | Database branch | 3130 | Sexually transmitted diseases and other urogenital conditions as risk factors for prostate cancer: A case-control study in Wayne County, Michigan | 2005 | E4 | Review, commentary, letter, news, or other non-primary report | Non-primary report type; no independent original eligible dataset for this review question. |
| 44 | Database branch | 3901 | Frequency of ejaculation and risk of prostate cancer | 2004 | E6 | Duplicate publication, overlapping population, or superseded report | Duplicate/abstract/commentary of ejaculation-frequency evidence. |
| 45 | Database branch | 4938 | Frequent ejaculation may be linked to decreased risk of prostate cancer | 2004 | E6 | Duplicate publication, overlapping population, or superseded report | Duplicate/abstract/commentary of ejaculation-frequency evidence. |
| 46 | Database branch | 3250 | Sexual factors and prostate cancer | 2003 | E6 | Duplicate publication, overlapping population, or superseded report | Duplicate/overlapping record of retained Mandel & Schuman 1987 full article. |
| 47 | Database branch | 3335 | Sexual activity and the risk of prostate cancer [4] | 2002 | E4 | Review, commentary, letter, news, or other non-primary report | Letter/commentary/non-primary report; no original eligible data. |
| 48 | Database branch | 3396 | Sexual factors and the risk of prostate cancer | 2001 | E6 | Duplicate publication, overlapping population, or superseded report | Duplicate/overlapping alternate title for retained Mandel & Schuman 1987 full article. |
| 49 | Database branch | 3431 | Inactive free: Total prostate specific antigen ratios in ejaculate from men with suspected and known prostate cancer, compared with young control men | 2000 | E9 | Wrong outcome or endpoint not incident/risk association | Biomarker endpoint rather than incident/risk association. |
| 50 | Database branch | 3555 | The molecular biology of prostate cancer morbidity and mortality: Accelerated death from ejaculate poisoning? | 1997 | E4 | Review, commentary, letter, news, or other non-primary report | Non-primary report type; no independent original eligible dataset for this review question. |
| 51 | Database branch | 4023 | Human papillomavirus in prostatic cancer: no evidence found by in situ DNA hybridization | 1991 | E7 | No independent non-cancer control group / unsuitable comparator | No independent non-cancer control or unsuitable comparator. |
| 52 | Database branch | 4895 | Sexually and Non-sexually Transmitted Infections and The risk of Prostate Cancer | 2023 | E6 | Duplicate publication, overlapping population, or superseded report | Duplicate/overlapping record of retained Sawaya 2024 EPICAP full article. |
| 53 | Database branch | 2031 | The ejaculatory ducts and their implications in prostate adenocarcinoma | 2013 | E9 | Wrong outcome or endpoint not incident/risk association | Wrong outcome/endpoint. |
| 54 | Database branch | 4901 | Detection of Human papilloma virus (HPV) DNA prevalence and p53 codon 72 (Arg72Pro) polymorphism in prostate cancer of Greek patients | 2012 | E6 | Duplicate publication, overlapping population, or superseded report | Preliminary/abstract/overlapping report superseded by retained Michopoulou 2014 full article. |
| 55 | Database branch | 2983 | The role of human papillomavirus infection in prostate cancer | 2007 | E4 | Review, commentary, letter, news, or other non-primary report | Non-primary report type; no independent original eligible dataset for this review question. |
| 56 | Database branch | 4906 | Ejaculation frequency and risk of prostate cancer | 2004 | E6 | Duplicate publication, overlapping population, or superseded report | Duplicate/abstract/commentary of ejaculation-frequency evidence. |
| 57 | Database branch | 4908 | Ejaculation frequency in relation to risk of prostate cancer. | 2003 | E6 | Duplicate publication, overlapping population, or superseded report | Duplicate/abstract/commentary of ejaculation-frequency evidence. |
| 58 | Database branch | 3301 | Chlamydia lowers prostate cancer risk. | 2002 | E5 | Conference abstract only / non-full report / full text unavailable for eligibility | News/commentary/brief non-primary report. |
| 59 | Database branch | 3459 | Human papillomavirus antibody and risk of prostate cancer [7] | 2000 | E6 | Duplicate publication, overlapping population, or superseded report | Duplicate/overlap of retained Hisada 2000 JAMA HPV antibody record. |
| 60 | Database branch | 3503 | Increased levels of human papillomavirus type 16 DNA in a subset of prostate cancers | 1999 | E7 | No independent non-cancer control group / unsuitable comparator | No independent non-cancer control or unsuitable comparator. |
| 61 | Database branch | 3671 | Presence of ras oncogene mutations and human papillomavirus DNA in human prostate carcinomas | 1992 | E7 | No independent non-cancer control group / unsuitable comparator | No independent non-cancer control or unsuitable comparator. |
| 62 | Other-method branch | 2633 | Circulating sex hormones play no role in the association between sexual activity and the risk of prostate cancer [secondary abstract] | 2010 | E6 | Duplicate publication, overlapping population, or superseded report | Secondary abstract/conference-type record overlaps with the retained database full article; not an independent included report. |
| 63 | Revision-stage eligibility reconciliation | — | Epidemiologic association between prostatitis and prostate cancer | 2002 | E4 | Review, meta-analysis, or other non-primary report | Secondary meta-analysis of previously published epidemiological studies; no independent primary observational dataset for the review question. |
| 64 | Revision-stage eligibility reconciliation | — | Lack of detection of human papillomavirus DNA in prostate carcinomas in patients from northeastern Brazil | 2016 | E7 | No independent non-cancer control group / unsuitable comparator | Prostate-cancer tissue series without an independent non-cancer comparator/reference group; therefore did not meet the registered comparator requirement. |

Abbreviations: E3, no usable risk estimate/no extractable 2x2 data; E4, non-primary report; E5, abstract-only/non-full report/full text unavailable; E6, duplicate/overlap/superseded report; E7, no independent non-cancer control or unsuitable comparator; E9, wrong outcome or endpoint.
