# Supplementary material for: Sexual behavior-related exposures, sexually transmitted infections, and viral markers in relation to prostate cancer risk: a systematic review and meta-analysis
Source: Front Oncol. 2026 Oct 8;16:1968570. doi: 10.3389/fonc.2026.1968570 (PMC13686856; doi:10.3389/fonc.2026.1968570)
Supplement: Supplementary file 3 [file Table2.docx]

| **Section and Topic** | **Item #** | **Checklist item** | **Location where item is reported** |
| --- | --- | --- | --- |
| **TITLE** | | |  |
| Title | 1 | Identify the report as a systematic review. | Main manuscript, title page. |
| **ABSTRACT** | | |  |
| Abstract | 2 | See the PRISMA 2020 for Abstracts checklist. | Main manuscript, structured Abstract, including Registration. |
| **INTRODUCTION** | | |  |
| Rationale | 3 | Describe the rationale for the review in the context of existing knowledge. | Main manuscript, Introduction: rationale. |
| Objectives | 4 | Provide an explicit statement of the objective(s) or question(s) the review addresses. | Main manuscript, Introduction: objectives. |
| **METHODS** | | |  |
| Eligibility criteria | 5 | Specify the inclusion and exclusion criteria for the review and how studies were grouped for the syntheses. | Main manuscript, Methods: eligibility criteria and exposure grouping. |
| Information sources | 6 | Specify all databases, registers, websites, organisations, reference lists and other sources searched or consulted to identify studies. Specify the date when each source was last searched or consulted. | Main manuscript, Methods: search strategy and information sources. |
| Search strategy | 7 | Present the full search strategies for all databases, registers and websites, including any filters and limits used. | Main manuscript, Methods: search strategy and information sources; supplementary search-strategy file. |
| Selection process | 8 | Specify the methods used to decide whether a study met the inclusion criteria of the review, including how many reviewers screened each record and each report retrieved, whether they worked independently, and if applicable, details of automation tools used in the process. | Main manuscript, Methods: study selection and data extraction; Results: study selection; Figure 1. |
| Data collection process | 9 | Specify the methods used to collect data from reports, including how many reviewers collected data from each report, whether they worked independently, any processes for obtaining or confirming data from study investigators, and if applicable, details of automation tools used in the process. | Main manuscript, Methods: study selection and data extraction. |
| Data items | 10a | List and define all outcomes for which data were sought. Specify whether all results that were compatible with each outcome domain in each study were sought (e.g. for all measures, time points, analyses), and if not, the methods used to decide which results to collect. | Main manuscript, Methods: eligibility criteria, exposure grouping and analytic hierarchy. |
|  | 10b | List and define all other variables for which data were sought (e.g. participant and intervention characteristics, funding sources). Describe any assumptions made about any missing or unclear information. | Main manuscript, Methods: study selection and data extraction; Supplementary Table S1. |
| Study risk of bias assessment | 11 | Specify the methods used to assess risk of bias in the included studies, including details of the tool(s) used, how many reviewers assessed each study and whether they worked independently, and if applicable, details of automation tools used in the process. | Main manuscript, Methods: risk of bias and evidence appraisal; Supplementary Table S2. A structured domain-based appraisal informed by NOS domains was used; no composite numeric score was calculated. |
| Effect measures | 12 | Specify for each outcome the effect measure(s) (e.g. risk ratio, mean difference) used in the synthesis or presentation of results. | Main manuscript, Methods: exposure grouping and analytic hierarchy; Data synthesis and statistical analysis. |
| Synthesis methods | 13a | Describe the processes used to decide which studies were eligible for each synthesis (e.g. tabulating the study intervention characteristics and comparing against the planned groups for each synthesis (item #5)). | Main manuscript, Methods: exposure grouping and analytic hierarchy; Supplementary Tables S1 and S3. |
|  | 13b | Describe any methods required to prepare the data for presentation or synthesis, such as handling of missing summary statistics, or data conversions. | Main manuscript, Methods: study selection and data extraction; Data synthesis and statistical analysis. |
|  | 13c | Describe any methods used to tabulate or visually display results of individual studies and syntheses. | Main manuscript, Results; Fig. 2; Supplementary Figs. S1-S19; Supplementary Table S4. |
|  | 13d | Describe any methods used to synthesize results and provide a rationale for the choice(s). If meta-analysis was performed, describe the model(s), method(s) to identify the presence and extent of statistical heterogeneity, and software package(s) used. | Main manuscript, Methods: data synthesis and statistical analysis. |
|  | 13e | Describe any methods used to explore possible causes of heterogeneity among study results (e.g. subgroup analysis, meta-regression). | Not applicable. No subgroup analysis or meta-regression was conducted because study numbers within comparable exposure groups were insufficient. Sensitivity analyses are reported under item 13f. |
|  | 13f | Describe any sensitivity analyses conducted to assess robustness of the synthesized results. | Main manuscript, Methods and Results; Supplementary Table S3. |
| Reporting bias assessment | 14 | Describe any methods used to assess risk of bias due to missing results in a synthesis (arising from reporting biases). | Main manuscript, Methods: funnel plot and Egger’s regression test were used for the primary tissue-based HPV analysis (13 studies). Formal small-study testing was not conducted for smaller domains; potential reporting bias was considered in GRADE. |
| Certainty assessment | 15 | Describe any methods used to assess certainty (or confidence) in the body of evidence for an outcome. | Main manuscript, Methods 2.1 and 2.6; Results 3.6; Supplementary Table S5. GRADE was added after registration as an evidence-interpretation layer and did not alter eligibility or numerical meta-analysis estimates. |
| **RESULTS** | | |  |
| Study selection | 16a | Describe the results of the search and selection process, from the number of records identified in the search to the number of studies included in the review, ideally using a flow diagram. | Main manuscript, Results: study selection; Figure 1. |
|  | 16b | Cite studies that might appear to meet the inclusion criteria, but which were excluded, and explain why they were excluded. | Main manuscript, Results: study selection; Supplementary Table S6. |
| Study characteristics | 17 | Cite each included study and present its characteristics. | Main manuscript, Results 3.2 and Table 2; Supplementary Table S1; eligible primary observational reports cited in references [19-75]. |
| Risk of bias in studies | 18 | Present assessments of risk of bias for each included study. | Main manuscript, Results 3.2 and Table 2; Supplementary Table S2, with report-level appraisal entries for each of the 57 eligible primary observational reports. |
| Results of individual studies | 19 | For all outcomes, present, for each study: (a) summary statistics for each group (where appropriate) and (b) an effect estimate and its precision (e.g. confidence/credible interval), ideally using structured tables or plots. | Main manuscript, Results; Fig. 2; Supplementary Figs. S1-S19; Supplementary Table S4. The final plotted analyses contain 28 unique reports: 25 enter at least one pooled model and 3 additional reports appear only in descriptive forest plots. |
| Results of syntheses | 20a | For each synthesis, briefly summarise the characteristics and risk of bias among contributing studies. | Main manuscript, Results 3.2 and Table 2; Supplementary Tables S1-S3. |
|  | 20b | Present results of all statistical syntheses conducted. If meta-analysis was done, present for each the summary estimate and its precision (e.g. confidence/credible interval) and measures of statistical heterogeneity. If comparing groups, describe the direction of the effect. | Main manuscript, Results; Table 1; Supplementary Table S3. |
|  | 20c | Present results of all investigations of possible causes of heterogeneity among study results. | Not applicable. No subgroup analysis or meta-regression investigating causes of heterogeneity was conducted because comparable groups contained too few studies. |
|  | 20d | Present results of all sensitivity analyses conducted to assess the robustness of the synthesized results. | Main manuscript, Results; Supplementary Table S3. |
| Reporting biases | 21 | Present assessments of risk of bias due to missing results (arising from reporting biases) for each synthesis assessed. | Funnel plot and Egger’s regression test were performed for the primary tissue-based HPV analysis (13 studies). Formal statistical testing was not conducted for other domains with fewer studies, and potential publication bias was considered in result interpretation and GRADE assessment. |
| Certainty of evidence | 22 | Present assessments of certainty (or confidence) in the body of evidence for each outcome assessed. | Main manuscript, Methods 2.6; Results 3.6; Supplementary Table S5. All evaluated domains were rated very low certainty. |
| **DISCUSSION** | | |  |
| Discussion | 23a | Provide a general interpretation of the results in the context of other evidence. | Main manuscript, Discussion: principal findings; comparison with previous reviews. |
|  | 23b | Discuss any limitations of the evidence included in the review. | Main manuscript, Discussion: strengths and limitations. |
|  | 23c | Discuss any limitations of the review processes used. | Main manuscript, Discussion: strengths and limitations. |
|  | 23d | Discuss implications of the results for practice, policy, and future research. | Main manuscript, Discussion: clinical and research implications; Conclusions. |
| **OTHER INFORMATION** | | |  |
| Registration and protocol | 24a | Provide registration information for the review, including register name and registration number, or state that the review was not registered. | Main manuscript, Methods: protocol and reporting. |
|  | 24b | Indicate where the review protocol can be accessed, or state that a protocol was not prepared. | Main manuscript, Methods: protocol and reporting; PROSPERO CRD420261334823. |
|  | 24c | Describe and explain any amendments to information provided at registration or in the protocol. | Main manuscript, Methods 2.1. The revised manuscript also transparently identifies GRADE as a post-registration evidence-interpretation addition; final eligibility reconciliation is described in Results 3.1. |
| Support | 25 | Describe sources of financial or non-financial support for the review, and the role of the funders or sponsors in the review. | Main manuscript, Statements: Funding. |
| Competing interests | 26 | Declare any competing interests of review authors. | Main manuscript, Statements: Competing interests. |
| Availability of data, code and other materials | 27 | Report which of the following are publicly available and where they can be found: template data collection forms; data extracted from included studies; data used for all analyses; analytic code; any other materials used in the review. | Main manuscript, Availability of data and materials; Supplementary Tables S1-S6; Supplementary Material 3. Study-level analysis inputs, summary outputs, analytic R code for Fig. 2 and Supplementary Figs. S1-S19, Egger-test output, figure files, and R session information are supplied. Fig. 1 is a PRISMA reporting diagram and is not generated by the meta-analysis R script. |

*From:*  Page MJ, McKenzie JE, Bossuyt PM, Boutron I, Hoffmann TC, Mulrow CD, et al. The PRISMA 2020 statement: an updated guideline for reporting systematic reviews. BMJ 2021;372:n71. doi: 10.1136/bmj.n71. This work is licensed under CC BY 4.0. To view a copy of this license, visit <https://creativecommons.org/licenses/by/4.0/>
