# Supplementary material for: Sexual behavior-related exposures, sexually transmitted infections, and viral markers in relation to prostate cancer risk: a systematic review and meta-analysis
Source: Front Oncol. 2026 Oct 8;16:1968570. doi: 10.3389/fonc.2026.1968570 (PMC13686856; doi:10.3389/fonc.2026.1968570)
Supplement: Supplementary file 4 [file DataSheet2.zip › Supplementary_Figures_S1-S19.pdf]

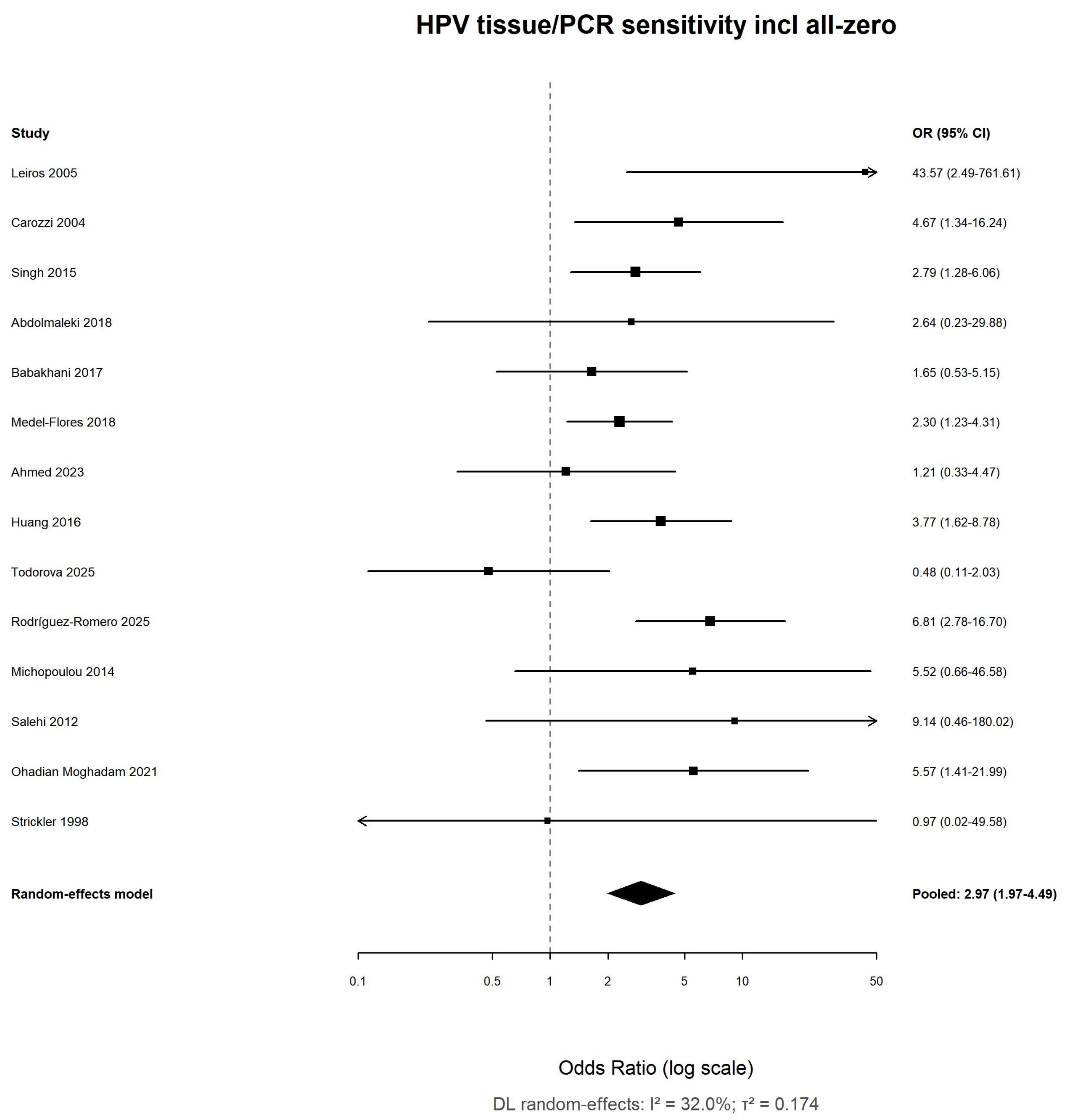
HPV tissue/PCR sensitivity incl all-zero
Study
OR (95% CI)
Leiros 2005
43.57 (2.49-761.61)
Carozzi 2004
4.67 (1.34-16.24)
Singh 2015
2.79 (1.28-6.06)
Abdolmaleki 2018
2.64 (0.23-29.88)
Babakhani 2017
1.65 (0.53-5.15)
Medel-Flores 2018
2.30 (1.23-4.31)
Ahmed 2023
1.21 (0.33-4.47)
Huang 2016
3.77 (1.62-8.78)
Todorova 2025
0.48 (0.11-2.03)
Rodríguez-Romero 2025
6.81 (2.78-16.70)
Michopoulou 2014
5.52 (0.66-46.58)
Salehi 2012
9.14 (0.46-180.02)
Ohadian Moghadam 2021
5.57 (1.41-21.99)
Strickler 1998
0.97 (0.02-49.58)
Random-effects model
Pooled: 2.97 (1.97-4.49)
0.1
0.5
1
2
5
10
50
Odds Ratio (log scale)
DL random-effects: I² = 32.0%; τ² = 0.174

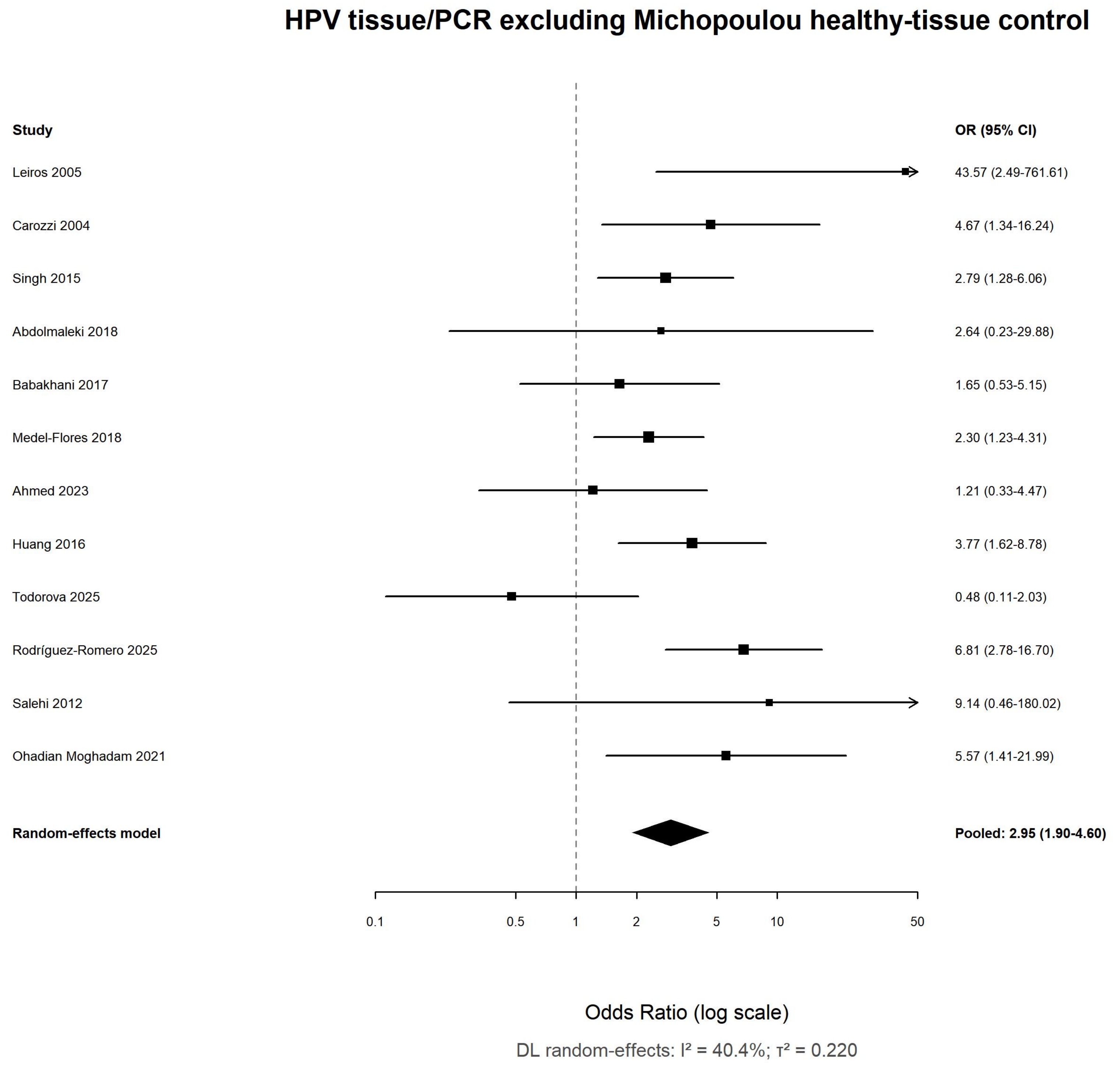
HPV tissue/PCR excluding Michopoulou healthy-tissue control
Study
OR (95% CI)
Leiros 2005
43.57 (2.49-761.61)
Carozzi 2004
4.67 (1.34-16.24)
Singh 2015
2.79 (1.28-6.06)
Abdolmaleki 2018
2.64 (0.23-29.88)
Babakhani 2017
1.65 (0.53-5.15)
Medel-Flores 2018
2.30 (1.23-4.31)
Ahmed 2023
1.21 (0.33-4.47)
Huang 2016
3.77 (1.62-8.78)
Todorova 2025
0.48 (0.11-2.03)
Rodríguez-Romero 2025
6.81 (2.78-16.70)
Salehi 2012
9.14 (0.46-180.02)
Ohadian Moghadam 2021
5.57 (1.41-21.99)
Random-effects model
Pooled: 2.95 (1.90-4.60)
0.1
0.5
1
2
5
10
50
Odds Ratio (log scale)
DL random-effects: I² = 40.4%; τ² = 0.220

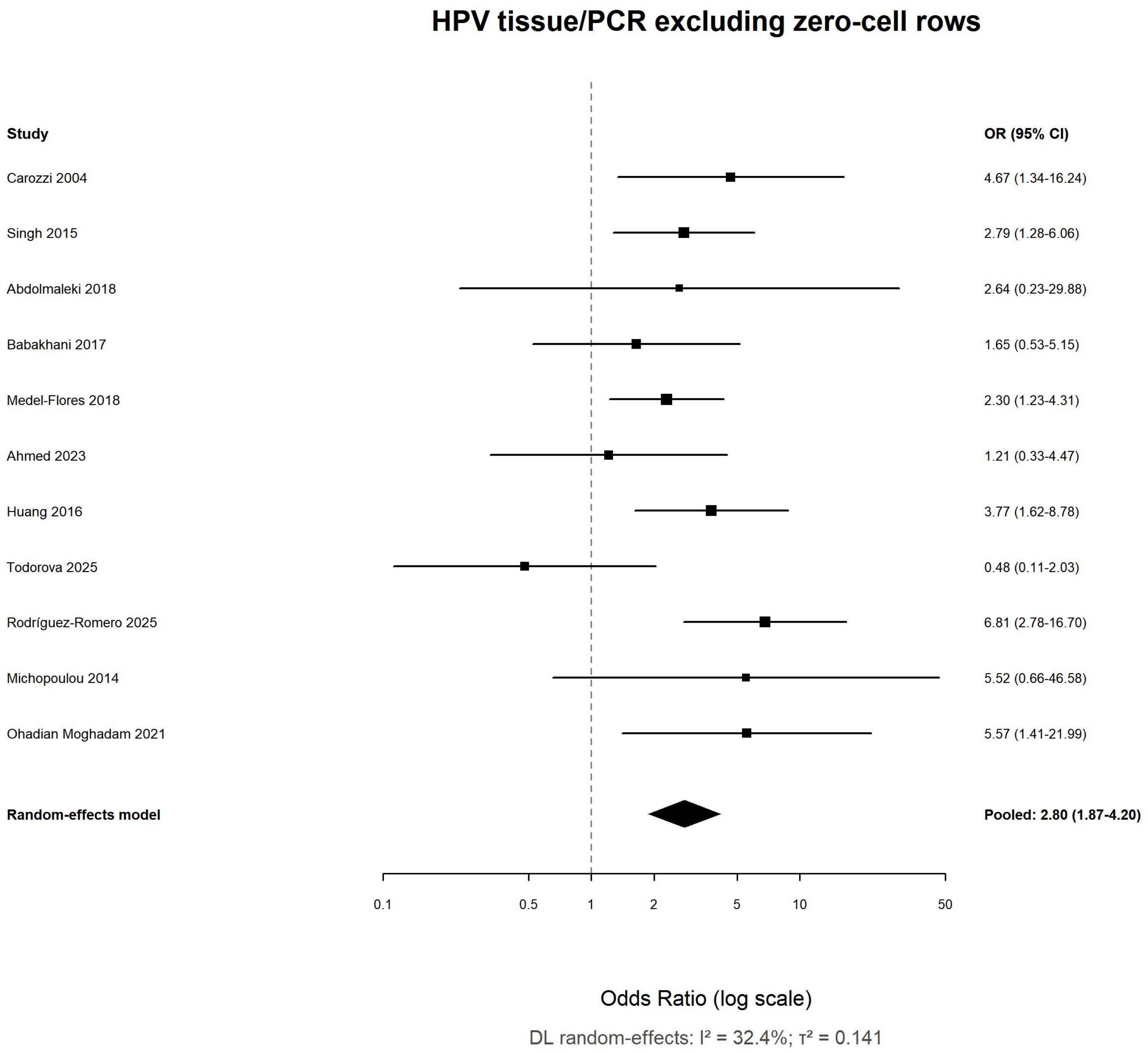

HPV tissue/PCR excluding zero-cell rows
Study
OR (95% CI)
Carozzi 2004
4.67 (1.34-16.24)
Singh 2015
2.79 (1.28-6.06)
Abdolmaleki 2018
2.64 (0.23-29.88)
Babakhani 2017
1.65 (0.53-5.15)
Medel-Flores 2018
2.30 (1.23-4.31)
Ahmed 2023
1.21 (0.33-4.47)
Huang 2016
3.77 (1.62-8.78)
Todorova 2025
0.48 (0.11-2.03)
Rodríguez-Romero 2025
6.81 (2.78-16.70)
Michopoulou 2014
5.52 (0.66-46.58)
Ohadian Moghadam 2021
5.57 (1.41-21.99)
Random-effects model
Pooled: 2.80 (1.87-4.20)
0.1
0.5
1
2
5
10
50
Odds Ratio (log scale)
DL random-effects: I² = 32.4%; τ² = 0.141

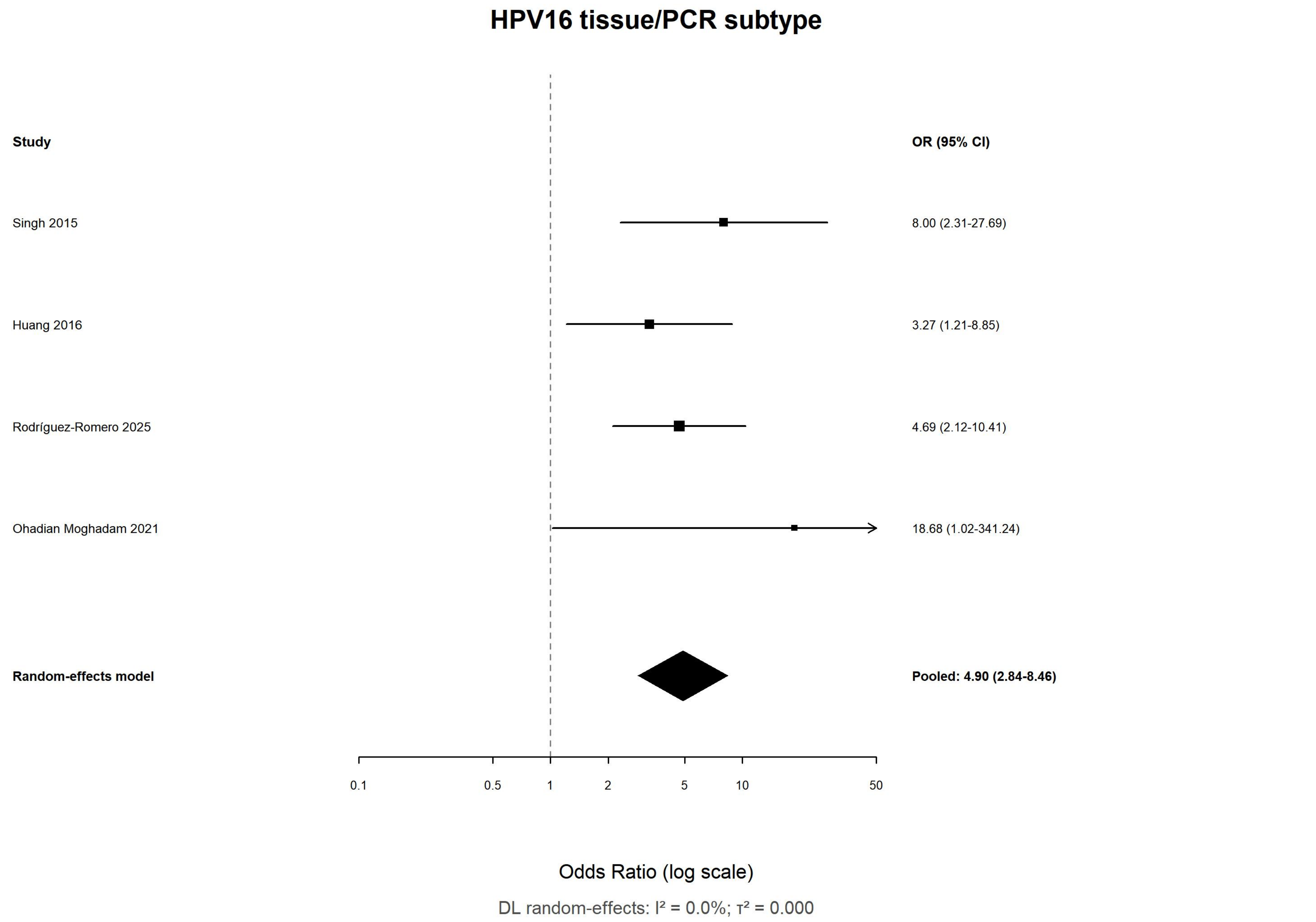
HPV16 tissue/PCR subtype
Study
OR (95% CI)
Singh 2015
8.00 (2.31-27.69)
Huang 2016
3.27 (1.21-8.85)
Rodríguez-Romero 2025
4.69 (2.12-10.41)
Ohadian Moghadam 2021
18.68 (1.02-341.24)
Random-effects model
Pooled: 4.90 (2.84-8.46)
0.1
0.5
1
2
5
10
50
Odds Ratio (log scale)
DL random-effects: I² = 0.0%; τ² = 0.000

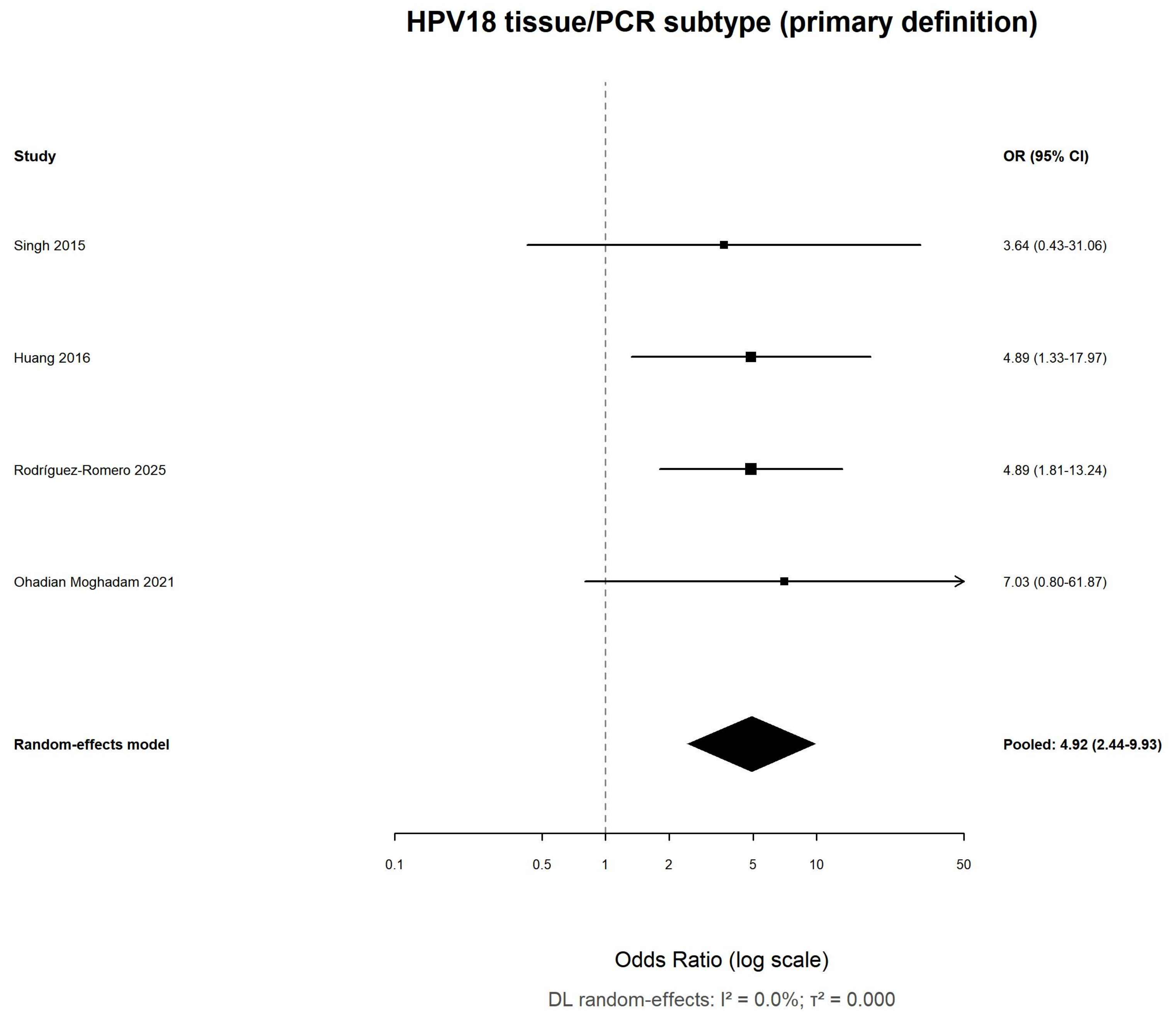

HPV18 tissue/PCR subtype (primary definition)
Study
OR (95% CI)
Singh 2015
3.64 (0.43-31.06)
Huang 2016
4.89 (1.33-17.97)
Rodríguez-Romero 2025
4.89 (1.81-13.24)
Ohadian Moghadam 2021
7.03 (0.80-61.87)
Random-effects model
Pooled: 4.92 (2.44-9.93)
0.1
0.5
1
2
5
10
50
Odds Ratio (log scale)
DL random-effects: I² = 0.0%; τ² = 0.000

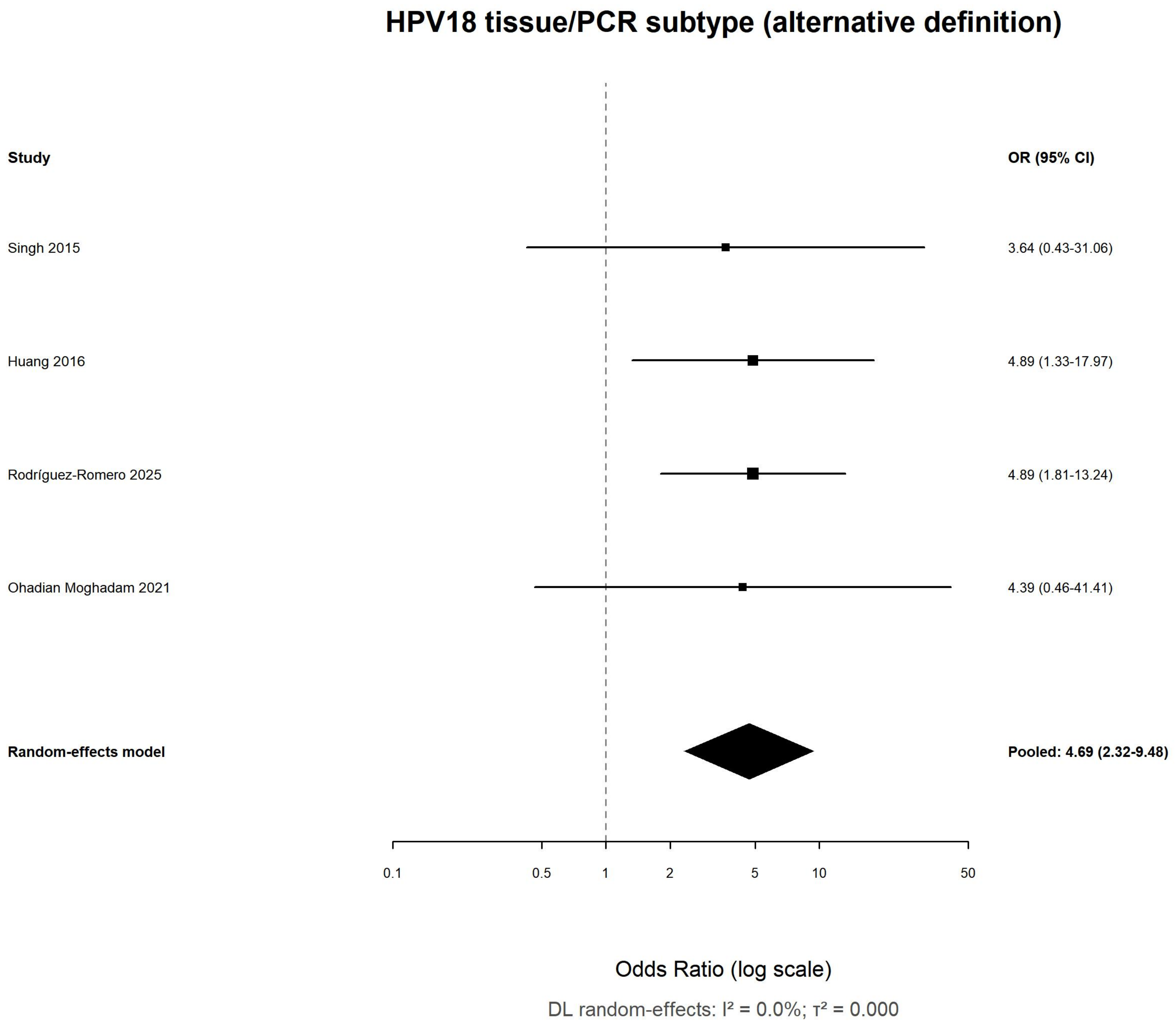
HPV18 tissue/PCR subtype (alternative definition)
Study
OR (95% CI)
Singh 2015
3.64 (0.43-31.06)
Huang 2016
4.89 (1.33-17.97)
Rodríguez-Romero 2025
4.89 (1.81-13.24)
Ohadian Moghadam 2021
4.39 (0.46-41.41)
Random-effects model
Pooled: 4.69 (2.32-9.48)
0.1
0.5
1
2
5
10
50
Odds Ratio (log scale)
DL random-effects: I² = 0.0%; τ² = 0.000

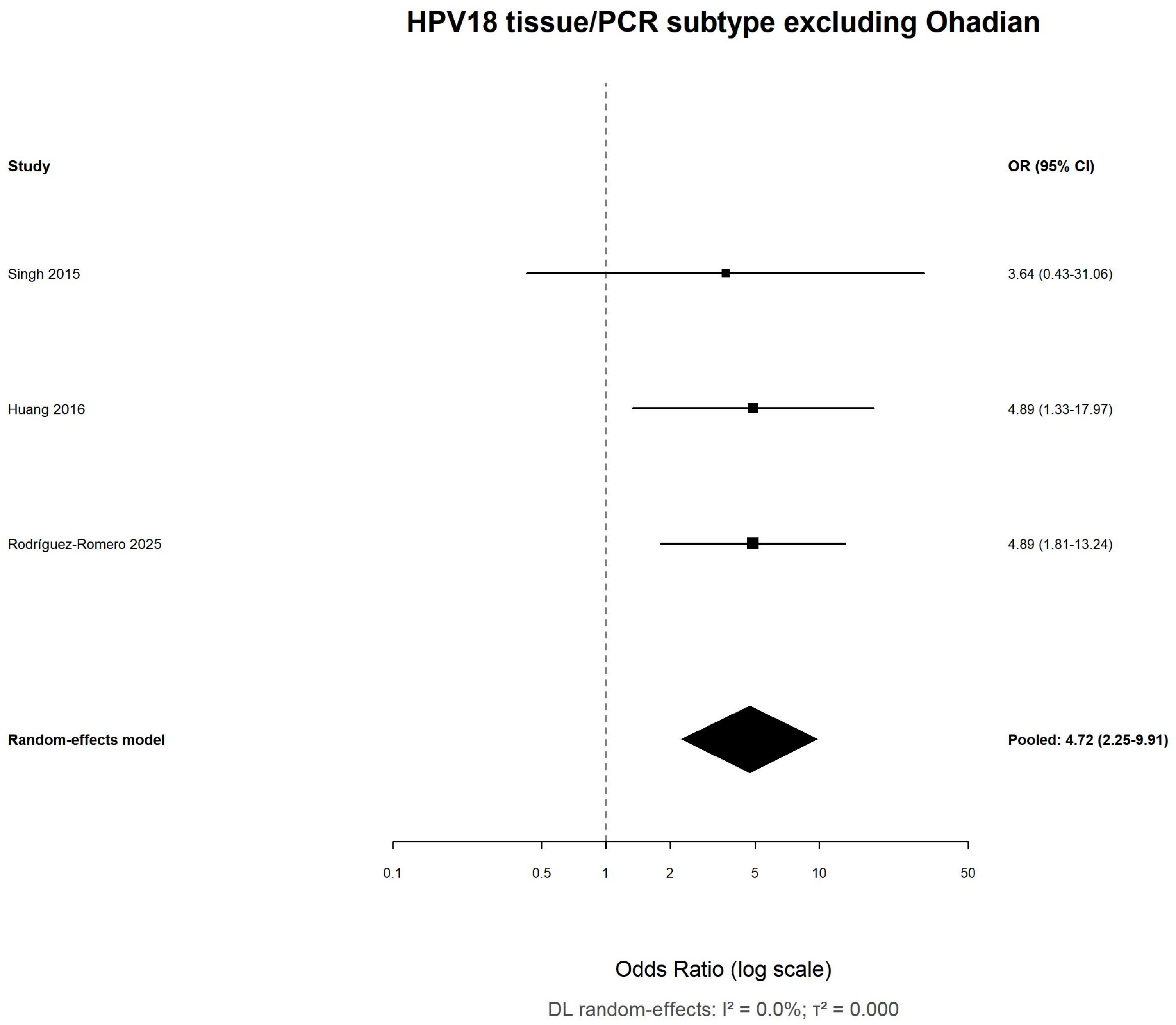

HPV18 tissue/PCR subtype excluding Ohadian
Study
OR (95% CI)
Singh 2015
3.64 (0.43-31.06)
Huang 2016
4.89 (1.33-17.97)
Rodríguez-Romero 2025
4.89 (1.81-13.24)
Random-effects model
Pooled: 4.72 (2.25-9.91)
0.1
0.5
1
2
5
10
50
Odds Ratio (log scale)
DL random-effects: I² = 0.0%; τ² = 0.000

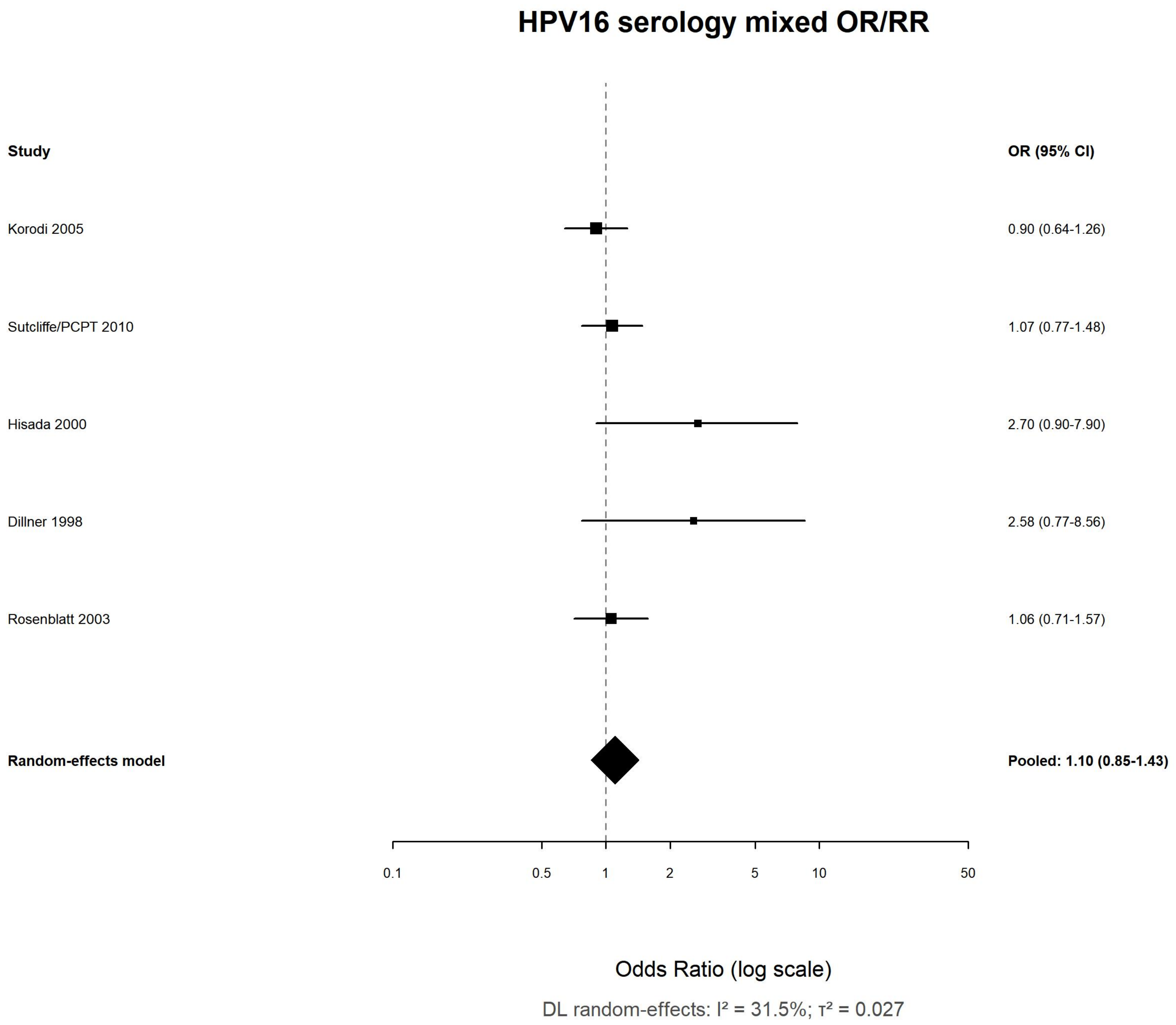
HPV16 serology mixed OR/RR
Study
OR (95% CI)
Korodi 2005
0.90 (0.64-1.26)
Sutcliffe/PCPT 2010
1.07 (0.77-1.48)
Hisada 2000
2.70 (0.90-7.90)
Dillner 1998
2.58 (0.77-8.56)
Rosenblatt 2003
1.06 (0.71-1.57)
Random-effects model
Pooled: 1.10 (0.85-1.43)
0.1
0.5
1
2
5
10
50
Odds Ratio (log scale)
DL random-effects: I² = 31.5%; τ² = 0.027

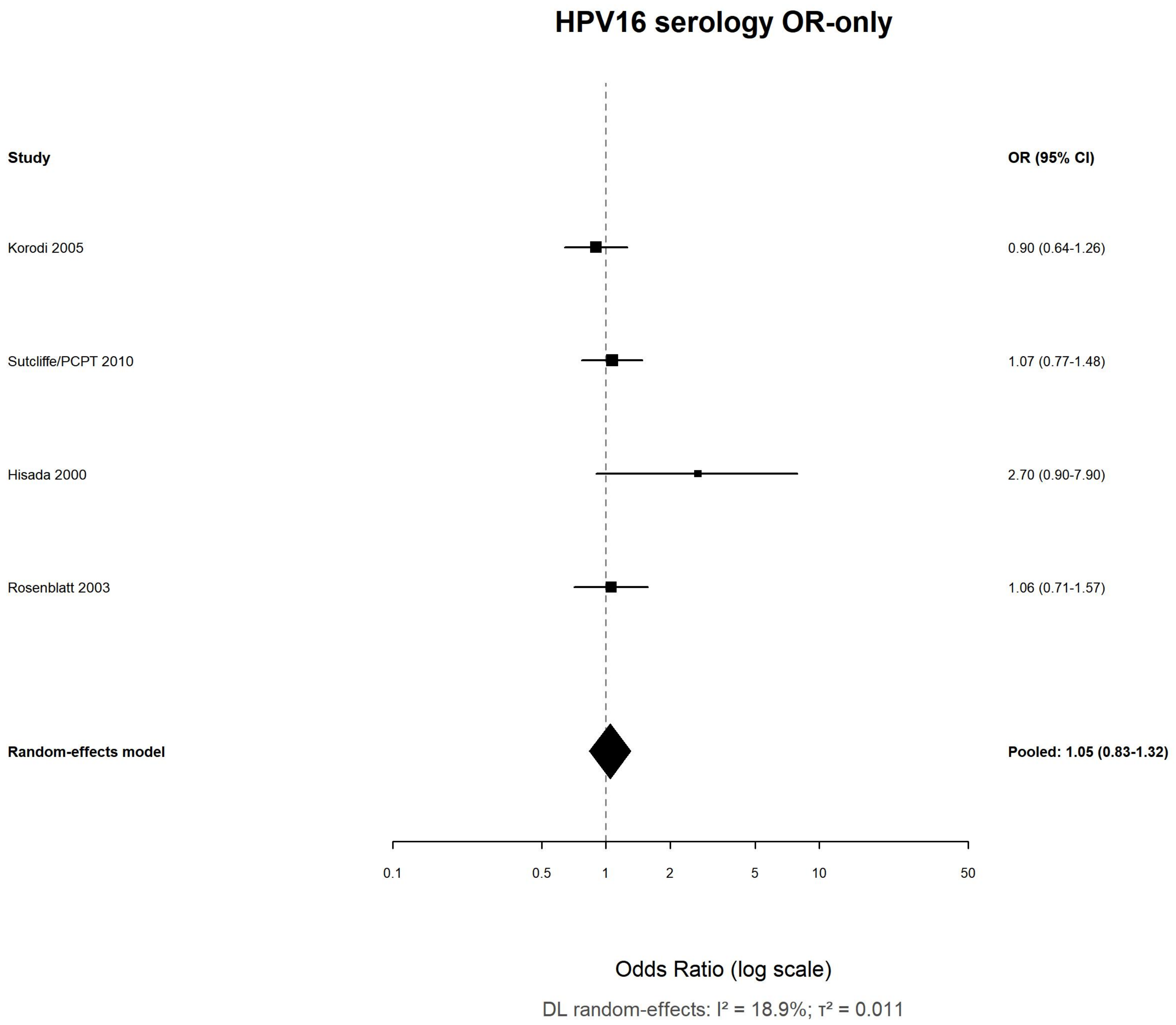
HPV16 serology OR-only
Study
OR (95% CI)
Korodi 2005
0.90 (0.64-1.26)
Sutcliffe/PCPT 2010
1.07 (0.77-1.48)
Hisada 2000
2.70 (0.90-7.90)
Rosenblatt 2003
1.06 (0.71-1.57)
Random-effects model
Pooled: 1.05 (0.83-1.32)
0.1
0.5
1
2
5
10
50
Odds Ratio (log scale)
DL random-effects: I² = 18.9%; τ² = 0.011

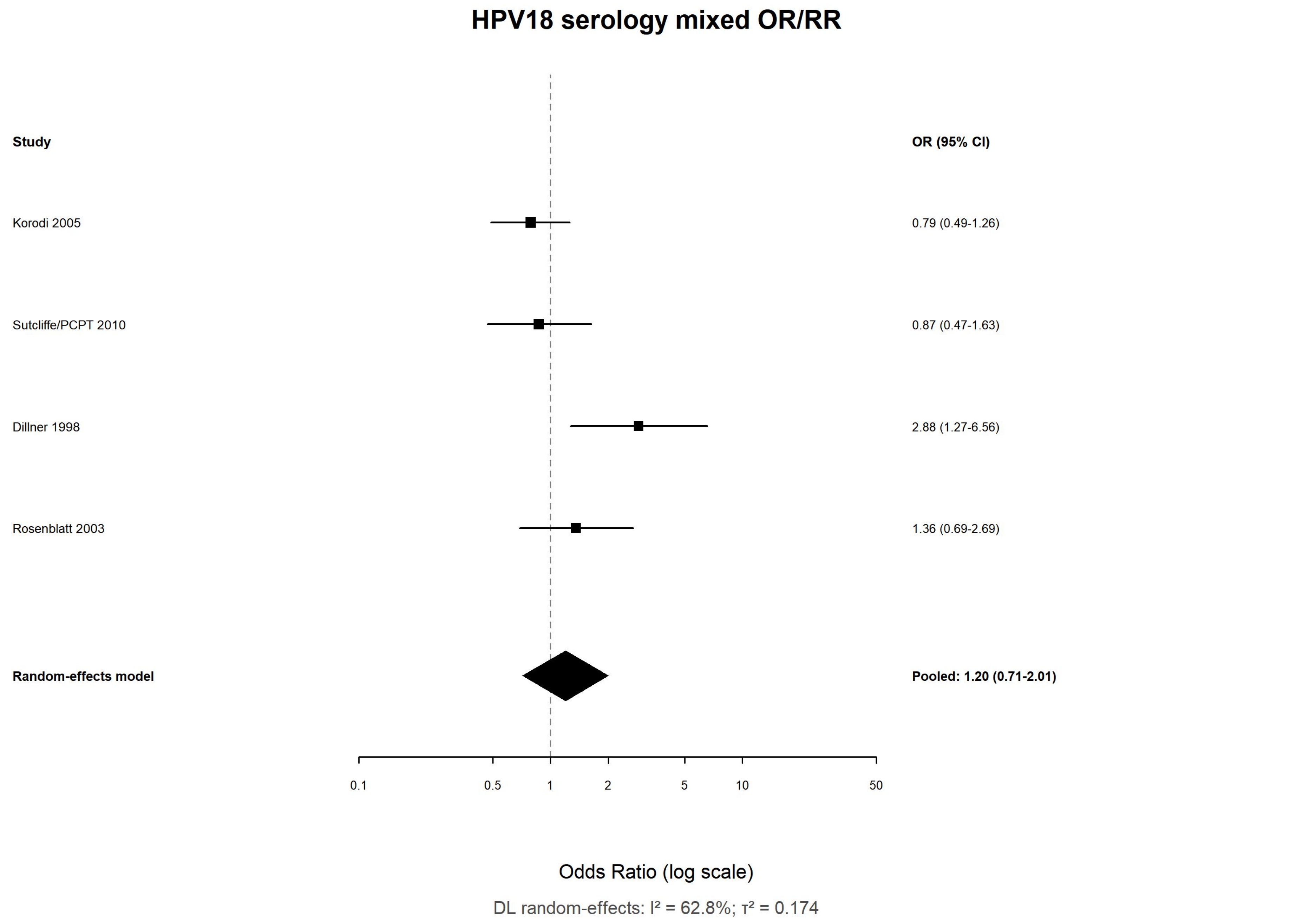
HPV18 serology mixed OR/RR
Study
OR (95% CI)
Korodi 2005
0.79 (0.49-1.26)
Sutcliffe/PCPT 2010
0.87 (0.47-1.63)
Dillner 1998
2.88 (1.27-6.56)
Rosenblatt 2003
1.36 (0.69-2.69)
Random-effects model
Pooled: 1.20 (0.71-2.01)
0.1
0.5
1
2
5
10
50
Odds Ratio (log scale)
DL random-effects: I² = 62.8%; τ² = 0.174

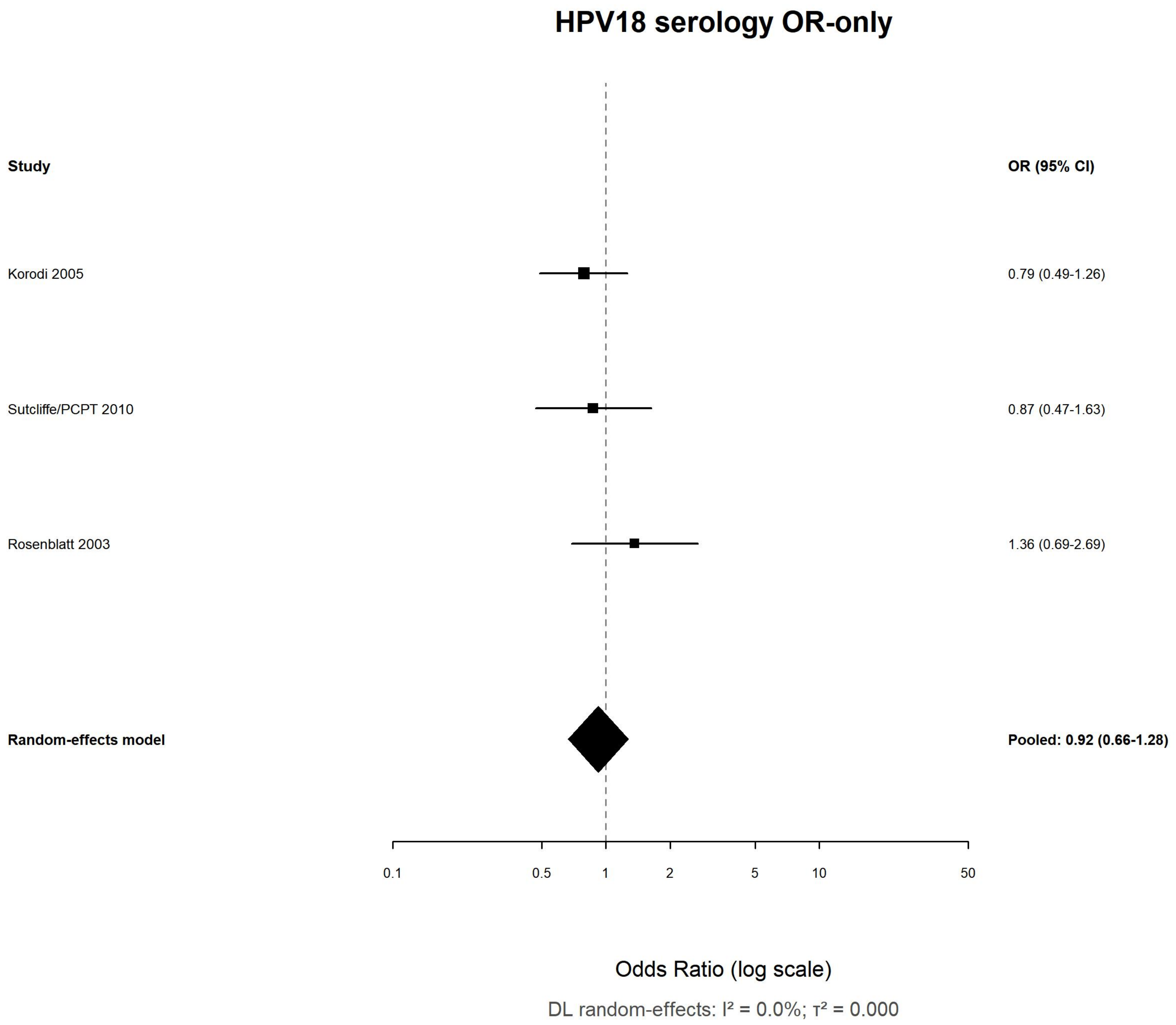
HPV18 serology OR-only
Study
OR (95% CI)
Korodi 2005
0.79 (0.49-1.26)
Sutcliffe/PCPT 2010
0.87 (0.47-1.63)
Rosenblatt 2003
1.36 (0.69-2.69)
Random-effects model
Pooled: 0.92 (0.66-1.28)
0.1
0.5
1
2
5
10
50
Odds Ratio (log scale)
DL random-effects: I² = 0.0%; τ² = 0.000

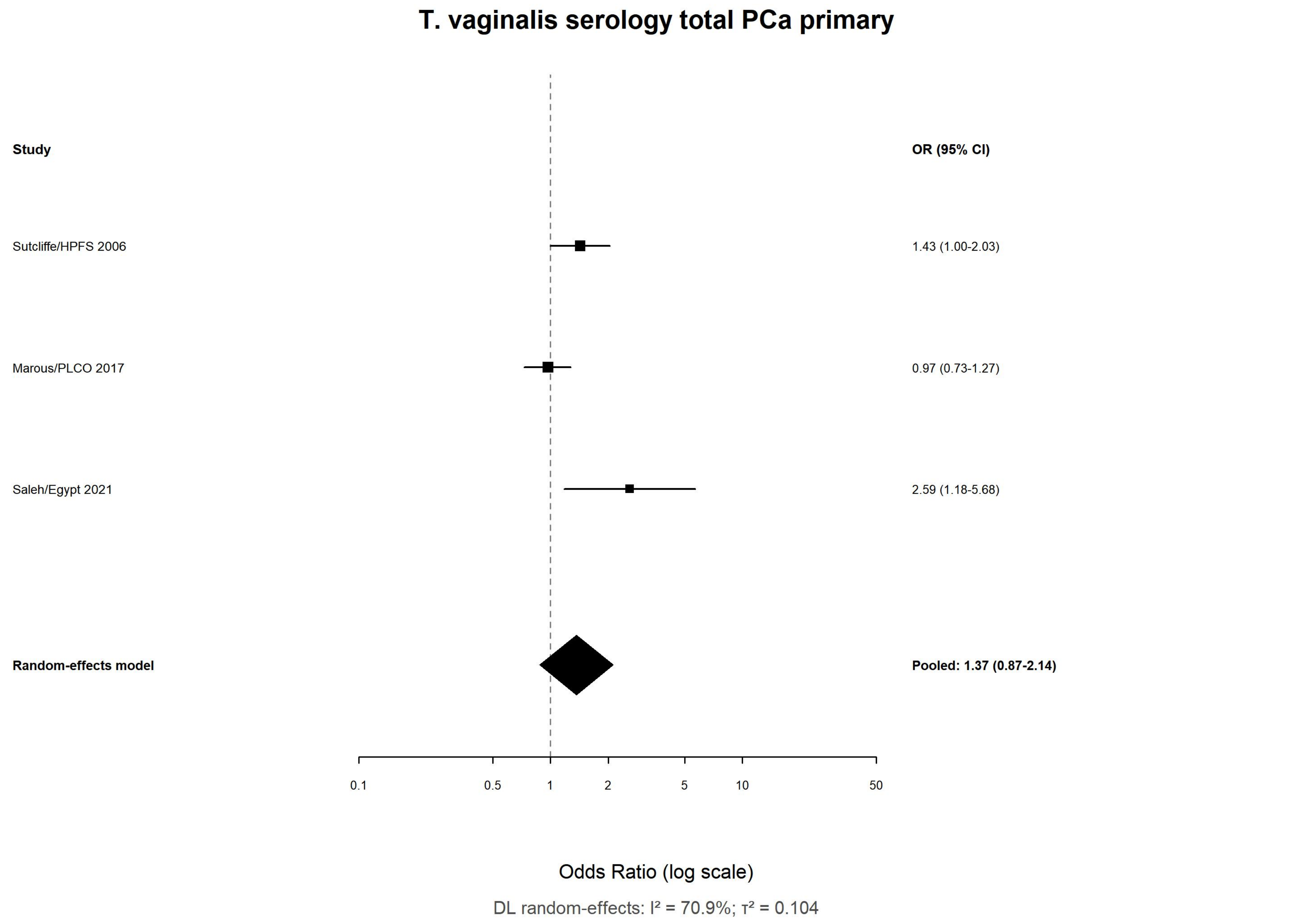
T. vaginalis serology total PCa primary
Study
OR (95% CI)
Sutcliffe/HPFS 2006
1.43 (1.00-2.03)
Marous/PLCO 2017
0.97 (0.73-1.27)
Saleh/Egypt 2021
2.59 (1.18-5.68)
Random-effects model
Pooled: 1.37 (0.87-2.14)
0.1
0.5
1
2
5
10
50
Odds Ratio (log scale)
DL random-effects: I² = 70.9%; τ² = 0.104

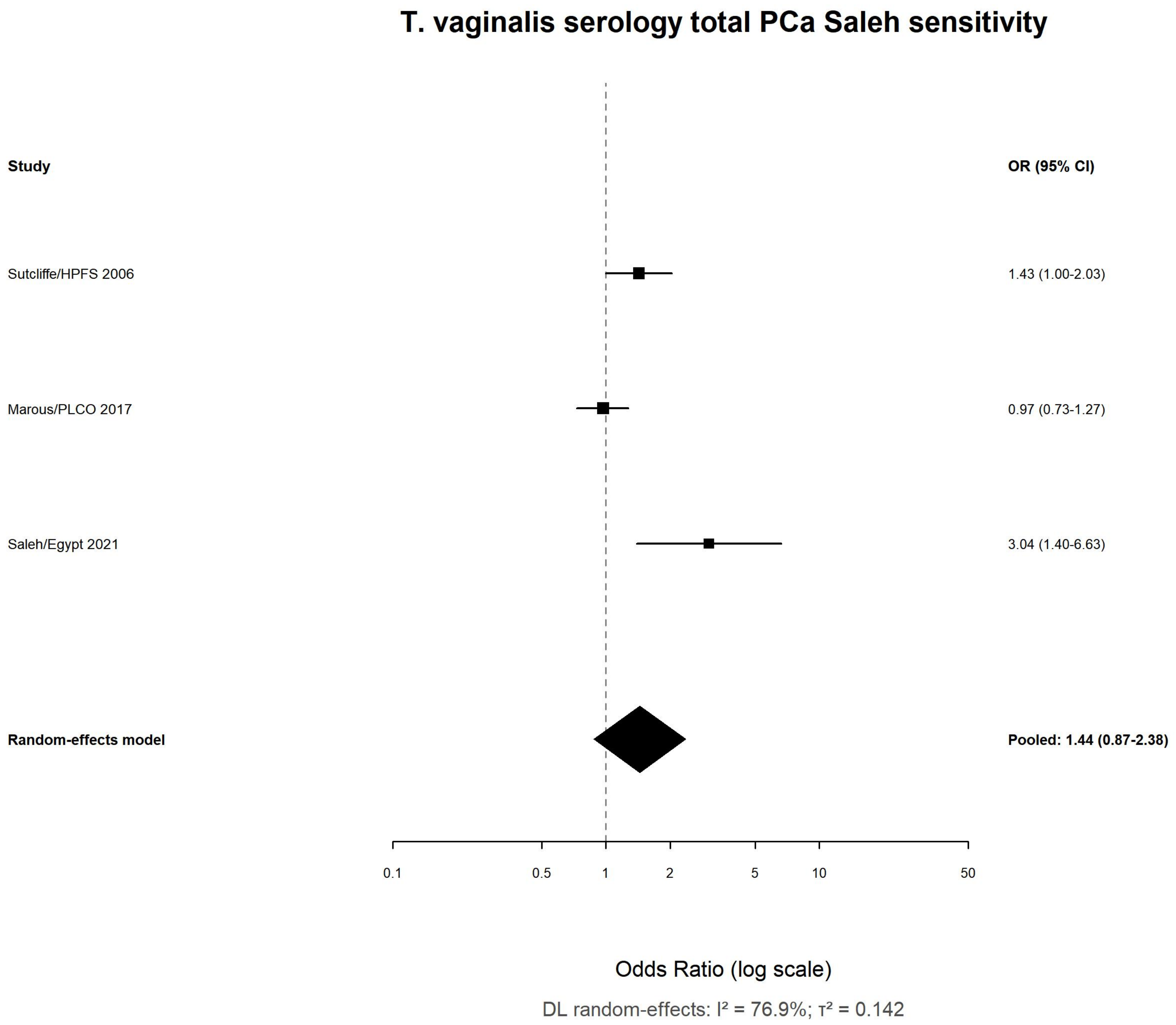

T. vaginalis serology total PCa Saleh sensitivity
Study
OR (95% CI)
Sutcliffe/HPFS 2006
1.43 (1.00-2.03)
Marous/PLCO 2017
0.97 (0.73-1.27)
Saleh/Egypt 2021
3.04 (1.40-6.63)
Random-effects model
Pooled: 1.44 (0.87-2.38)
0.1
0.5
1
2
5
10
50
Odds Ratio (log scale)
DL random-effects: I² = 76.9%; τ² = 0.142

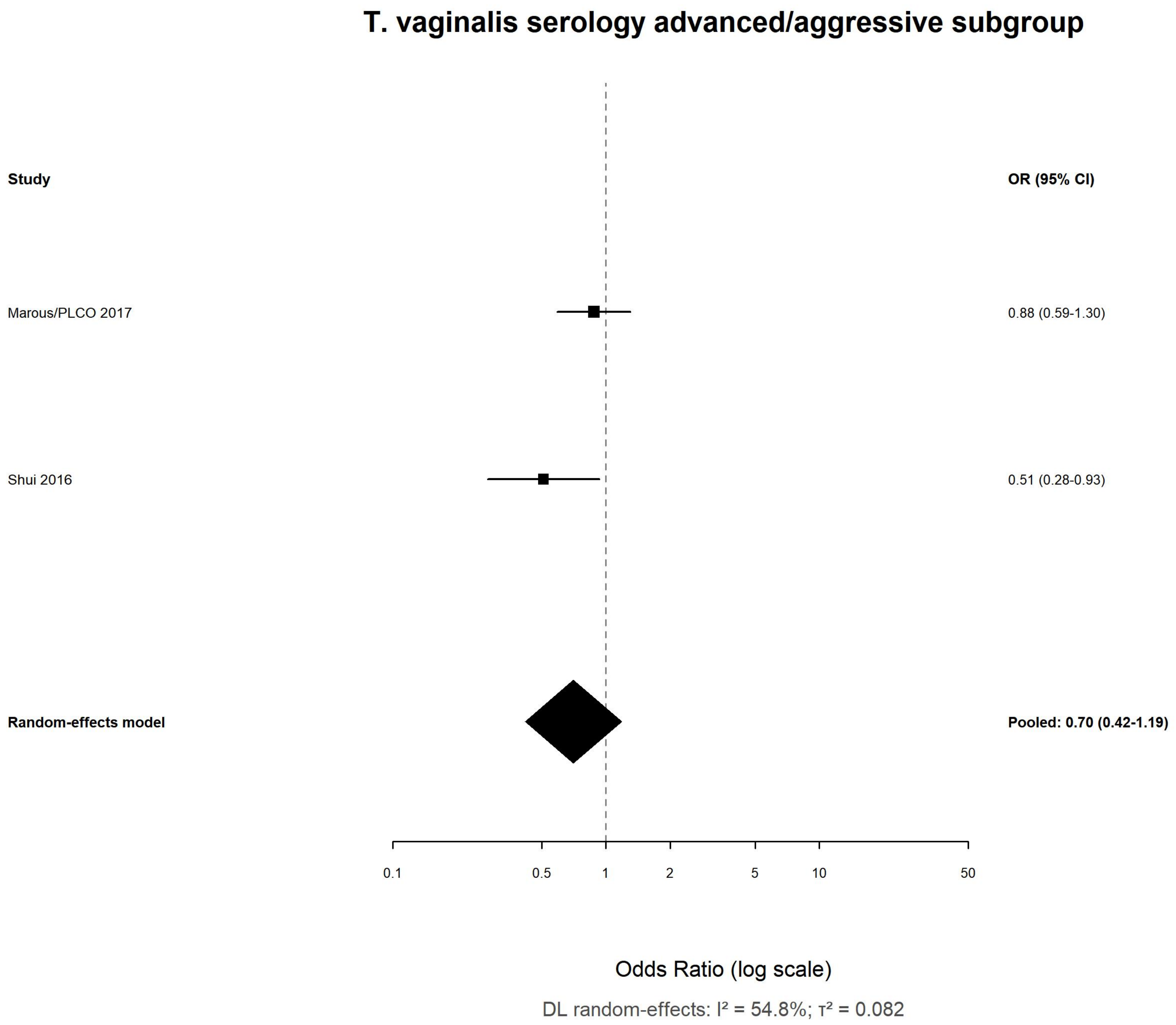
T. vaginalis serology advanced/aggressive subgroup
Study
OR (95% CI)
Marous/PLCO 2017
0.88 (0.59-1.30)
Shui 2016
0.51 (0.28-0.93)
Random-effects model
Pooled: 0.70 (0.42-1.19)
0.1
0.5
1
2
5
10
50
Odds Ratio (log scale)
DL random-effects: I² = 54.8%; τ² = 0.082

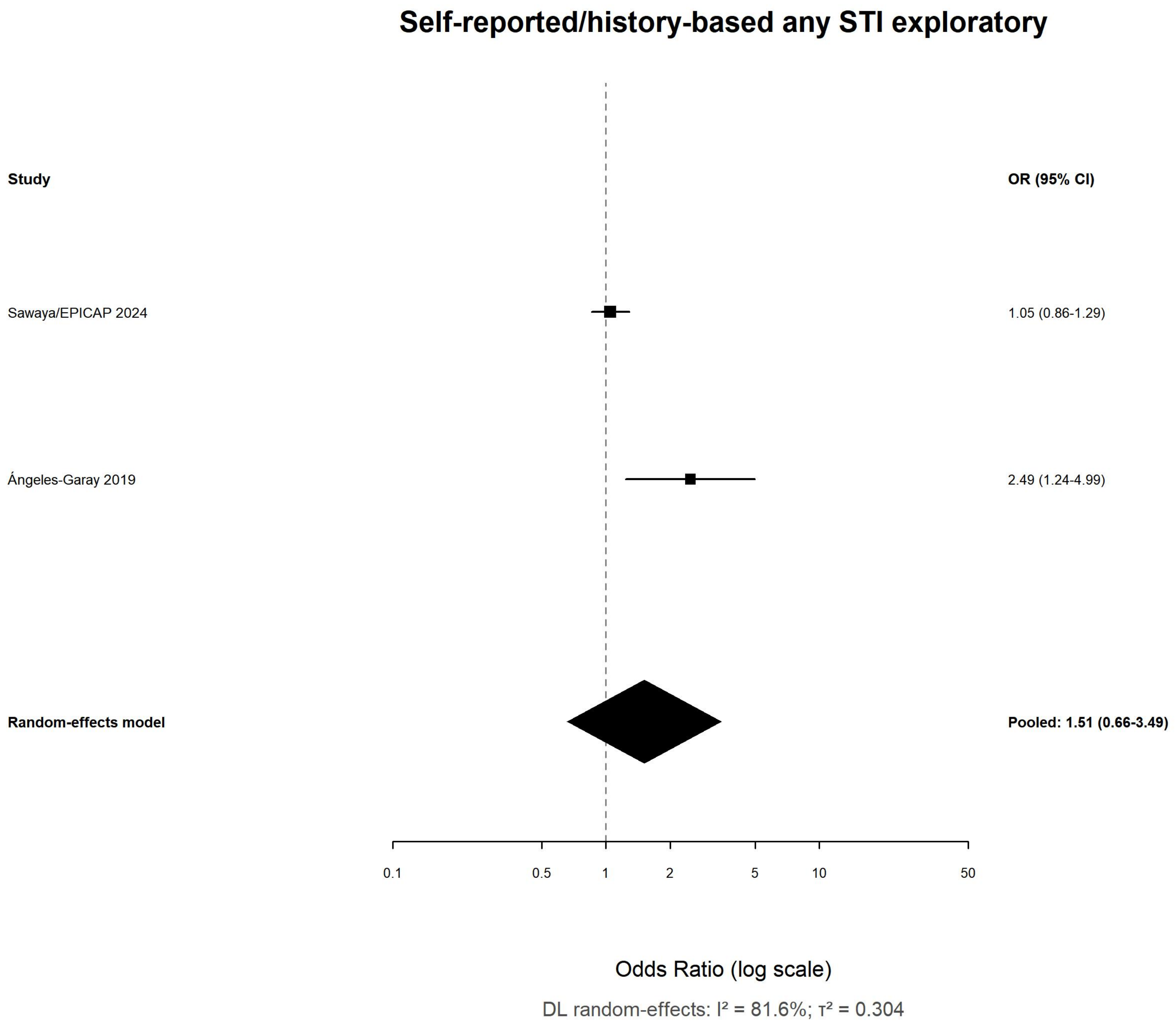

Self-reported/history-based any STI exploratory
Study
OR (95% CI)
Sawaya/EPICAP 2024
1.05 (0.86-1.29)
Ángeles-Garay 2019
2.49 (1.24-4.99)
Random-effects model
Pooled: 1.51 (0.66-3.49)
0.1
0.5
1
2
5
10
50
Odds Ratio (log scale)
DL random-effects: I² = 81.6%; τ² = 0.304

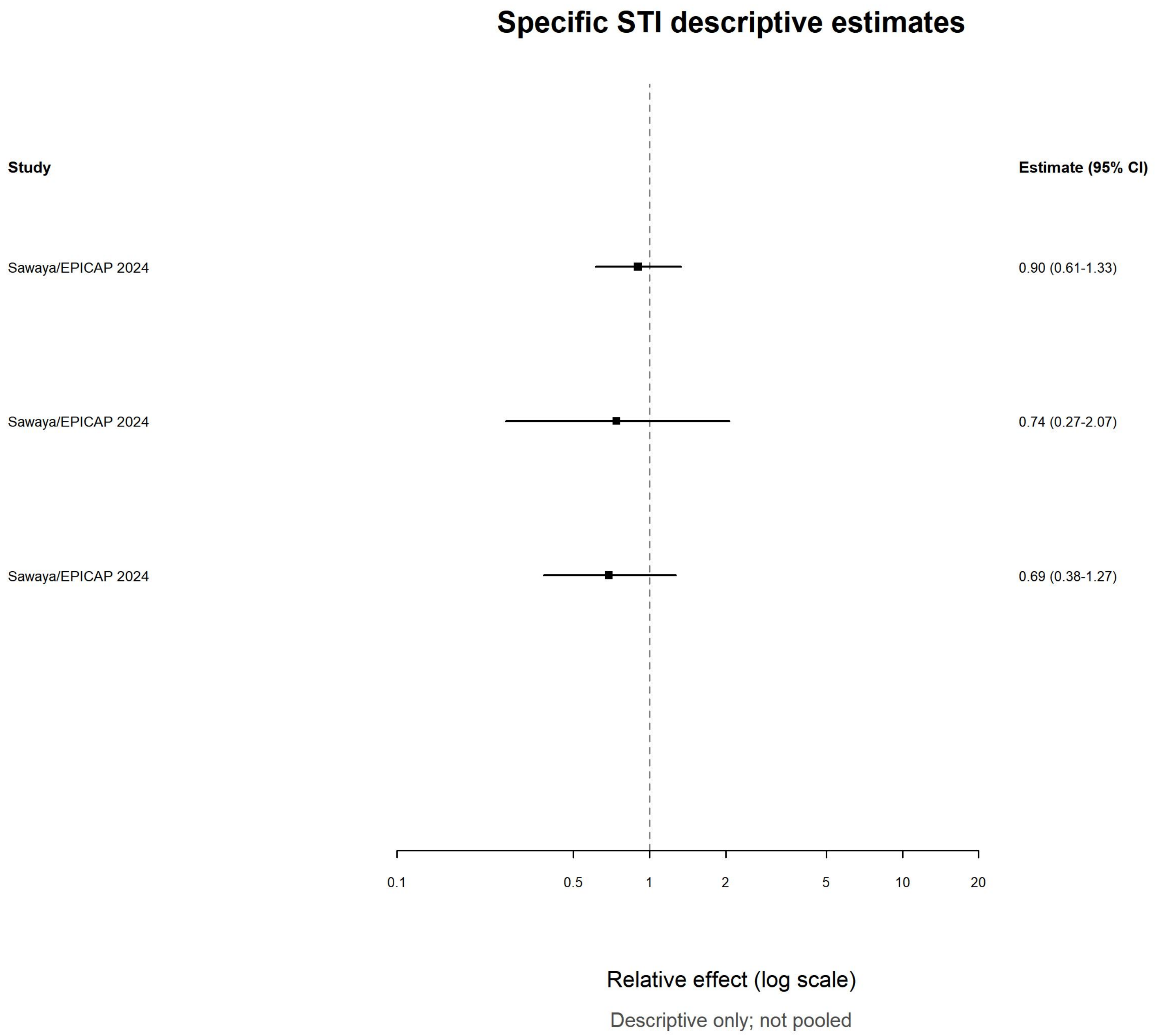
Specific STI descriptive estimates
Study
Estimate (95% CI)
Sawaya/EPICAP 2024
0.90 (0.61-1.33)
Sawaya/EPICAP 2024
0.74 (0.27-2.07)
Sawaya/EPICAP 2024
0.69 (0.38-1.27)
0.1
0.5
1
2
5
10
20
Relative effect (log scale)
Descriptive only; not pooled

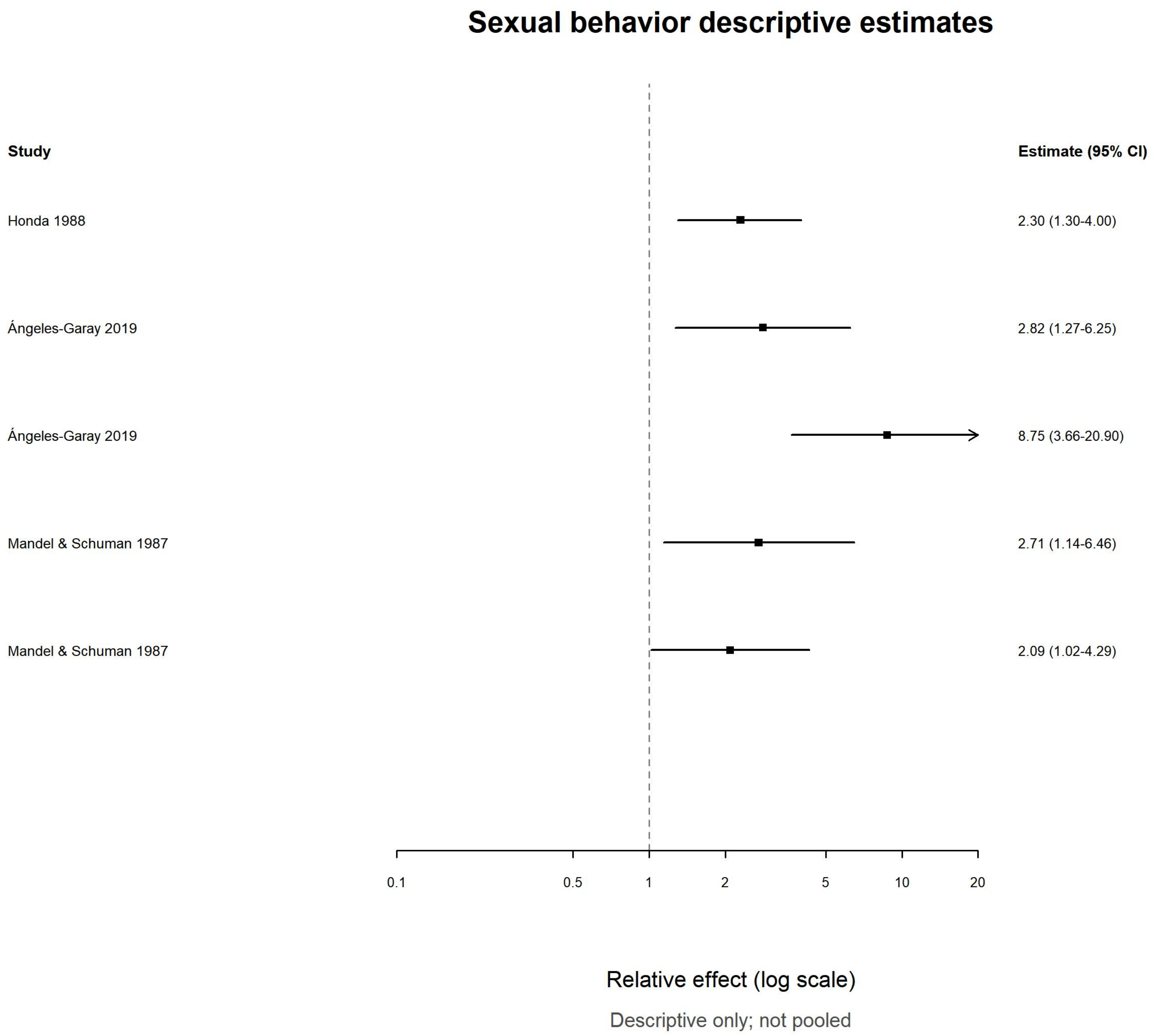
Sexual behavior descriptive estimates
Study
Estimate (95% CI)
Honda 1988
2.30 (1.30-4.00)
Ángeles-Garay 2019
2.82 (1.27-6.25)
Ángeles-Garay 2019
8.75 (3.66-20.90)
Mandel & Schuman 1987
2.71 (1.14-6.46)
Mandel & Schuman 1987
2.09 (1.02-4.29)
0.1
0.5
1
2
5
10
20
Relative effect (log scale)
Descriptive only; not pooled

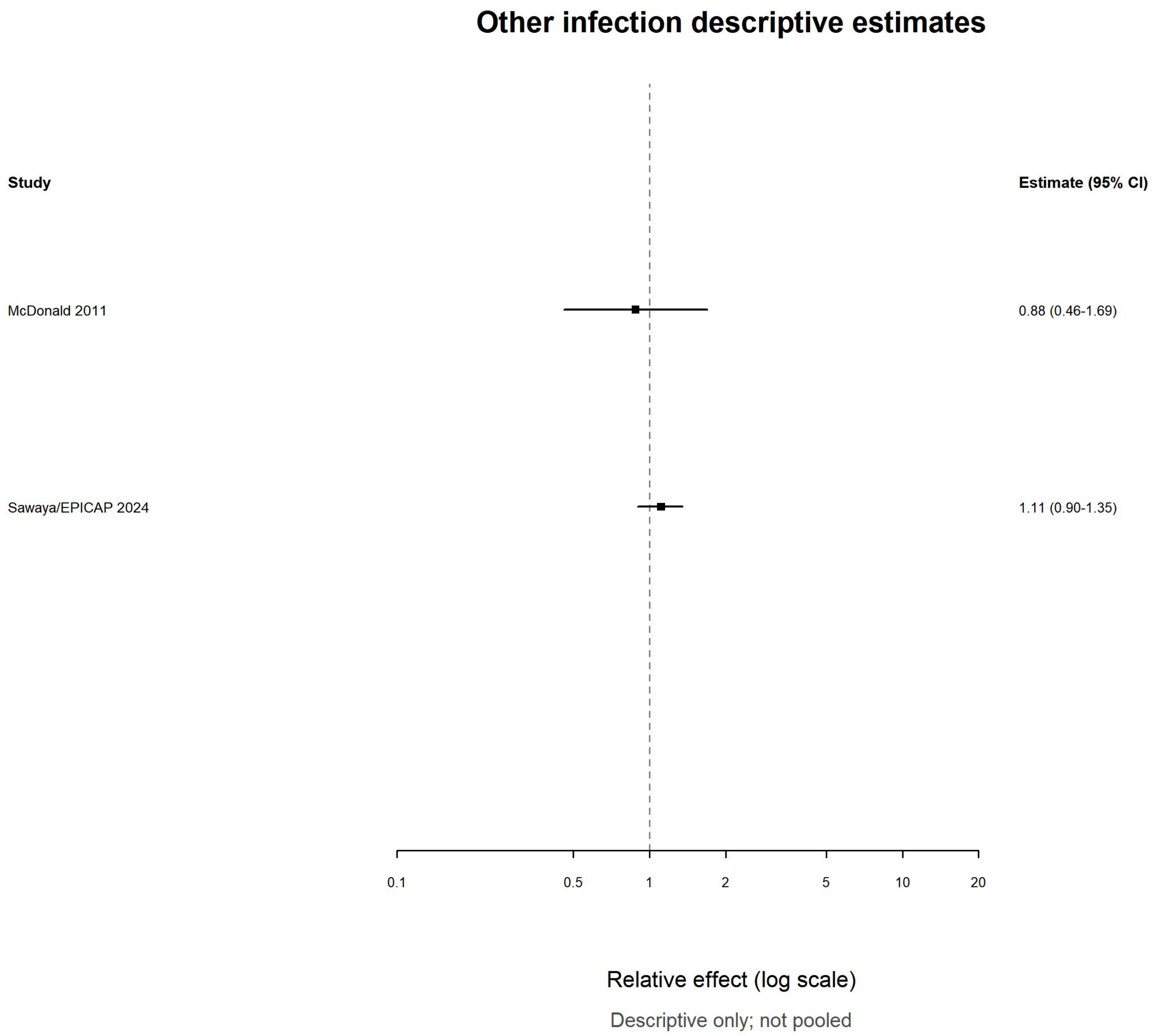

Other infection descriptive estimates
Study
Estimate (95% CI)
McDonald 2011
0.88 (0.46-1.69)
Sawaya/EPICAP 2024
1.11 (0.90-1.35)
0.1
0.5
1
2
5
10
20
Relative effect (log scale)
Descriptive only; not pooled

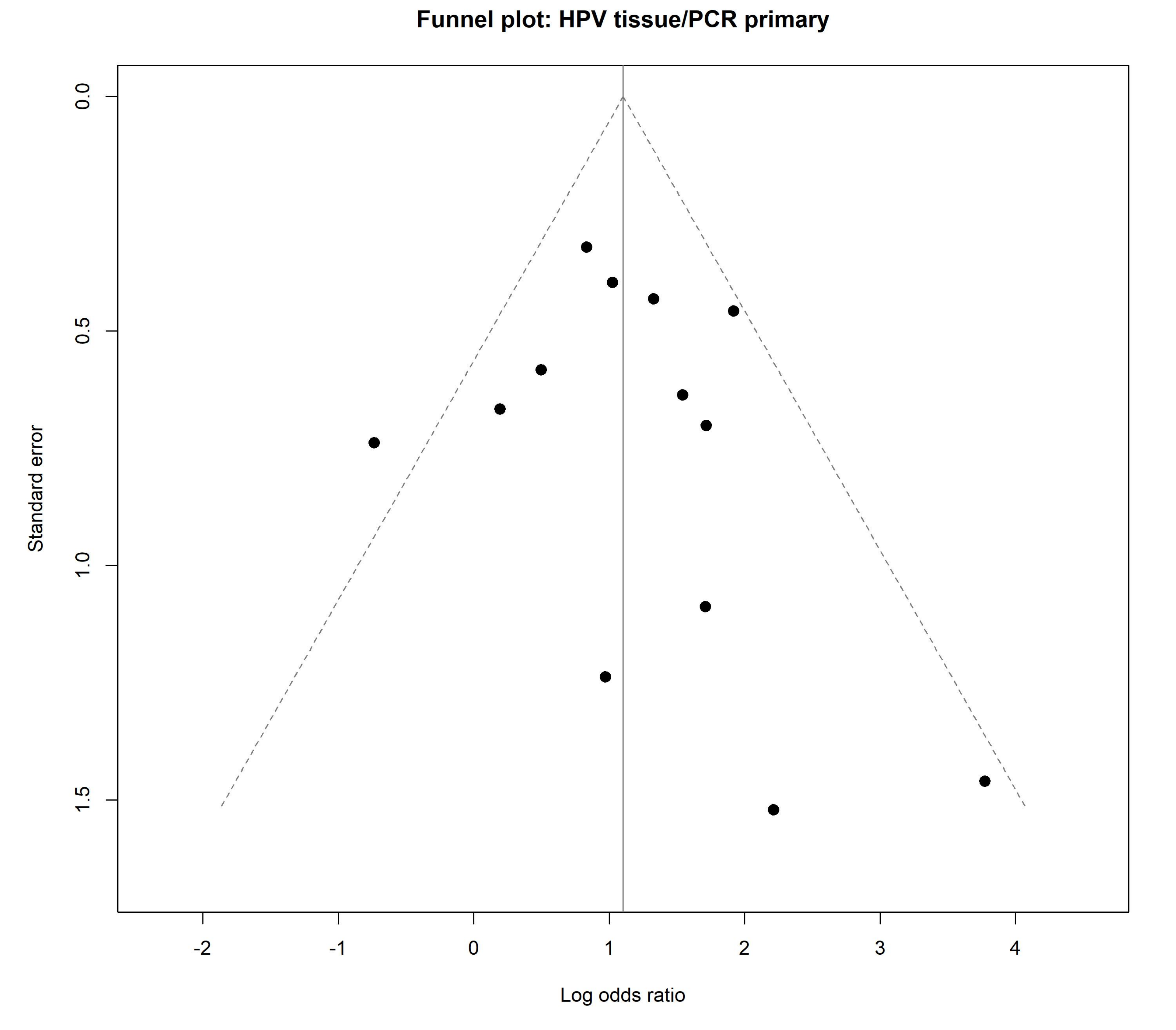

Funnel plot: HPV tissue/PCR primary
Standard error
0.0
0.5
1.0
1.5
-2
-1
0
1
2
3
4
Log odds ratio
